# Antioxidant and Anti-Inflammatory Activity of *Cynanchum acutum* L. Isolated Flavonoids Using Experimentally Induced Type 2 Diabetes Mellitus: Biological and In Silico Investigation for NF-κB Pathway/miR-146a Expression Modulation

**DOI:** 10.3390/antiox10111713

**Published:** 2021-10-27

**Authors:** Reda F. A. Abdelhameed, Amany K. Ibrahim, Mahmoud A. Elfaky, Eman S. Habib, Mayada I. Mahamed, Eman T. Mehanna, Khaled M. Darwish, Dina M. Khodeer, Safwat A. Ahmed, Sameh S. Elhady

**Affiliations:** 1 Department of Pharmacognosy, Faculty of Pharmacy, Suez Canal University, Ismailia 41522, Egypt; reda.abdelhameed@pharm.suez.edu.eg (R.F.A.A.); emy_197@hotmail.com (E.S.H.); mayada_isamedin@pharm.suez.edu.eg (M.I.M.); safwat_aa@yahoo.com (S.A.A.); 2 Department of Pharmacognosy, Faculty of Pharmacy, Galala University, New Galala 43713, Egypt; 3 Department of Natural Products, Faculty of Pharmacy, King Abdulaziz University, Jeddah 21589, Saudi Arabia; melfaky@kau.edu.sa (M.A.E.); ssahmed@kau.edu.sa (S.S.E.); 4 Centre for Artificial Intelligence in Precision Medicines, King Abdulaziz University, Jeddah 21589, Saudi Arabia; 5 Department of Biochemistry, Faculty of Pharmacy, Suez Canal University, Ismailia 41522, Egypt; 6 Department of Medicinal Chemistry, Faculty of Pharmacy, Suez Canal University, Ismailia 41522, Egypt; khaled_darwish@pharm.suez.edu.eg; 7 Department of Pharmacology, and Toxicology, Faculty of Pharmacy, Suez Canal University, Ismailia 41522, Egypt; dina_khoudaer@pharm.suez.edu.eg

**Keywords:** Apocynaceae, *Cynanchumacutum*, flavonoids, miR-146a, nuclear factor kappa B, tumor necrosis factor-alpha, molecular modeling

## Abstract

*Cynanchum acutum* L. is a climbing vine that belongs to the family Apocynaceae. Using different chromatographic techniques, seven compounds were isolated from the methanolic extract of the plant. The isolated compounds include six flavonoid compounds identified as rutin (**1**), quercetin-3-*O*-neohesperidoside (**2**), quercetin-3-*O*-*β*-galactoside (**3**), isoquercitrin (**4**), quercetin (**5**), and kaempferol 3-*O*-*β*-glucoside (**6**), in addition to a coumarin, scopoletin (**7**). The structures of the compounds were elucidated based on 1D NMR spectroscopy and high-resolution mass spectrometry (HR-MS), and by comparison with data reported in the literature. The first five compounds were selected for in vivo investigation of their anti-inflammatory and antioxidant properties in a rat model of type 2 diabetes. All tested compounds significantly reduced oxidative stress and increased erythrocyte lysate levels of antioxidant enzymes, along with the amelioration of the serum levels of inflammatory markers. Upregulation of miR-146a expression and downregulation of nuclear factor kappa B (NF-κB) expression were detected in the liver and adipose tissue of rats treated with the isolated flavonoids. Results from the biological investigation and those from the validated molecular modeling approach on two biological targets of the NF-κB pathway managed to highlight the superior anti-inflammatory activity of quercetin-3-*O*-galactoside (**3**) and quercetin (**5**), as compared to other bioactive metabolites.

## 1. Introduction

The family Apocynaceae, commonly known as the dogbane family, is a large family of flowering plants that includes forest trees, shrubs, perennial herbs, succulent herbs, and vines. Many Apocynaceae plants have milky latex, and many species are poisonous. Several plants belonging to Apocynaceae were traditionally used as arrow poisons and dog poisons, hence the name “dogbane” [1]. The subfamily Asclepiadoideae, formerly family Asclepiadaceae, is a subfamily of Apocynaceae that contains 2900 species distributed among 348 genera [2]. The *Cynanchum* genus previously belonged to Asclepiadaceae, but is now classified under the Apocynaceae. Plants belonging to genus *Cynanchum* have traditionally been used in folk medicine as antifebrile, antitussive, diuretic, expectorant, anticonvulsant, analgesic, and tonic agents [3]. Pharmacological studies have reported various uses for *Cynanchum* plants. The dried whole plant of *C. paniculatum* was reported to possess potent anti-inflammatory activity [4]. The essential oil of *C. chinense* showed potent antibacterial and antioxidant activities [5]. The dried whole *C. atratum* plant was reported to have acetylcholinesterase inhibitory activity [6]. *C. stauntonii* extract showed potent airway smooth muscle relaxant effects [7]. *Cynanchum acutum* L. is a climbing vine that is native to Europe, Africa, and Asia. It commonly grows in Egypt and is known among natives as olliq, modeid, or libbein [8,9]. The alcoholic extract of the leaves of *C. acutum* was reported to have insecticidal [10], antidiabetic [11], antioxidant [11], antibacterial [12], anticancer [13], anti-inflammatory, analgesic, and antipyretic [12] effects. The extract also had a protective role against carbon tetrachloride-induced hepatotoxicity in rats [14]. Phytochemical investigation of *Cynanchum* plants revealed the presence of several natural compounds, including triterpenes [15], carbohydrates [16], long-chain fatty acid esters [17], alkaloids [18], flavonoids [19], and coumarins [20].

Several flavonoids were isolated from the methanolic extract of *C. acutum*. Their structures were established as quercetin 3-*O*-*β*-galacturonopyranoside, quercetin 7-*O*-*β*-glucopyranoside, tamarixtin 3-*O*-*β*-galacturonopyranoside, kaempferol 3-*O*-*β*-galacturonopyranoside, 8-hydroxyquercetin 3-*O*-*β*-galactopyranoside, tamarixtin 3-*O*-*α*-rhamnopyranoside, and tamarixtin 7-*O*-*α*-arabinopyranoside [11]. Flavonoids are known to exhibit strong antioxidant and antitumor [21] activities. Simple coumarins that have been isolated from the aerial parts of *C. acutum* include scopoletin and scoparone [20]. Flavonoids are well known to have strong antioxidant and anti-inflammatory properties, and are widely used as nutraceuticals and pharmaceutical components in a variety of cases [22]. The antioxidant and anti-inflammatory properties of quercetin have been proven in type 2 diabetes (T2DM) and obesity [23]. Similarly, isoquercetin and rutin were reported to ameliorate the oxidative stress and inflammation in the liver of T2DM experimental rats [24,25]. Rutin also had a glucose-lowering effect in a diabetic mouse model [26]. Several mechanisms were suggested to explain the biological activities of the previous compounds; however, further studies are required to provide novel insights on the molecular mechanisms that promote the beneficial actions of those flavonoids and their derivatives.

The aim of this study was to investigate the chemical profile of *C. acutum* growing in Egypt, and to assess the antioxidant and anti-inflammatory properties of its isolated flavonoids in experimentally induced T2DM, along with investigating the molecular mechanisms of those effects by means of biochemical measurements and molecular modeling.

## 2. Materials and Methods

### 2.1. Plant Material

Samples of the whole plant of *C. acutum* were collected from three different locations in Suez Canal University: Faculty of Pharmacy Garden, Faculty of Commerce garden, and Faculty of Nursing garden during the month of October 2019. The plant was kindly authenticated by Professor Sayeda Gamal Eldin, Professor of Botany, Faculty of Science, Suez Canal University. A voucher specimen (# SAA-157) was deposited in the herbarium section of the Pharmacognosy Department, Faculty of Pharmacy, Suez Canal University, Egypt.

### 2.2. Reagents and Instrumentation

Analytically pure reagents (dichloromethane, ethyl acetate, methanol and *n*-butanol) for the extraction and isolation were purchased from Sigma Aldrich (Bremen, Germany). Thin layer chromatography (TLC) analysis was carried out on aluminum-backed plates pre-coated with silica gel F254 (20 × 20 cm; 200 µm; 60 Å (Merck™, Darmstadt, Germany). Chromatographically pure reagents for HPLC and HR-ESI-MS (DI-water, methanol, acetonitrile) were purchased from TEDIA Company Inc. (Fairfield, CA, USA). Sephadex^®^ LH-20 (Sigma Aldrich, Bremen, Germany) and silica gel 60/230–400 µm mesh size (Whatman™, Sanford, ME, USA) were used for column chromatography. The pre-column used for HR-ESI-MS was In-Line filter disks (Phenomenex, 0.5 µm × 3.0 mm) and the column was X select HSS T3 (Waters, 2.5 µm, 2.1 × 150 mm); the flow rate was 0.3 mL/min. Data processing was performed via MS-DIAL3.52. Feature (peaks) extraction from total ion chromatogram was achieved using master view software, 1H and 13C NMR data were obtained with a Bruker Avance III HD 400 spectrometer operating at 400 MHz for 1H, and 100 MHz for 13C. Both 1H and 13C NMR chemical shifts are expressed in *δ* values and coupling constants are given in Hertz (Hz).

### 2.3. Extraction and Isolation

After collection, the plant (10 kg) was dried, powdered, and extracted with methanol (4 × 5 L). The extract was dried under vacuum using rotary evaporator to give 531 g of greenish methanolic extract. The dried extract was suspended in 1.5 L of distilled water, then extracted with 4 L each of hexane, chloroform, ethyl acetate and butanol, successively. Each of the four extracts was dried under vacuum using rotary evaporator to yield 170 g, 80 g, 62 g, and 126 g of hexane (RH), chloroform (RC), ethyl acetate (RE), and butanol (RB) dried extracts, respectively. The ethyl acetate extract (RE, 62 g) was fractionated using a hexane/EtOAc gradient until 100% EtOAc, followed by an EtOAc/MeOH gradient, until 100% MeOH.

The fraction eluted using 100% MeOH (1.8 g) was purified by Sephadex LH-20 using methanol as an eluent to yield compound **1** (50 mg). The fraction eluted using 50% MeOH in EtOAc (3 g) was purified by a Sephadex LH-20 column twice, using methanol as an eluent to yield compound **2** (150.7 mg). The fraction eluted using 35% MeOH in EtOAc (1.5 g) was subjected to column chromatography using a chloroform/MeOH gradient until 100% MeOH, followed by a MeOH/H_2_O gradient until 10% H_2_O/MeOH. TLC was used for monitoring the subfractions, and similar subfractions were pooled together and evaporated to dryness to afford compound **3** (630 mg).

The fraction eluted using 20% MeOH in EtOAc was concentrated to afford 414.6 mg of a green residue. It was purified on a Sephadex LH-20 column using methanol as an eluent to yield 7 subfractions. Subfraction 5 showed one spot on the TLC plate (compound **4**, 56 mg). Subfraction 4 (50.5 mg) was further purified on Sephadex LH-20 to yield compound **6** (15.2 mg).

The fraction eluted using 80% EtOAc in hexane (300 mg) was purified by a Sephadex LH-20 column twice, using methanol as an eluent to yield compound **5** (100.7 mg). The fraction eluted using 20% EtOAc in hexane (400 mg) was purified by a Sephadex LH-20 column using methanol as an eluent. TLC was used for monitoring the subfractions and similar subfractions were pooled together and evaporated to dryness under reduced pressure to afford 61.9 mg of a greenish blue residue. Further purification was carried out by preparative chromatography on TLC plates and by using Sephadex LH-20 to afford compound **7** (8.6 mg).

### 2.4. Experimental Animals

A total of one hundred and twelve (112) male albino rats (150 ± 17 g) were purchased from the Egyptian Organization for Biological Products and Vaccines (Cairo, Egypt). They were left to acclimatize for seven days at 25 °C, with free access to standard diet and tap water and a normal dark/light a cycle. The animals were kept and used according to the guidelines of the National Research Council (2011). The study protocol was approved by Ethical Committee of Faculty of Pharmacy, Suez Canal University (202102RA1).

#### 2.4.1. In Vivo Biological Evaluation of the *C. acutum* Crude Extract and Fractions

The in vivo study was carried out in two consecutive stages. The first involved investigation of the anti-diabetic and anti-obesity effects of the crude extract of *C. acutum* and four of its fractions, where 56 rats were divided randomly into seven groups (*n* = 8). One normal group was fed a normal pellet diet, and all of the other six groups were fed a high fat diet (HFD) [i.e., 87.7% standard diet (*w/w*), 10% pork fat (*w/w*), 2% cholesterol (*w/w*) and 0.3% bile salt (*w/w*)] [27,28]. After 10 weeks of receiving a high fat diet, the rats were administered a single intra-peritoneal (i.p) dose of Streptozotocin (STZ) dissolved in citrate buffer (30 mg/kg). Ten days after injection of STZ, rats were fasted overnight and fasting blood glucose (FBG) was measured, where the rats with FBG > 200 mg/dL were considered diabetic and were assigned into: control HDF + STZ group (untreated), crude extract group, hexane fraction group, chloroform fraction group, ethyl acetate fraction groups, and butanol fraction group. The crude extract and all the fractions were dissolved into tween 80 and were given by oral gavage to the rats at a dose of 250 mg/kg/day for 14 consecutive days.

At the end of the experiment, the rats were weighed to determine the percentage increase in body weight compared to the starting weight of each rat, and they were fasted overnight. Blood samples were collected from the tail vein and rats were euthanized under ketamine anesthesia. A portion of the blood was collected on EDTA anti-coagulant, and plasma was separated by centrifugation at 3000 rpm for 15 min at 4 °C. FBG was determined in plasma calorimetrically (Biodiagnostic, Cairo, Egypt), and fasting plasma insulin was determined by ELISA (Cat No MBS045315) (MyBioSource, San Diego, CA, USA). Homeostasis model assessment-insulin resistance (HOMA-IR) was calculated by the equation: HOMA-IR = [FBG (mg/dL) * fasting plasma insulin (μIU/mL))/405] [29], and quantitative insulin sensitivity check index (QUICKI) was calculated by the equation: QUICKI = 1/(log FBG + log fasting plasma insulin) [30].

The other portion of blood was collected in plain tubes and used for separation of serum by centrifugation at 3000 rpm for 15 min, and at 4 °C. Serum was used for determination of the liver enzymes: alanine aminotransferase (ALT) and aspartate aminotransferase (AST), calorimetrically (Diamond, Egypt). Lipid profile was also determined calorimetrically in serum, including: triglycerides (TG), total cholesterol (TC), low density lipoprotein-cholesterol (LDL-C), and high density lipoprotein-cholesterol (HDL-C) (Biodiagnostic, Egypt).

Liver was isolated from the rats, washed with ice-cold saline and weighed to determine the liver index ((liver weight/body weight) × 100]. Subcutaneous and visceral (including epididymal) white adipose tissue was also dissected, washed, and weighed to calculate the adipose tissue index (adipose tissue weight/body weight) × 100).

#### 2.4.2. In Vivo Study of the Effects of the Isolated Compounds of *C. acutum*

The second stage of the in vivo study was carried out to investigate the anti-oxidative and anti-inflammatory effects of five compounds that were isolated from the most active fraction (ethyl acetate fraction). Fifty-six (56) rats were randomly divided into seven groups (*n* = 8). The first group was considered as the normal group, and T2DM was induced in all other seven groups, as described above. Diabetic rats were divided into: control (HFD + STZ) untreated group, and five groups were treated with: C1 (rutin), C2 (quercetin 3-*O*-neohesperdoside), C3 (quercetin-3-*O*-*β*-galactoside), C4 (isoquercetin), and C5 (quercetin). The other two compounds, kaempferol 3-*O*-*β*-glucoside and scopoletin, were not tested due to their relatively small quantities. All investigated compounds were dissolved in tween 80 and administered by oral gavage at a dose of 10 mg/kg/day for 21 consecutive days. The food intake of the rats was recorded daily through calculating the difference between the amount of food offered to the rats and the amount remaining in the feeder next day.

At the end of the experiment, rats were weighed and fasted overnight, blood was collected where plasma and serum were separated as previously described and stored at -20°C. Anesthetized rats were euthanized, and liver and adipose tissue were isolated for determination of the liver index, adipose tissue index, and for histopathological examination. Portions of the visceral white adipose tissue and the liver tissue were frozen for further assessment of biochemical markers.

Additionally, liver lipids were extracted according to the modified Folch method that was described by Mopuri et al. [31], and the levels of TC and TG in the liver were measured by enzymatic colorimetric kits (Biodiagnostic, Egypt).

#### 2.4.3. Determination of MDA, Antioxidant Enzymes and Inflammatory Markers

Reduced glutathione (GSH), superoxide dismutase (SOD), and catalase were assessed in the erythrocyte lysate that was collected from stored plasma samples. The leucocytes buffy layer was removed, and erythrocytes were lysed by tris-buffered ammonium chloride, followed by centrifugation at 10000 rpm for 15 min at 4°C. GSH (Cat No MBS724319), SOD (MBS036924), and catalase (MBS726781) were determined by ELISA kits (MyBioSource, San Diego, CA, USA). Levels of malondialdehyde (MDA) (Cat No MBS738685), tumor necrosis factor-alpha (TNF-α) (Cat No MBS2507393, and interleukin-6 (IL-6) (Cat No MBS726707) were also measured in serum by rat ELISA kits (MyBioSource, San Diego, CA, USA).

#### 2.4.4. Determination of the Expression of miR-146a, Inflammatory Markers and Adipokines in Liver and Adipose Tissues by Quantitative Real-Time PCR

Total RNA, including miRNA, was isolated from both the adipose and hepatic tissues by QiagenmiRNeasy Mini kit (Cat No 217004) (Qiagen, Hilden, Germany). The concentration and purity of isolated RNA were assessed by NanoDrop spectrophotometer (Thermo Fisher Scientific, Waltham, MA, USA).

For quantification of miR-146a in the adipose and liver tissues, TaqMan^®^ microRNA reverse transcription kit (Cat No 4366596), Taqman microRNA assays (Cat No 4427975) and Taqman universal master mix (Cat No 4440043) (Applied Biosystems, Waltham, MA, USA) were used, where cDNA was produced according to the manufacturer’s instructions. U6B small nuclear RNA (RNU6B) was used as the endogenous control. The real-time PCR reaction mix consisted of: 4 μL of cDNA, 10 μLTaqman universal mater mix, 1 μLTaqman assay (20×) (miR-146a or RNU6B), and 5 μL nuclease-free water. The cycle was composed of 10 min at 95 °C for polymerase activation; followed by 40 cycles of denaturation at 95 °C for 15 s, and annealing at 60 °C for 60 s.

The expression of nuclear factor kappa B (NF-κB) and TNF-α was determined in both the liver and adipose tissue, while the expression of leptin and adiponectin was measured only in the adipose tissue using GoTaq^®^ 1-Step RT-qPCR System (Promega, Madison, WI, USA). Glyceraldehyde 3-phosphate dehydrogenase (GAPDH) was used as an endogenous control. The primers and annealing temperatures are listed in Table 1. The reaction consisted of: 4 μL RNA template, 0.4 μL GoScript™ RT mix for 1-step RT-qPCR, 1 μL of each of the forward and reverse primers, 10 μL GoTaq^®^ qPCR master mix, 0.31 μL supplemental CXR reference dye, and 3.29 μL nuclease-free water. The cycle involved 15 min at 37 °C for reverse transcription, inactivation of reverse transcriptase enzyme at 95 °C for 10 min, followed by 40 cycles of: denaturation at 95 °C for 10 s, annealing for 30 s, and extension at 72 °C for 30 s.

All real time PCR reactions were carried out in StepOnePlus™ Real-Time PCR thermal cycling instrument (Applied Biosystems, Waltham, MA, USA). ΔΔCt and fold change were calculated, and the results were expressed as log 2-fold change relative to the normal group (base line), where the value of the expression in the normal group was normalized to 1, and log 2 (1) = zero.

#### 2.4.5. Histopathological Examination

At the end of the experiment, after euthanizing the animals, the liver was dissected and washed with ice cold 0.9% NaCl solution. Hepatic and adipose tissues samples were taken 5 mm away from the edge of the largest hepatic lobe and part of white adipose tissue respectively, then fixed with 10% (v/v) formaldehyde, embedded in paraffin wax, stained with hematoxylin and eosin (H&E) stain for histopathological examinations. Histopathological semi-quantitative grading was performed for the hepatic steatosis, inflammation, and focal lytic necrosis (mostly caused by ballooning degeneration) [32]. Additionally, the diameters of 100 adipocyte spaces in randomly selected various microscopic fields were measured applying an image analysis system [ImageJ 1.45F] (National Institute of Health, Bethesda, MD, USA). All histological assessments and grading of both the liver and adipose tissue were performed by a professional pathologist, who was blinded to the experimental groups.

#### 2.4.6. Statistical Analysis

Results were expressed as mean ± SD. Comparison of the values in different groups was conducted by a one-way analysis of variance (ANOVA) followed by Bonferroni’s post hoc test for multiple comparisons. SPSS software version 21.0 (IBM, Armonk, NY, USA) was used to analyze the results. Statistically significant differences were considered at *p* < 0.05.

### 2.5. Molecular Modelling Investigation

Docking studies were conducted using the Molecular Operating Environment (MOE) service package for investigating the isolated compounds at the binding cavities of both biological targets; NF-κB RelB:p52 (PDB: 3DO7) heterodimer and hIKK (PDB: 4KIK). Routine docking protocol was processed as reported [33,34,35]. In brief, ligand preparation was proceeded through construction of the isolated compounds using the MOE Builder module through importing the PubChem-deposited SMILES strings; Quercetin (CID 5280343), quercetin-3-*O*-*β*-galactoside (CID 5281643), isoquercitrin (CID 5280804), quercetin-3-neohesperidoside (CID 5491657), and rutin (CID 5280805). The constructed ligands were energy-minimized under the Merck Molecular ForceField (MMFF), as implemented in the MOE program with a conjugate-gradient approach (2000 steps), until reaching 1 × 10^−3^ Kcal/Å gradient.

Biological targets were prepared through 3D-protonation, termini capping, and structural auto-corrections. Partial charges were assigned by the Amber99 forcefield being parameterized for the macromolecules, including both proteins and nucleic acid targets [36]. The binding sites were defined by MOE-Alpha SiteFinder, and refinement of the binding site was done for including important amino acids reported within the literature. For the NF-κB RelB:p52 heterodimer, the pharmacophoricκB DNA-specific binding site included Arg-117, Arg-119, Tyr-120, Cys-122, Glu-123, Arg-125, Ser-129, Arg-209, Lys-210, Gln-307, Lys-308, Arg-333, Gln-334 for the RelB monomer, as well as the homologous residues Arg-52, Arg-54, Tyr-55, Cys-57, Glu-58, Ser-61, His-62, Thr-142, Lys-143, Lys-252, Gln-254, Lys-255, Gln-284 for the p52 protomer [37]. Concerning the key residues included at the hIKK ATP-binding site, Leu-21-Thr-23, Asn-28, Val-29, Ala-42, Lys-44, Val-74, Met-96 to Asp-103, Asp-145, Lys-147, Glu-149, Asn-150, Val-152, Ile-165, Asp-166, and Tyr-169 were assigned as the pocket lining residues [38]. The hydrophobic-polar descriptors of the assigned active site were described using the Dummy atoms generated from the defined alphaspheres.

The B-factor values of the defined binding sites revealed the significant flexibility of its constituting amino acids, the thing that rationalized the application of induced-fitting docking protocol, where the receptor sidechains are allowed to flex during refinement of the poses. Throughout this protocol, generated ligand conformations were anchored within the binding site via the triangular matching technique, where they were ranked via London-dG score function. The refinement process was then proceeded for sets of 10 poses/ligand, followed by a rescoring protocol using the generalized born-solvation model/weighted surface-area (GBVI-WSA)-dG forcefield. This latter scoring module relies on Coulombic’s potentials, solvation electrostatics, ligand-protein van der Waals scores, exposure-weighed surface area, and loaded partial charges [39]. Docking simulation was done in triplicates, and data are represented as mean ± SD. The validity of adopted docking protocol was confirmed via a co-crystallized ligand (KSA; PDB: 4KIK) redocking approach at target’s binding site. Interestingly, the redocked co-crystallized ligand showed a root-mean-square deviation (RMSD) = 0.80 ± 0.12 Å, ensuring the validity of this molecular modelling protocol. This was rationalized, since both the adopted algorithms and parameters were considered sufficient for determining the best ligand’s binding mode when depicting redocking RMSD values below 2 Å [40]. Assessing docking pose validity and selection for the investigated ligands were done when obtaining significant interactions with key pocket amino acids and furnishing RMSD values around the 2.0 Å cut-off. PyMol-Schrodinger v2.0.6 graphical software was used for analysing and graphical representation of target-ligand binding interactions.

## 3. Results and Discussion

### 3.1. Isolated Compounds ***1**–**7***

Seven compounds (Figure 1) were isolated and purified from the fractions of the crude extract of *C. acutum*. The compounds were identified as rutin **1** [41], quercetin-3-*O*-neohesperidoside **2**, quercetin-3-*O*-*β*-galactoside **3** [19], isoquercitrin **4** [41], quercetin **5** [41], kaempferol 3-*O*-*β*-glucoside **6** [42], and scopoletin **7** [20].The structures of the isolated compounds were elucidated based on spectroscopic data, and by comparison with data reported in the literature (Supplementary Figures S1–S23).

### 3.2. In Vivo Investigation of the Antioxidant and Anti-Inflammatory Effects of the Isolated Compounds

The findings of the current study showed that the induction of T2DM in experimental rats by HFD + STZ (30 mg/kg) led to a significant increase in % body weight, adipose tissue index, liver index, liver enzymes, FBG, insulin resistance, TG, TC, LDL-C, and a significant decrease in insulin sensitivity and HDL-C. Ethyl acetate fraction of *C. acutum* excreted the best anti-hyperglycemic effect in rats with T2DM. Rats that received this fraction exhibited the lowest FBG, fasting plasma insulin, HOMA-IR, TG, LDL-C, and the highest HDL-C levels compared to the groups that received the crude extract and all other fractions. Ethyl acetate fraction administration also resulted in the lowest % increase in body weight, adipose tissue index, liver index and liver enzymes. The data of treatment with the crude extract, hexane, chloroform, ethyl acetate, and butanol fractions of *C. acutum* are represented in Supplementary Tables S1–S3.

Consequently, ethyl acetate fraction was subjected to further bio-activity guided isolation of five compounds—rutin (C1), quercetin 3-*O*-neohesperdoside (C2), quercetin-3-*O*-*β*-galactoside (C3), isoquercetin (C4), and quercetin (C5). The groups that received C1, C3, and C5 had significantly lower FBG, fasting plasma insulin and HOMA-IR compared to the T2DM control rats (*p* < 0.05). Only C3-administered rats exhibited high insulin sensitivity index (QUICKI) (Table 2). Moreover, levels of total cholesterol and LDL-C were significantly lowered in all treated groups compared to the HFD + STZ control group, but only the rats that were given C1, C2 and C3 had significantly lower TG, % increase in body weight, adipose tissue index, and high HDL-C levels compared to the control group (*p* < 0.05) (Table 3). The recorded food intake of the experimental rats was slightly higher in the HFD + STZ control group compared to the normal group, but with no significant difference. Overall, there was no significant effect of the investigated compounds on the food intake of the rats (Supplementary Table S4). Additionally, liver index was significantly lowered on treatment with C1, C3, and C5. All compounds except C4 lowered serum ALT significantly, but only C3 and C5 significantly decreased serum AST levels (*p* < 0.05). Serum levels of ALT and AST in both C3- and C5-treated groups were not significantly different compared to the normal rats (Table 4). The effect of the isolated flavonoids on the amounts of lipids in the liver was determined as well. All the investigated compounds decreased the levels of TC and TG in the liver, with the group treated with C3 showing the lowest TC and TG levels (Table 4).

**Figure 2:**
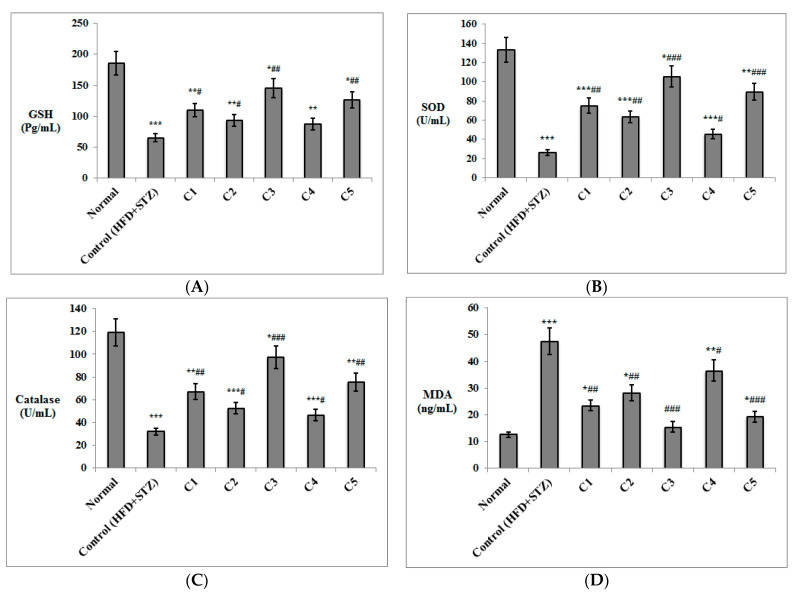

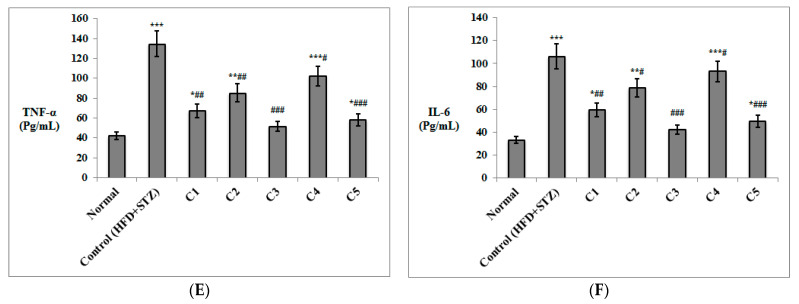
Antioxidant and anti-inflammatory effects of the isolated compounds on erythrocyte lysate levels of (**A**) GSH, (**B**) SOD enzyme, (**C**) catalase enzyme, as well as serum levels of (**D**) MDA, (**E**) TNF-α, and (**F**) IL-6. (*n* = 6–8). GSH = reduced glutathione; SOD = superoxide dismutase; MDA = malondialdehyde; TNF-α = tumor necrosis factor-alpha; IL-6 = interleukin-6. Levels of MDA, TNF-α and IL-6 were determined in serum, whereas concentrations of GSH, SOD and catalase were estimated in the erythrocyte lysate. C1: Rutin; C2: Quercetin 3-*O*-neohesperdoside; C3: Quercetin-3-*O*-*β*-galactoside; C4: Isoquercetin; C5: Quercetin. Data are expressed as mean ± SD and analyzed. Statistical significance in relation to the normal group was assigned at * *p* < 0.05; ** *p* < 0.01; and *** *p* < 0.001; whereas statistical significance in relation to the T2DM control group (high fat diet + Streptozotocin) was assigned at ^#^ *p* < 0.05; ^##^ *p* < 0.01; and ^###^ *p* < 0.001 using one-way ANOVA followed by Bonferroni’s post hoc test.

The antioxidant effect of the isolated compounds was investigated (Figure 2), where the T2DM control rats had significantly lower levels of GSH, SOD, and catalase in erythrocyte lysates and significantly higher serum levels of MDA (*p* < 0.001) compared to the normal rats. Both C3 and C5 resulted in increased levels of GSH (*p* < 0.01), SOD (*p* < 0.001), catalase (*p* < 0.001 for C3; *p* < 0.01 for C5), and decreased serum levels of MDA (*p* < 0.001) compared to the HDF + STZ control group. Similarly, C1 and C2 enhanced levels of GSH (*p* < 0.05), SOD (*p* < 0.01), and catalase (*p* < 0.01 for C1; *p* < 0.05 for C2) and reduced serum levels of MDA (*p* < 0.01) compared to the T2DM control group. Rats treated with C4 didn’t show a significant enhancement of GSH levels, but had significantly higher SOD and catalase values and decreased serum MDA (*p* < 0.05), compared to the control rats.

Serum levels of the inflammatory markers TNF-α and IL-6 were also significantly increased in the HFD + STZ control rats compared to the normal group (*p* < 0.001). Both markers decreased significantly in all treated group (*p* < 0.001 for C3 and C5; *p* < 0.01 for C1; *p* < 0.05 for C4). C2 significantly lowered both TNF-α and IL-6 (*p* < 0.01 and *p* < 0.05, respectively) compared to the HDF + STZ control group. Interestingly, levels of both markers in the rats treated with C3 were not significantly different compared to the normal group (Figure 2).

**Figure 3:**
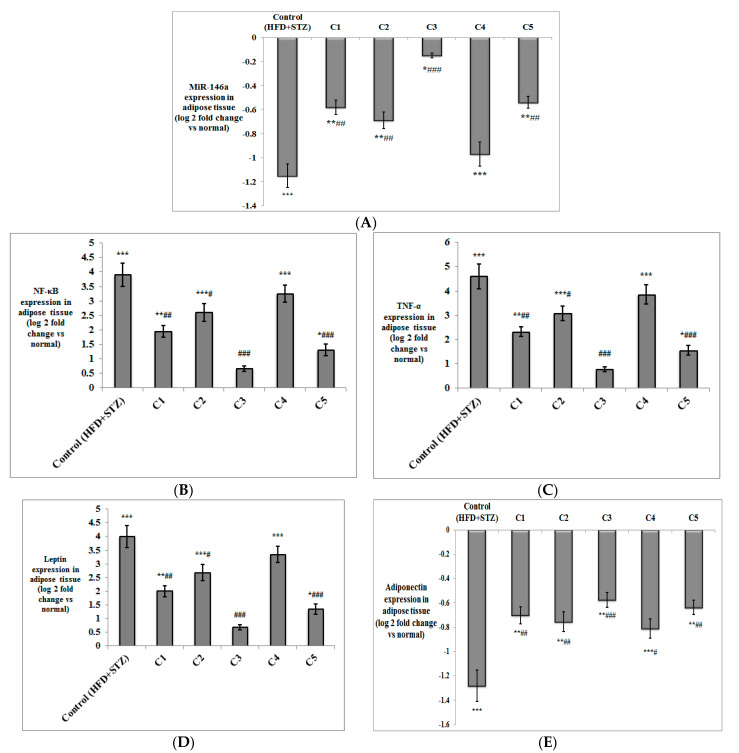
The effect of the isolated compounds on the expression of (**A**) miR-146a, (**B**) NF-κB, (**C**) TNF-α (**D**) leptin, and (**E**) adiponectin in the adipose tissue of the experimental rats. (*n* = 6–8). NF-κB = nuclear factor kappa B; TNF-α = tumor necrosis factor-alpha. C1: Rutin; C2: Quercetin 3-*O*-neohesperdoside; C3: Quercetin-3-*O*-*β*-galactoside; C4: Isoquercetin; C5: Quercetin. Data are expressed as mean ± SD of the log 2-fold change relative to the normal group (log 2 (1) = zero, i.e., base line). Statistical significance in relation to the normal group was assigned at * *p* < 0.05; ** *p* < 0.01; and *** *p* < 0.001; whereas statistical significance in relation to the T2DM control group (high fat diet + Streptozotocin) was assigned at ^#^ *p* < 0.05; ^##^ *p* < 0.01; and ^###^ *p* < 0.001 using one-way ANOVA, followed by Bonferroni’s post hoc test.

**Figure 4:**
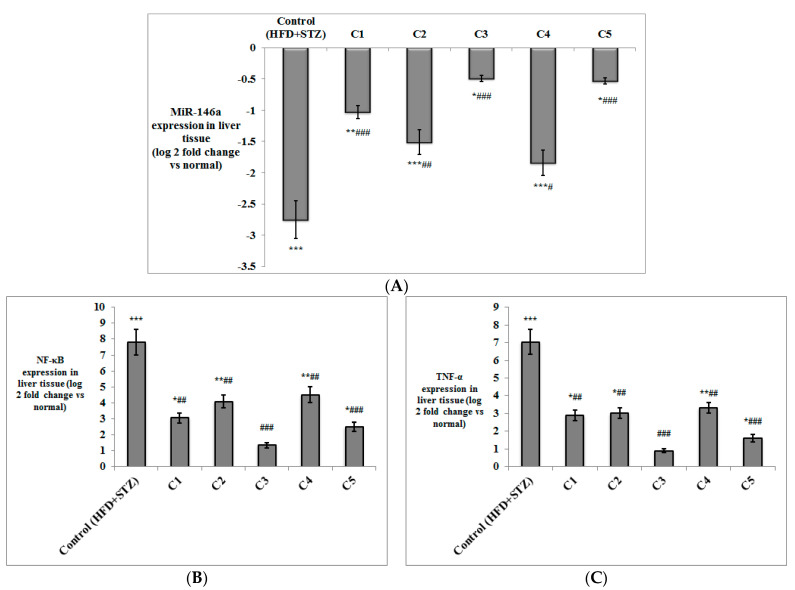
The effect of the isolated compounds on the expression of (**A**) miR-146a, (**B**) NF-κB, (**C**) TNF-α in the liver tissue of the experimental rats. (*n* = 6–8). NF-κB = nuclear factor kappa B; TNF-α = tumor necrosis factor-alpha. C1: Rutin; C2: Quercetin 3-*O*-neohesperdoside; C3: Quercetin-3-*O*-*β*-galactoside; C4: Isoquercetin; C5: Quercetin. Data are expressed as mean ± SD of the log 2 fold change relative to the normal group (log 2 (1) = zero, i.e., base line). Statistical significance in relation to the normal group was assigned at * *p* < 0.05; ** *p* < 0.01; and *** *p* < 0.001, whereas statistical significance in relation to the T2DM control group (high fat diet + Streptozotocin) was assigned at ^#^ *p* < 0.05; ^##^ *p* < 0.01; and ^###^ *p* < 0.001 using one-way ANOVA followed by Bonferroni’s post hoc test.

Besides the determination of TNF-α levels in serum, its expression was also evaluated in both the adipose and liver tissues, alongside NF-κB (Figure 3 and Figure 4). The expression of both genes was significantly up-regulated in both the liver and adipose tissue of the T2DM control rats (*p* < 0.001) compared to the normal group. NF-κB and TNF-α were significantly down-regulated on treatment with C1 (*p* < 0.01), C2 (*p* < 0.05 in adipose tissue; *p* < 0.01 in liver), C3 (*p* < 0.001) and C5 (*p* < 0.001) compared to the T2DM control group. The expression of NF-κB and TNF-α was down-regulated only in the liver of the group treated with C4 (*p* < 0.01), but not in the adipose tissue. It is worth noting that the expression of both inflammatory genes in the adipose tissue and liver of the group treated with C3 was not significantly different in comparison to the normal rats.

The expression of miRNA-146a was measured in both adipose and liver tissues of the experimental rats (Figure 3 and Figure 4), where its expression was significantly down-regulated in both tissues in the HFD + STZ control rats (*p* < 0.001) compared to the normal rats. MiRNA-146a expression was significantly up-regulated in the adipose tissue of the groups treated with C1, C2, C5 (*p* < 0.01), and C3 (*p* < 0.001) compared to the control group. In the liver tissue, the expression of miRNA-146a was also up-regulated in all treated groups compared to the T2DM control rats (*p* < 0.001 for C1, C3 and C5; *p* < 0.01 for C2; and *p* < 0.05 for C4).

The expression of the adipokines, leptin and adiponectin, was determined in the adipose tissue of the study groups (Figure 3). As expected, leptin was significantly up-regulated, while adiponectin was significantly down-regulated in the T2DM control rats (*p* < 0.001) compared to the normal rats. Expression of leptin was down-regulated in the groups treated with C1 (*p* < 0.01), C2 (*p* < 0.05), C3 and C5 (*p* < 0.001) compared to the control group. Up-regulation of adiponectin was observed in all treated groups (*p* < 0.001 for C3; *p* < 0.01 for C1, C2 and C5; *p* < 0.05 for C4) in comparison with the HFD + STZ control rats. 

**Figure 5:**
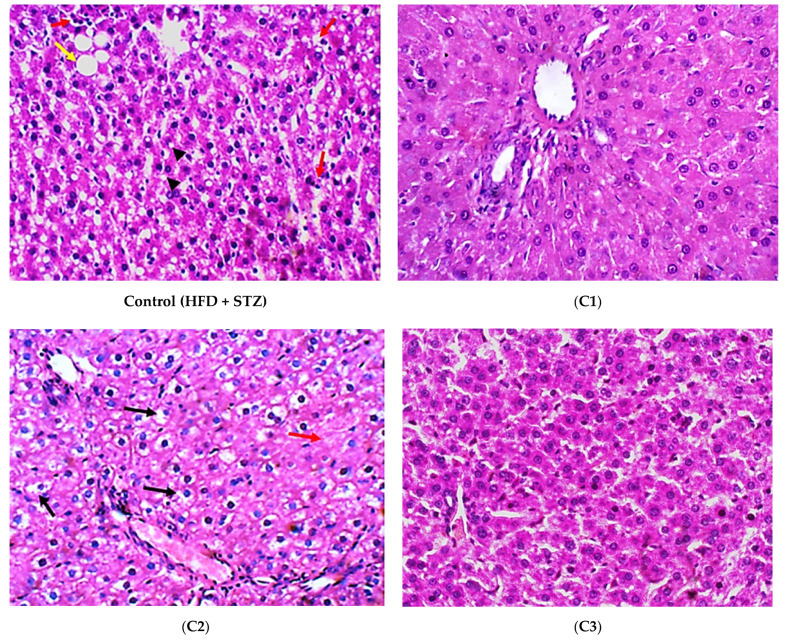

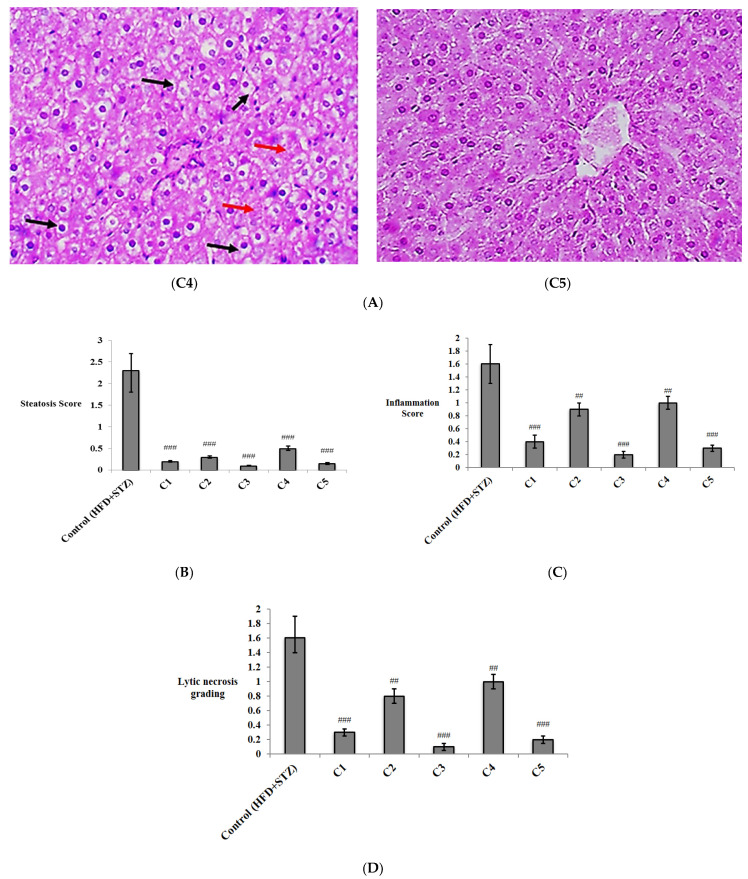
The histopathological study of hepatic tissues from different study groups. (**A**) Photomicrographs of hepatic tissues from different experimental groups stained by hematoxylin and eosin (H&E) and examined with magnifying power (40×). HFD + STZ control group showed hepatocytes with micro-vesicular steatosis (arrow heads), as well as macro-vesicular steatosis (yellow arrow). There were scattered foci of lytic necrosis of hepatocytes (red arrows) (H&E, 40×). (**C1**): Rutin-treated group showed uniform hepatocytes with no evidence of injury (H&E, 40×). (**C2**): Quercetin 3-O-neohesperdoside-treated group: Hepatocytes showed no evidence of steatosis, yet, several hepatocytes still showed hydropic degeneration (black arrows), with few foci of lytic necrosis (red arrows) (H&E, 40×), (**C3**): Quercetin-3-*O*-*β*-galactoside-treated group, in which hepatocytes showed no evidence of steatosis or hydropic degeneration. No other pathological changes observed (H&E, 40×), (**C4**): Isoquercetrin-treated group; in which hepatocytes showed no evidence of steatosis, yet, several hepatocytes still show hydropic degeneration (black arrows), with few loci of lytic necrosis (red arrows) (H&E, 40×). (**C5**): Quercetin-treated group; in which hepatocytes showed no evidence of steatosis or hydropic degeneration. No other pathological changes observed (H&E, 40×), (**B**–**D**) histopathological scores of liver steatosis, inflammation, and lytic necrosis, respectively. Data are expressed as mean ± SD and analyzed using one-way ANOVA followed by Bonferroni’s post hoc test. ^##^ significantly different compared to the T2DM control group (high fat diet + Streptozotocin) at at *p* < 0.01; ^###^ at *p* < 0.001.

The histopathological evaluation was performed in all experimental groups. Hepatocytes in the T2DM control group showed both macro- and micro-vesicular steatosis along with the scattered foci of lytic necrosis of hepatocytes. Hepatocytes in the C1-treated group showed uniform hepatocytes, with no evidence of injury or hepatic steatosis. Hepatocytes in C2 group showed no evidence of steatosis; yet, several hepatocytes still showed hydropic degeneration and focal lytic necrosis. In the group treated with C3, hepatocytes showed no evidence of steatosis or hydropic degeneration. No other pathological changes were observed. The C4-treated group showed no evidence of steatosis in hepatocytes; yet, several hepatocytes still showed hydropic degeneration and focal lytic necrosis. Hepatocytes of the group treated with C5 showed no evidence of steatosis or hydropic degeneration. No other pathological changes were observed (Figure 5A). Histopathological grading of the hepatic lesions indicated that steatosis was significantly decreased in all treated groups (*p* < 0.001) (Figure 5B). Inflammation and lytic necrosis were also significantly decreased in the groups treated with C2 and C4 (*p* < 0.01), and in the groups treated with C1, C3, and C5 (*p* < 0.001) (Figure 5C,D). The lytic necrosis observed in the histological photos is often caused by the ballooning degeneration of hepatocytes [43,44]. Ballooning degeneration is a form of liver cell degeneration that is associated with cell swelling and enlargement, and represents a hallmark of steatohepatitis [45]. Fibrosis was not markedly determined in the liver tissue of any of the examined groups (Figure 5A).

**Figure 6:**
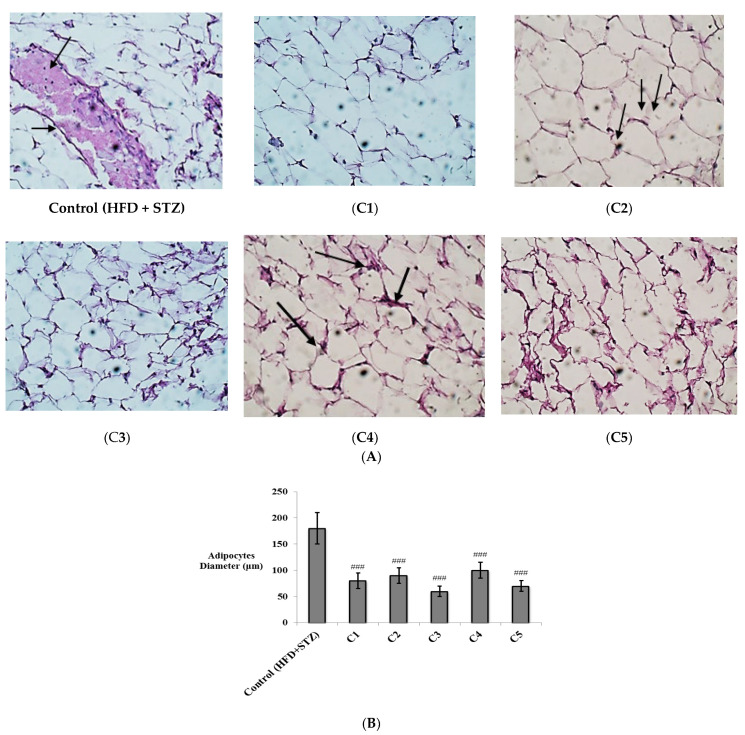
The histopathological study of adipose tissues from different study groups. (**A**) Photomicrographs of adipose tissues from different experimental groups stained by hematoxylin and eosin (H&E) and examined with magnifying power (40×). HFD + STZ control group; adipose tissue showed many congested vessels (Black arrows) (H&E, 40×). (**C1**): Rutin-treated group; adipose tissue showed uniform fat cells, with non-congested blood vessels (H&E, 40×). (**C2**): Quercetin 3-*O*-neohesperdoside-treated group; adipose tissue showed few congested vessels (Black arrows). (**C3**): Quercetin-3-*O*-*β*-galactoside-treated group; adipose tissue showed uniform fat cells, with non-congested blood vessels. No inflammatory changes observed (H&E, 40×), (**C4**): Isoquercetrin-treated group; adipose tissue showed few congested vessels (Black arrows) (H&E, 40×), (**C5**): Quercetin-treated group; adipose tissue showed uniform fat cells, with non-congested blood vessels (H&E, 40×). No inflammatory changes observed. (**B**) Diameter of adipocytes (µm) expressed as mean ± SD and analyzed using one-way ANOVA followed by Bonferroni’s post hoc test. ^###^ significantly different compared to at *p* < 0.001.

Additionally, histopathological examination of the adipose tissue showed many congested blood vessels in the HFD + STZ control group. The congested vessels were decreased in the groups treated with C2 and C4, and almost diminished in the groups treated with C1, C3, and C5, which showed uniform fat cells (Figure 6A). Determination of the adipocyte diameter revealed a significant decrease in all the treated groups (*p* < 0.001) (Figure 6B).

In general, the results of histopathological examination were confirmatory of the biochemical measures, and proved the potent anti-inflammatory effects of the investigated compounds in both the liver and adipose tissue, especially C3 (quercetin-3-*O*-*β*-galactoside) and C5 (quercetin).

Obesity, diabetes, oxidative stress, and inflammation are inter-connected, where chronic inflammation is associated with metabolic syndrome, and is one of the crucial risk factors of developing T2DM [46]. Hyperglycemia and insulin resistance generate reactive oxygen species (ROS) and oxidative stress, which in turn play a pivotal role in the development of T2DM complications [47]. Biochemical alterations involve increased lipid peroxidation; indicated by increased MDA levels, impaired glutathione metabolism and decreased levels of the antioxidant enzymes SOD and catalase [48]. Oxidative stress stimulates the production of inflammatory mediators and inflammation, and in turn, enhances the generation of ROS [46]. Obesity is one of the most important underlying causes of the state of chronic inflammation [49], in which the adipocytes produce various proinflammatory cytokines, including TNF-α and IL-6, which are involved in in the activation of the NF-κB pathway [50]. Alterations in the expression of adipokines are also associated with obesity-induced inflammation, where the levels of circulating leptin and leptin expression in the adipose tissue increase [51]. Inflammation mediates leptin resistance [52], which in turn leads to the development of metabolic disorders [53], and was positively correlated with insulin resistance [54]. Conversely, expression and levels of adiponectin are lowered in obesity. Adiponectin increases insulin sensitivity in muscle and liver, and is also known for its antioxidant and anti-inflammatory activities [55].

T2DM and liver dysfunction are consistently linked, and insulin resistance is one of the major causes of nonalcoholic fatty liver disease (NAFLD) [56]. Diabetes triggers oxidative stress in the liver and reduces its antioxidant defenses [57,58]. The proinflammatory cytokine TNF-α plays a major role in NAFLD development through upregulating key molecules associated with fibrosis in the liver [59].

The role of NF-κB as a central regulator of inflammation has been extensively studied. NF-κB is involved in all the inflammatory processes that are linked to the induction of obesity-related metabolic disease. NF-κB signaling is upregulated by excess nutrients, metabolic stress, and proinflammatory cytokines, and its activation promotes the proinflammatory state linked with obesity and leads to development of insulin resistance [60]. NF-κB is a key M1 macrophages transcription factor and acts as an inducer of various inflammatory genes, including TNF-α, IL-6, and IL-1β [61]. NF-κBis also crucialto theregulation of T-cell differentiation. The NF-κB signaling pathway represents a promising target for anti-inflammatory remedies [62]. It was suggested that blocking NF-κB-driven inflammation can alleviate hyperglycemia and insulin resistance in T2DM [63].

MiRNAs are small non-coding RNA molecules that play an essential role in the regulation of cellular gene expression; either by the suppression of protein translation or by induction of degradation of the target mRNA [64]. miRNA-146a, encoded on human chromosome 5q33.3, was identified as a negative feedback regulator of inflammation in human adiopocytes through the downregulation of the pro-inflammatory interleukin 1 receptor associated kinase 1 (IRAK1) and tumor necrosis factor receptor associated factor 6 (TRAF6) [65]; both targets are upstream of the NF-κB signaling pathway, and their regulation inhibits the expression of NF-κB target genes, such as TNF-α, IL-1β, IL-6, and IL-8 [66]. miR-146a was also reported to repress inflammation and diet-induced obesity and regulate metabolic processes in experimental mice [67]. miR-146a mediates its anti-inflammatory effects in a variety of immune cells by repressing the signaling of NF-kB and Toll-like receptor 4 (TLR4) [68]. On the other hand, decreased serum level of miR-146a was considered as a biomarker of chronic inflammation in T2DM patients [69], and its deficiency significantly increased obesity-induced inflammation in the liver of obese mice [70]. Moreover, it was suggested that the deficiency of miR-146a may contribute to the severe state of COVID-19 observed in diabetic, obese and hypertensive patients through enhanced inflammation and fibrosis [71]. Interestingly, miR-146a was suggested as an inflammatory regulator and a potential therapeutic molecule for obesity-induced diabetes and related periodontal diseases [72]. The relation of miR-146a with oxidative stress was also proven; where miR-146a was considered as a potential regulator of oxidative stress and inflammation in the brain of chronic T2DM rats through regulating the NF-κB pathway [73]. The miR-146a promoter analysis provided evidence that its induction is NF-κB -dependent [74]. This suggests a negative regulatory loop, in which NF-κB upregulates miR-146a; which, upon processing and maturation, down-regulates IRAK1 and TRAF6 to inhibit the activity of NF-κB [75].

The findings of the currents study are consistent with previous reports, where quercetin (C5) was suggested to regulate obesity through the inhibition of lipid accumulation and reducing body weight in a mouse model of obesity [76]. Quercetin was reported to lower blood glucose via the stimulation of glucose uptake through a mitogen-activated protein kinase (MAPK) insulin-dependent mechanism [77]. In the animal model of T2DM, quercetin decreased plasma TC and TG, and increased plasma HDL-C and adiponectin [78]. Quercetin also decreased plasma levels of TNF-α and leptin, suppressed the expression of NF-κB, and increased antioxidant enzyme levels in diet-induced obese mice [79].

Similarly, isoquercetin (C4) improved glucose tolerance in a rat model of T2DM, reduced serum ALT and AST, and increased levels of HDL-C, SOD, and GSH [24]. Isoquercetin also significantly decreased body weight, attenuated liver damage, decreased leptin, and increased adiponectin in the HFD mouse model [80]. Recently, isoquercetin was reported to play a neuroprotective effect in rats exposed to ischemic stroke through inhibiting NF-κB and suppressing the inflammatory response [81].

Rutin (C1) is known to have potent antioxidant effects, particularly in neurodegenerative disorders [82]. It was found to protect against neurotoxicity through reducing lipid peroxidation and enhancement of the levels of GSH, SOD and catalase [83]. In STZ induced diabetes in rats, rutin significantly lowered blood glucose levels and reduced the expression of TNF-α [84]. Additionally, rutin had an anti-inflammatory effect against lipopolysaccharide-induced mastitis through attenuating endoplasmic reticulum stress and inhibition of the NF-κB pathway [85]. Potent anti-oxidative properties of quercetin-3-*O*-*β*-galactoside (C3) were also reported in an in vitro study [86].

The anti-inflammatory effects of flavonoids were substantially attributed to their ability to target the signaling pathway of NF-κB [87]. Quercetin was reported to decrease the transcriptional activity of NF-kB in stable coronary artery disease [88], and to lower the NF-kB p65 subunit DNA binding activity through dephosphorylation and up-regulation of the inhibitor of kappa B α (IkB-α) in human colon cancer cells [89]. Isoquercetin was also suggested to ameliorate cisplatin induced nephrotoxicity through the downregulation of the expression of p65 and phosphorylated p65 [90]. Moreover, the protective activity of rutin against cardiotoxicity in rats was mediated through upregulating IκB-α and downregulating NF-κB expression [91].

Interestingly, in vitro studies showed that quercetin upregulated the expression of miR-146a in human breast cancer cell lines [92]. In rheumatoid arthritis, quercetin treatment was shown to elevate the level of miR-146a to inhibit the migration and invasion of fibroblast-like synoviocytes (FLSs) [93]. However, the current study is one of the first research articles to address the effect of other isolated flavonoids on the expression of miR-146a in vivo, and to link this relation to their antioxidant and anti-inflammatory properties.

### 3.3. Molecular Modelling Investigation

For gaining more insights regarding the molecular aspects and differential anti-inflammatory activities of the examined compounds, molecular docking outflow was conducted on different biomolecular targets involved within the NF-κB signaling pathway. The in silico study was conducted at the DNA-binding segments of one of the main NF-κB dimers, RelB:p52 (PDB: 3DO7) heterodimer, in order to assess the compound’s potentiality to hamper the NF-κB-specific DNA gene binding [37]. The latter approach would provide reliable bases correlating with the compound’s in vitro anti-inflammatory activity to the obtained downregulated expressions of several NF-κB-directed pro-inflammatory mediators, including TNF-α and IL-6 [62]. Compared to other NF-κB dimers, the RelB:p52 constellation is capable of recognizing a broad spectrum of κB sites, causing this particular heterodimer to be associated with the DNA-specific genes in a non-discriminatory fashion [37]. Thus, the RelB:p52 heterodimer has been considered as an attractive therapeutic target for hampering the NF-κB signaling pathway. The molecular modelling investigation further evaluated the compound’s potentiality to accommodate the ATP-substrate binding pocket of the human inhibitory-κB kinase (hIKK; PDB: 4KIK) [38]. This protein target represents a key regulator of NF-κB as being responsible for initial phosphorylation and successive ubiquitination for the final degradation of IκB proteins permitting NF-κB dimer liberation for subsequent nuclear translocation and specific DNA gene activation [94]. Setting theses biological targets for the in silico study is highly rationalized, since several flavonoids and polyphenolic compounds exhibited relevant modulation of the NF-κB pathway, affecting hIKK activity, IκB degradation, as well as NF-κB nuclear translocation, and binding to specific DNA genes.

Quercetin, the plant flavanol, showed the downregulation of the expression of NF-κB-associated genes within the murine RAW264.7 macrophages, which was correlated to the blocked nuclear translocation of RelA and p50 subunits [95]. This was further conformed through both in vitro and in vivo studies, where quercetin inhibited the IκB phosphorylation within macrophages, human mast cells, and the dextran sulfate sodium rodent colitis model [96,97]. In addition to blocking the hIKK activation, quercetin reduced the NF-κB-specific DNA gene association within inflammatory primed mice microglia cells-treated with lipopolysaccharides or interferon-γ [98]. Similar findings were obtained for luteolin, galangin, and genistein, where hIKK /NF-κB inhibition and pro-inflammatory mediator down regulation were depicted in co-cultured mice RAW 264.7 macrophages/intestinal epithelial Caco-2 and/or lipopolysaccharide-induced dendritic/epithelial cells [99,100,101]. Both epigallocatechin-3-gallate and catechin showed inhibitory activity against hIKK, IκB degradation, and RelA NF-κB subunit nuclear translocation, as well as reducing NF-κB-DNA binding within murine peritoneal macrophages or phorbolmyristate acetate-stimulated Jurkat T-cells, respectively [102,103,104,105,106]. Other polyphenols, including hydroxytyrosol and resveratrol, showed the inhibition of NF-κB activity within lipopolysaccharide-triggered human umbilical vein endothelial cells [107].

The crystalline NF-κB heterodimer (PDB: 3DO7) is composed of RelB and p52 subunits, being associated through a 10-residue-long linker of their respective immunoglobulin-like domains at the C-terminals (Supplementary Figure S24A). The interaction between the NF-κB and DNA occurs within a solvent-accessible surface area (~3200 Å^2^) formed by the N-terminal and smaller C-terminal domains, mediating several base-specific and non-specific contacts, respectively. The DNA-binding segment of each subunit is highly homologous, where Arg117, Arg119, Glu123 from RelB and the corresponding Arg25, Arg54, Glu58 from p52 (hereafter, p52 residues are denoted italics) are involved in base-specific polar interactions with the flanking CCC:GGG core sequences (Supplementary Figure S24B). Polar contacts are also mediated by Arg125, as well as Ser61, His62, and Lys221 residues, which are missing from their corresponding residues on respective homologous subunits. Non-polar base-specific interactions are also considered, where Tyr120 and its corresponding Tyr55 mediate relevant sequence-specific van der Waals contacts with the hydrophobic carbons and rings of the DNA bases, including T+2 and T–1:C–2, respectively. Mutagenesis studies emphasized the crucial role of the above DNA base-recognizing residues, since mutant proteins showed lower affinity towards several NF-κB-specific DNA genes, compared to the wild-type heterodimer. Based on the above evidence, a ligand which is capable of interacting with these important residues is considered efficient to block the NF-κB recognition of κB DNA sites.

**Figure 7:**
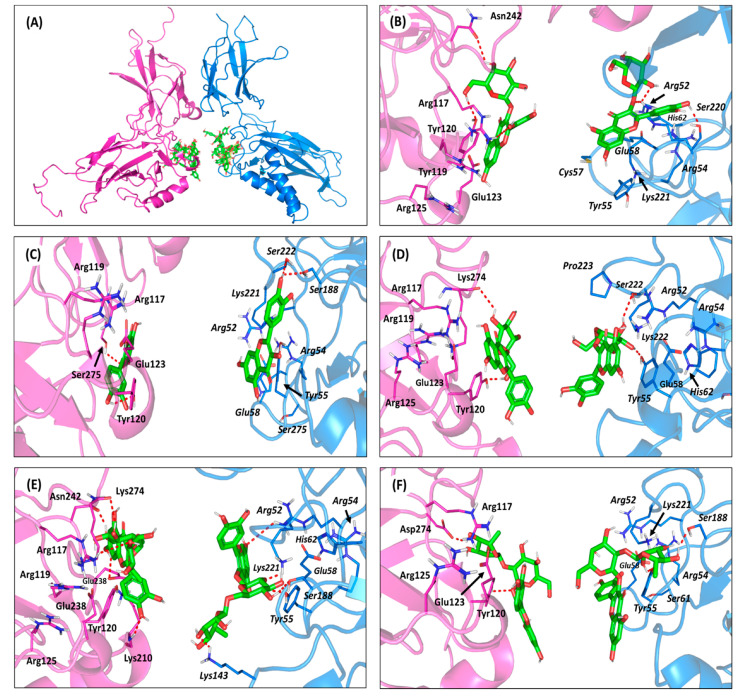
Molecular docking investigation of the isolated compounds at DNA-binding domains of the NF-κB RelB:p52 heterodimer (PDB ID: 3DO7). (**A**) An overlay of the docked ligands (green sticks) at both NF-κB heterodimer subunits, RelB (magenta cartoon) and p52 (marine blue cartoon). (**B**–**F**) The predicted binding modes of quercetin-3-*O*-*β*-galactoside(**B**); quercetin (**C**); isoquercitrin(**D**); rutin(**E**); and quercetin-3-*O*-neohesperdoside (**F**) at RelB (left side) and p52 (right side) illustrated as cartoon 3D-representation within their respective magenta and marine blue colors. Polar interactions, represented as hydrogen bonds, are illustrated as red dashed lines and only residues (lines), located within a 4 Å radius of bound ligand, are displayed and labeled with sequence number.

Molecular docking simulation of the examined isolated compounds on the RelB:p52:DNAheterocomplex illustrated significant ligand accommodation at the NF-κB-specific DNA binding domains of each protein subunit (Figure 7A). Significant polar and hydrophobic interactions between were illustrated for the docked ligands and the base-specific residues at the NF-κB subunits. As a general observation, ligands bounded to the RelB subunits showed higher docking scores (−4.90 ± 0.34 up to −7.47 ± 0.22 Kcal/mol), compared to those at p52 (−4.12 ± 0.14 up to −6.86 ± 0.66 Kcal/mol), suggesting a predicted higher affinity towards the earlier NF-κB heterodimer subunit.

Moreover, ligands with higher glycosidic moieties, such as rutin and quercetin-3-*O*-neohesperdoside, were assigned with significant negative docking scores (average -6.40 ± 0.38 Kcal/mol). Such observation implies the predicted significant role of these highly polar sugar groups for anchoring their respective flavonoid-based scaffolds at the NF-κB-specific DNA binding domains. Nevertheless, the non-glycosidic quercetin showed higher docking score relative to its sugar-3-*O*-linked analogue, isoquercitrin (−5.96 ± 0.51 versus −4.90 ± 0.34 Kcal/mol), which was similar for the single glycosylated quercetin-3-*O*-*β*-galactoside relative to its double glycosylation at quercetin-3-neohesperidoside (−6.98 ± 0.22 versus −6.32 ± 0.89 Kcal/mol). The latter differential findings would highlight the significant role of the specific type of sugar part, rather than its number or presence for guiding the ligand’s binding at the target site. Explaining the ligand-specific docking and target binding was processed through intensive ligand-target interaction analysis of the docking poses.

Quercetin-3-*O*-*β*-galactoside predicted one of the highest docking scores at the RelB subunit (−6.98 ± 0.22 Kcal/mol) through furnishing strong hydrophilic contacts with several base-specific residues (Figure 7B). The β-hydroxyl groups at C4 and C6 of the galactosyl moiety predicted double hydrogen bonding with the oxygen atoms of Tyr120 and Asn242 sidechains (Supplementary Table S5). Additionally, the meta-hydroxyl group of the ligand’s aromatic ring substitution depicted significant polar contact with the guanidine sidechain of one of the significant κB base-recognizing residues, Arg117. The latter favored polar contacts brought the ligand’s coumarin aromatic ring at proximity and optimum orientation towards the Tyr120 sidechain mediating relevant ring π-π hydrophobic interactions (3.6 ± 0.87 Å). Comparable docking pose was predicted for quercetin-3-*O*-*β*-galactoside at p52 subunit, where relevant polar contacts with corresponding polar residues, Arg52 and Ser220, were compared, with its sugar and terminal hydroxylated aromatic ring substitution, respectively. However, the weaker ring π–π hydrophobic interactions with the relatively more distant hydrophobic residue, Tyr55 (5.2 ± 0.78 Å), could reason for the ligand’s lower docking score (−6.06 ± 0.06 Kcal/mol) at the p52 homologous subunit.

Comparing quercetin-3-*O*-*β*-galactoside with its non-glycosylated form illustrated the significant impact of the sugar moiety for ligand-target binding. Lacking the sugar group, the quercetin flavanol showed more intact anchoring at the DNA-binding domains surface. At RelB subunit, the ligand has close polar contact with Ser275 and Tyr120 (Figure 7C). The stability of the ligand at target site was further mediated via the phenyl substitution, showing favored proximity with Tyr120 sidechain (4.5 ± 0.51 Å). Notably, the depicted π-π interaction was at the α-face of Tyr120, rather than at its β-face, where the latter was the case for the quercetin-3-*O*-*β*-galactoside binding mode. The same ligand-target tightness was depicted at p52, permitting strong hydrogen bond interactions with Ser188 and Ser222, besides a favored orientation towards the Tyr55 aromatic ring (3.0 ± 0.72 Å). Thus, the absence of sugar moiety may be reasoned for no polar contacts with κB base-recognition arginines, as compared to quercetin-3-*O*-*β*-galactoside; the thing that was obvious is lower docking scores and correlated biological activity. However, lacking the sugar moiety allowed the tight binding of the ligand at the DNA-binding interface, allowing significant compensation, keeping the docking score at relatively moderate values.

Regarding the quercetin-3-*O*-*β*-galactoside closest analogue, isoquercitrin showed altered ligand orientation at the protein target site. Unlike quercetin-3-*O*-*β*-galactoside, the α-isomeric hydroxyl group at C4 of isoquercitrin sugar part lacked any polar contacts with relevant RelB residues. This was recognized for the unfavored α-face orientation of OH, being directed towards the solvent side rather than the protein interface (Figure 7C). As such, the ligand predicted hydrogen bond pair with the carbonyl oxygen of Lys274 mainchain, causing a significant retraction of the flavanol central scaffold to be far from the key κB base-recognizing residues, Arg117 and Arg119. Despite the extra polar interaction of the sugar moiety with the Tyr120 hydroxyl group, the depicted ligand’s orientation was unfavored for the optimum proximity of the ligand’s aromatic ring towards the RelB Tyr120 sidechain (6.7 ± 0.50 Å). Similarly, the binding mode of isoquercitrin at p52 showed the distant orientation of the coumarin scaffold from the corresponding hydrophobic residue Tyr55 sidechain (11.8 ± 0.43 Å). Such distant orientation was also driven by the steric hindrance imposed by the residues of the linker loop, particularly Pro223, against the ligand’s sugar moiety. All of these have served in furnishing poor docking scores for isoquercitrin (−4.90 ± 0.34 Kcal/mol at RelB and −4.12 ± 0.14 Kcal/mol at p52), which were not compensated by any polar interactions between the sugar moieties, and either Lys274 (Ser222) or Tyr120 (Tyr55) sidechains. The latter in silico findings would propose reasonable explanations for the superior reduction of pro-inflammatory gene expressions by quercetin-3-*O*-*β*-galactoside over isoquercitrin.

Moving towards the highly glycosylated ligands, favored ligand/target interactions were depicted at both RelB and p52 subunits. Owing to the planar structure of its double glycosylation (α-L-rhamnopyranosyl-(1→6)-*β*-D-glucopyranose), rutin depicted extended anchoring over a large surface of both the RelB and p52 DNA-binding domains (Figure 7D). Such extended binding modes permitted several favored polar contacts, with target residues, including the specific base-recognition ones. Rutin predicted significant hydrogen bonding with the RelB residues, including the sidechains of Arg117, Glu238 and Asn242, in addition to the mainchains of Lys210 and Lys274. Despite the lack of favored hydrophobic contacts, with the important base-recognition hydrophobic residue Tyr120 (8.6 ± 0.99 Å); rutin managed to furnish the highest depicted docking score (−7.47 ± 0.22 Kcal/mol) guided by its extensive polar network. On the other side, the ligand exhibited several polar contacts at p52 with Arg52, Tyr55, Arg221, Lys143, and Ser188. Moreover, the ligand’s chromone ring was anchored at relatively close orientation towards the corresponding Tyr55 (5.0 ± 1.03 Å), favoring relevant hydrophobic contacts, correlating with a nearly comparable docking score of −7.01 ± 0.66 Kcal/mol. Regarding the other double glycosylated ligand, quercetin-3-neohesperidoside exhibited non-linear conformation at both DNA binding domains of the RelB:p52 protein target. Having a different double glycosylation pattern of α-L-rhamnopyranosyl-(1→2)-α-D-galactopyranose, the ligand showed distinct anchoring at target site with its galactose sugar part directed towards the solvent side (Figure 7E). Such unfavored orientation of the central sugar linker caused a loss of significant polar contacts, with the target surface furnishing fewer hydrogen bonds relative to those for rutin. It is worth mentioning that quercetin-3-neohesperidoside was further stabilized at target site, through significant hydrophobic interactions with Tyr120 and its homologous residue Tyr55 at RelB and p52, respectively (~3.5 ± 0.45 Å). All the above binding preferentiality could be translated into quercetin-3-neohesperidoside, with lower docking scores coming second to both rutin and quercetin-3-*O*-*β*-galactoside (Supplementary Table S5). Additionally, the latter docking results would provide reasonable bases for quercetin-3-neohesperidoside lower in vitro anti-inflammatory activity concerning IL-6 and TNF-α expressions. Nevertheless, the molecular docking simulation data were not in good concordance with the quercetin-3-neohesperidoside and rutin, with lower biological activity than the non-glycosylated quercetin. Thus, it was suggested that the isolated compounds could also act on different NF-B pathway targets, including the literature-reported hIKK enzyme, leading to a collective biological profile.

In these regards, it was fundamental to further investigate the isolated compounds for the subsequent molecular docking study on this particular enzyme target.

The adopted crystalline hIKK (PDB: 4KIK) exhibits a shear-like trimodular structural arrangement of three distinct domains; (i) the N-terminal kinase domain, (ii) ubiquitin-like domain, and (iii) elongated α-helical dimerization/scaffold domain at the C-terminal (Supplementary Figure S25A) [38]. The combined N-terminal kinase and ubiquitin-like domain resembles the shear handle, while the C-terminal dimerization/scaffold domain is the blade of the hIKK shear structure. This blade-like elongated structure (residues 410–664) consists of six α-helices interconnected through short β-loops, which is subdivided into the leucine zipper (458–479), helix-loop-helix domain (603–642), and C-terminal domain (637–642) designated for the binding of NF-κB essential modulator (NEMO; hIKKregulatory subunit). Both the ubiquitin-like and dimerization/scaffold domains provide the platform for NF-κB inhibitory proteins binding, and bringing them in proper orientation towards the N-terminal kinase domain for their phosphorylation.

The catalytic kinase domain is conserved among all kinases showing the typical bislobal kinase protein folding comprising the N-lobe and C-lobe, featuring the stranded β-sheets and α-helices secondary protein structures, respectively. The C-lobe possesses non-structured flexible segment “activation loop” (20–30 residues), where its N-terminal region shifts from disordered to ordered upon activation, while its C-terminal one forms a pocket that binds to the substrate protein residues (Supplementary Figure S25B). Both kinase lobes are connected through a hinge region forming the catalytic ATP-binding site, including the adenine-, sugar-, and tri-phosphate-specific pockets, in addition to the less conserved hydrophobic pocket and channel settled at the opposite sides of the adenine-specific pocket [38]. The hydrophobic pocket is considered a more buried cleft guided by a gatekeeper residue, where significant mutation of such residue with bulkier ones could mediate significant resistance against the binding of several of kinase inhibitors [108]. Generally, ATP does not accommodate these hydrophobic pockets, making such non-polar clefts relevant for superior binding of kinase inhibitors, as well as relative selectivity against particular kinase enzymes [109].

The crystalline ligand, KSA, accommodates the ATP-binding cleft forming strong polar interactions with mainchains of Glu97 and Cys99 at the hinge region, mediated by the ligand’s pyrrolidinone ring (Supplementary Figure S25C). The ligand’s bicyclic 10-memebered ring is oriented at the sugar-specific region, while furnishing a single polar interaction between its hydroxyl substitution and Glu149 lining residues. Further stabilization of KSA within the catalytic site and profound competition against ATP binding were illustrated through anchoring its aromatic rings towards the opposite-sided hydroponic pocket and channel, furnishing relevant hydrophobic contacts (π-H interactions) with Leu21 and Val29. Based on the above binding modes, the ability of any of the investigated compounds to hamper the hIKK catalytic transphosphorylation activity would be related to its comparable or even better binding pose at the ATP-binding cleft [110].

The pharmacophoric features of most of the investigated compounds illustrated significant resemblance with those of the ATP, suggesting a significant competitive binding approach for these compounds. The aromatic chromone ring with its polar oxygen functionality as well as dihydroxy aromatic ring substitution showed comparable pharmacophoric features relative to the adenine scaffold of the ATP molecule. Possessing several hydrogen bond donors and acceptors at the chromone nucleus corresponds to the significant capability of such a scaffold to mediate polar interactions with the relevant residues at the pocket’s hinge region. Moreover, the hydroxylated phenyl substitution within the investigated compound structures resembles an aromatic pharmacophoric feature capable of anchoring at either of the hydrophobic sites at the ATP-binding pocket. Interestingly, both the chromone and hydroxylated phenyl ring share similar pharmacophoric features; the thing that could allow equal opportunity for each of these two scaffolds to bind at the hinge region or at one of the hydrophobic sites. Notably, favoring one binding mode over the other could be highly driven by the preferential anchoring of the glycosidic moieties at the ATP-binding site. Possessing sugar moiety by almost all the investigated compounds allowed further resemblance with the ATP molecule for ribose ring, which would permit significant competitive binding at the target site. Different types of the glycosidic moieties would permit variable orientation near the sugar-specific pocket, which in turn, are translated as variable positioning of the ligand’s aromatic and polar pharmacophoric features within the kinase domain. Thus, differential binding modes would be expected for the investigated compounds, since they possess single, multiple, or even no glycosylated moieties. Such predicted differential binding modes would be thoroughly discussed within the forthcoming context.

**Figure 8:**
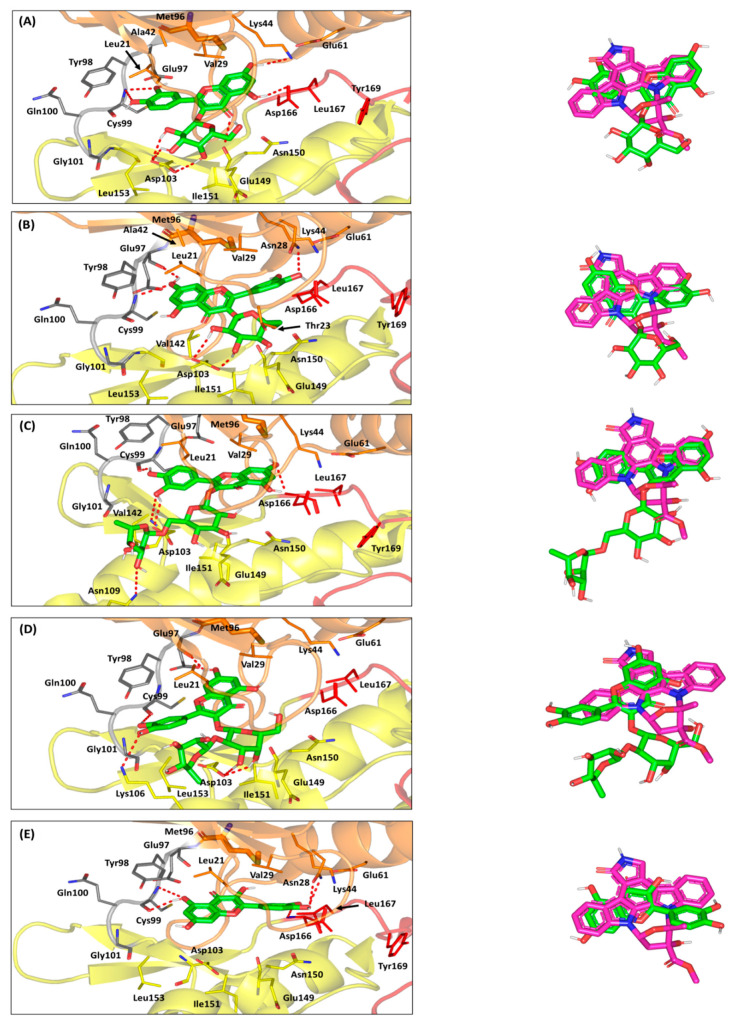
Molecular docking investigation of the isolated compounds at ATP-binding site of the hIKK protein target (PDB ID: 4KIK). Left panels illustrate the proposed docking poses for the investigated compounds (green sticks) at the catalyric ATP-binding site of IKK target protein (orange, yellow, gray, and red cartoons for N-lobe, C-lobe, hinge region, and activation segment, respectively); (**A**) quercetin-3-*O*-*β*-galactoside; (**B**) isoquercitrin; (**C**) rutin; (**D**) quercetin-3-*O*-neohesperdoside; (**E**) quercetin. On the right, overlay of docked isolated compounds (green sticks) and crystallized ligand, KSA (magenta sticks), depicting their comparative orientations within the catalytic binding site. Polar interactions, represented as hydrogen bonds, are illustrated as red dashed-lines and only residues (lines), located within 4 Å radius of bound ligand, are displayed, labeled with sequence number, and colored based on their respective position.

Molecular docking simulation revealed favorable predicted binding mode of quercetin-3-*O*-*β*-galactoside, showing great superimposition with the crystalline ligand conformation (root-mean square deviation; RMSD = 1.15 ± 0.34 Å) (Figure 8A). Optimum orientation was depicted for the ligand’s hydroxylated phenyl substitution towards the hinge region. Strong polar interactions are depicted between the ligand’s oxygen atom and NH mainchain of Cys99 residue (Supplementary Table S6). Moreover, the close proximity of the aromatic ring towards the pocket’s hydrophobic residues allowed significant hydrophobic contact (π-H interaction) with Leu21 (4.6 ± 0.43 Å). On the other hand, the ligand’s chromone ring is docked at the buried hydrophobic site, being at relevant distance far from any significant steric clashes with the Met96 gatekeeper residue. Further stabilization of the ligand’s central core was mediated through extended hydrogen bond network with Lys44, Glu149, and Asp166. The glycosidic moiety of the quercetin-3-*O*-*β*-galactoside depicted relevant orientation at the sugar-specific pocket furnishing triple hydrogen bonding with the Asp103 sidechain. Depicting more polar interactions with key pocket residues other than KSA would provide reason for the higher docking score of the docked ligand, as compared to the crystalline one (−7.914 ± 0.52 Kcal/mol versus −6.93 ± 0.79 Kcal/mol).

Concerning the quercetin-3-*O*-*β*-galactoside closest analogue, isoquercitrin exhibited altered orientation of the sugar moiety, owing to its α-isomeric hydroxyl group at the C4 position (Figure 8B). Alpha-face flipping of the C4-isomeric hydroxyl group mediates loss of polar interaction with the key sugar-specific Asp103 polar sidechain and allows significant drift of ligand’s aromatic ring from the hinge region. Adopting a more favored orientation while maintaining as much polar contact as possible with Asp103, the sugar part depicted an inverted conformation obtaining lower energy state. Unlike quercetin-3-*O*-*β*-galactoside, the ligand depicted favored the anchoring of its chromone nucleus towards the hinge region, and its C4 α-isomeric OH group became a favored position relative to Thr23 polar sidechain. Double polar interactions were depicted for the 7-OH with the mainchains of Cys99 and Glu97 hinge residues, whereas hydrophobic contact π-H interaction) with Leu21 was also depicted with ringBchromone (4.9 ± 0.52 Å). Despite the favored orientations of both sugar moiety and chromone ring at the ATP-binding site, the substituted phenyl ring showed limited entry at the buried hydrophobic site with single stability-mediating interaction at Asn28 sidechain. This was obvious since higher RMSD values (1.98 ± 1.21 Å) were depicted for isoquercitrin relative to crystalline ligand, KSA, inferring less superimposition. Thus, furnishing the lower range of polar contacts with pocket residues and limited anchoring at the hydrophobic site caused isoquercitrin to depict lower docking score (−6.08 ± 0.61 Kcal/mol), as compared to quercetin-3-*O*-*β*-galactoside.

Moving towards the higher sugar ligands, rutin showed unfavored orientation of its aromatic pharmacophoric features towards the ATP-binding pocket. Possessing a double glycosylation scaffold (α-L-rhamnopyranosyl-(1→6)-β-D-glucopyranose), the rutin ligand imposed reasonable steric hinderances at the ATP-binding site particularly via its terminal α-L-rhamnopyranosyl. The latter sugar part was directed towards the solvent exposed site, forming a single far range polar contact with the sidechain of Asn109 at the pocket entrance (Figure 8C). Such orientation drags the central β-D-glucopyranose and bishydroxylated aromatic ring, causing non-optimal orientation and a significant shift for these moieties at the sugar-specific and hinge region, respectively. Notably, the central sugar group depicted no polar interaction with the surrounding pocket residues, while only the para-OH of the phenyl ring illustrated single hydrogen bonding with the hinge key residue, Cys99 mainchain. Stability of the aromatic ring was further mediating by hydrophobic contact with Leu21 (4.4 ± 0.51 Å) /Val29 (4.2 ± 0.18 Å), as well as polar contacts with Asp103, where the latter further emphasizes on the ligand’s significant shift. Moreover, the chromone ring showed limited anchoring at the pocket’s hydrophobic cleft, showing single weak hydrogen bonding with the mainchain of the Asp166 residue (Supplementary Table S6). Based on the collective rutin-pocket binding mode, lower docking scores were assigned for this bisglycosylated flavanol (−6.73 ± 0.53 Kcal/mol), as compared to those of the crystalline ligand and quercetin-3-*O*-*β*-galactoside.

Steric hinderance imposed by the α-L-rhamnopyranosyl terminal sugar part was also depicted for the quercetin-3-neohesperidoside binding pose. The double glycosylated ligand showed a highly shifted orientation at all different ATP-binding subpockets, which corresponded to high RMSD values in relation to the crystalline ligand (2.52 ± 0.43 Å). Both the chromone ring and dihydroxy substituted phenyl were settled at the hinge region with the later phenyl substitution being directed towards the solvent exposed entrance of the pocket, and no hydrophobic interactions were depicted for both rings (Figure 8D). Depicting higher shifted ligand orientation in regard to rutin could be reasoned for the more sterically hindered glycosidic linkage between both sugar parts which was (1→2) rather than (1→6). Similar to rutin, the terminal sugar part was directed towards the solvent exposed site, forming a single far range polar contact with the sidechain of Lys106 at the pocket entrance. Stability of the ligand’s aromatic ring was mediated with polar interactions with key hinge region residues, Glu97 and Cys99 mainchains. Meanwhile, the stability of the central α-D-galactopyranose moiety was furnished via polar contacts with the key polar residue Asp103 sidechain. All above findings correlate with poor anchoring of the chromone ring at the deep hydrophobic pocket, which finally give rise to very low docking score (-5.94 ± 0.55 Kcal/mol), being inferior to all investigated flavanols.

Finally, the non-glycosylated flavanol quercetin showed favored orientation at both the hinge and hydrophobic regions, owing to their unrestrained orientation, since the ligand lacks a sugar moiety. Quercetin exhibited the lowest RMSD value (1.04 ± 0.53 Å), inferring significant superimposition with the pharmacophoric features of the crystalline ligand and comparable binding mode (Figure 8E). Actually, the docked chromone ring of the docked ligand were preferentially anchored near the hinge region furnishing double hydrogen bonding with the key hinge region residue, Cys99. Further stability of the chromone ring was mediated through hydrophobic contact with pocket residue, owing to ring’s close proximity towards Leu21 sidechain (4.2 ± 0.63 Å). The latter chromone-hinge region orientation permitted the anchoring of the substituted phenyl ring deep into the hydrophobic pocket. Predicting a planar orientation within the hydrophobic cleft allowed the phenyl ring to exhibit significant stability, with minimal steric clashes with the pocket lining residues. Polar contacts with key pocket residues, including Asn28 and Asp166, allowed relevant specificity for binding at the hydrophobic pocket. Notably, these quercetin-pocket binding features were translated into moderate docking score (-6.57 ± 0.71 Kcal/mol) which was better that a couple of the investigated compounds, including the glycosylated flavanols quercetin-3-neohesperidoside and isoquercitrin, the thing that was correlated to better biological activity. As for the final debate, the combined in silico findings from both performed docking studies illustrated the differential binding modes of the investigated compounds towards two biological targets of the NF-κB pathway. These obtained differential binding patterns have provided useful insights regarding structural activity requirements, serving as guiding tools for future lead development and optimization, speeding up the pipeline discovery of novel anti-inflammatory agents from the natural resource.

## 4. Conclusions

In conclusion, the utilization of various chromatographic techniques led to the isolation of seven compounds from the *C. acutum* plant extract, including a coumarin and six flavonoid compounds. The antioxidant and anti-inflammatory properties of five of the isolated flavonoids (i.e., quercetin-3-*O*-*β*-galactoside, quercetin, rutin, quercetin-3-O-neohesperdoside and isoquercitrin) were assessed in a rat model of T2DM. All the investigated compounds ameliorated the oxidative stress, reduced the levels of inflammatory markers, and regulated the expression of NF-κB and miR-146a in both liver and adipose tissue. The furnished findings from the molecular docking simulation on two significant NF-κB targets; RelB:p52 heterodimer and hIKK, have predicted the binding preferentiality for quercetin-3-*O*-*β*-galactoside, even as compared to the crystallized ligand at the hIKK protein target. Furthermore, the docking investigation highlights the molecular promiscuity of the isolated flavonoids, allowing them to modulate multiple molecular pathways and targets as well. Conformation of the obtained in silico findings could be done through experimental studies, testing the effect of compounds on NF-κB, by assessing the phosphorylation and total levels of p65 and IKK by Western blotting. The latter could be considered as a limitation of the presented study. Thus, further experimental studies would be conducted in the future to confirm the docking findings, while additionally conducted tests would be valuable to highlight different potential targets that would solve the missing pieces, explaining the flavonoid-oriented antioxidant and anti-inflammatory bioactivities.

## Figures and Tables

**Figure 1:**
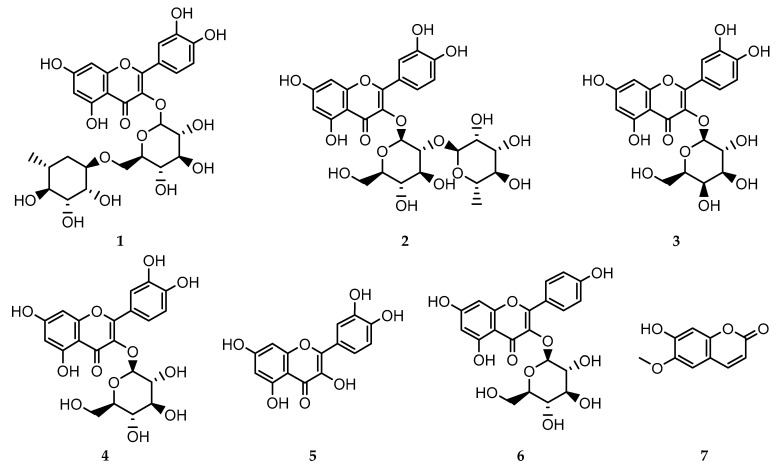
Structure of isolated compounds **1**–**7**.

**Table 1:** Primers and annealing temperatures used in real-time PCR.

GenBank Accession No.	Gene	Primers	Annealing Temperature
NM_199267.2	NF-κB	Forward: 5′-CAATGGCTACACAGGACCA-3′	55 °C
		Reverse: 5′-CACTGTCACCTGGAACCAGA-3′	
NM_012675.3	TNF-α	Forward: 5′-TCTACTGAACTTCGGGGTGATCG-3′	60 °C
		Reverse: 5′-TGATCTGAGTGTGAGGGTCTGGG-3′	
NM_013076.3	Leptin	Forward: 5′-GACATTTCACACACGCAGTC-3′	57 °C
		Reverse: 5′-GAGGAGGTCTCGCAGGTT-3′	
NM_144744.3	Adiponectin	Forward: 5′-AATCCTGCCCAGTCATGAAG-3′	56 °C
		Reverse: 5′-CATCTCCTGGGTCACCCTTA-3′	
NM_017008.4	GAPDH	Forward: 5′-ATGACTCTACCCACGGCAAG-3′	56 °C
		Reverse: 5′-GATCTCGCTCCTGGAAGATG-3′	

**Table 2:** The effect of the isolated compounds on fasting blood glucose, insulin, and insulin resistance indices in the experimental rats.

Groups	FBG (mg/dL)	Insulin (μIU/mL)	HOMA-IR	QUICKI
Normal	93.2 ± 9.26	10.89 ± 2.85	2.53 ± 0.79	0.33 + 0.03
Control (HFD + STZ)	277.28 ± 28.17 *	44.17 ± 4.71 *	30.09 ± 4.30 *	0.24 ± 0.03 *
C1 (Rutin)	218.07 ± 23.37 *^#^	34.64 ± 4.83 *^#^	18.84 ± 3.00 *^#^	0.26 ± 0.02 *
C2 (Quercetin 3-*O*-neohesperdoside)	251.34 ± 25.48 *	39.92 ± 4.92 *	24.79 ± 3.87 *	0.25 ± 0.02 *
C3 (Quercetin-3-*O*-*β*-galactoside)	120.93 ± 12.83 *^#^	19.21 ± 3.09 *^#^	5.68 ± 1.03 *^#^	0.30 ± 0.02 *^#^
C4 (Isoquercetrin)	262.25 ± 27.30 *	41.66 ± 4.48 *	27.17 ± 3.01 *	0.25 ± 0.02 *
C5 (Quercetin)	198.39 ± 20.37 *^#^	31.51 ± 3.98 *^#^	15.16 ± 2.04 *^#^	0.26 ± 0.03 *

Data are expressed as mean ± SD and analyzed using one-way ANOVA followed by Bonferroni’s post hoe test (n = 6-8). FBG = fasting blood glucose; HOMA-IR = homeostasis model assessment-insulin resistance; QUICKI = quantitative insulin sensitivity check index; * significantly different compared to the normal group at *p* < 0.05; at *p* < 0.001; ^#^ significantly different compared to the T2DM control group (high fat diet + Streptozotocin) at *p* < 0.05.

**Table 3:** The effect of the isolated compounds on the body weight, adipose tissue index and lipid profile of the experimental rats.

Groups	% Increase in Body Weight	Adipose Tissue Index %	TG (mg/dL)	TC (mg/dL)	LDL-C (mg/dL)	HDL-C (mg/dL)
Normal	61.90 ± 6.11	2.19 ± 0.33	192.19 ± 20.18	116.58 ± 13.98	56.97 ± 6.28	40.39 ± 4.27
Control (HFD + STZ)	189.02 ± 23.17 *	5.42 ± 0.44 *	284.68 ± 29.38 *	243.72 ± 28.01 *	180.78 ± 20.21 *	34.47 ± 3.21 *
C1 (Rutin)	146.97 ± 16.20 *^#^	4.21 ± 0.39 *^#^	221.62 ± 23.39 *^#^	146.73 ± 16.02 *^#^	86.54 ± 9.20 *^#^	38.03 ± 4.92 ^#^
C2 (Quercetin 3-*O*-neohesperdoside)	162.90 ± 17.00 *	4.67 ± 0.52 *	245.65 ± 25.23 *	148.24 ± 16.07 *^#^	86.97 ± 10.25 *^#^	36.71 ± 4.02
C3 (Quercetin-3-*O*-*β*-galactoside)	140.60 ± 13.29 *^#^	4.03 ± 0.40 *^#^	212.01 ± 24.20^#^	120.6 ± 12.45 ^#^	59.53 ± 6.39 ^#^	39.87 ± 3.01 ^#^
C4 (Isoquercetrin)	177.25 ± 19.83 *	5.08 ± 0.61 *	267.28 ± 28.31 *	177.89 ± 19.38 *^#^	115.11 ± 13.21 *^#^	36.05 ± 4.22
C5 (Quercetin)	142.59 ± 15.02 *^#^	4.09 ± 0.42 *^#^	215.02 ± 20.13^#^	126.63 ± 14.30 ^#^	65.39 ± 7.37 ^#^	39.74 ± 4.28 ^#^

Data are expressed as mean ± SD and analyzed using one-way ANOVA followed by Bonferroni’s post hoc test (*n* = 6–8). TG = triglycerides; TC = total cholesterol; LDL-C = low density lipoprotein-cholesterol; HDL-C = high density lipoprotein-cholesterol; * significantly different compared to the normal group at *p* < 0.05; ^#^ significantly different compared to the T2DM control group (high fat diet + Streptozotocin) at *p* < 0.05.

**Table 4:** The effect of the isolated compounds on the liver index, liver enzymes, and liver content of lipids in the experimental rats.

Groups	Liver Index %	ALT (IU/L)	AST (IU/L)	Liver TG (mg/g)	Liver TC (mg/g)
Normal	2.55 ± 0.25	49.27 ± 5.70	68.83 ± 7.45	5.90 ± 0.88	3.20 ± 0.52
Control (HFD + STZ)	3.51 ± 0.28 *	77.12 ± 8.13 *	91.73 ± 9.32 *	14.33 ± 2.90 *	7.80 ± 1.00 *
C1 (Rutin)	2.98 ± 0.15 *^#^	63.43 ± 6.80 *^#^	78.86 ± 8.47	8.50 ± 1.59 *^#^	5.32 ± 0.71 *^#^
C2 (Quercetin 3-*O*-neohesperdoside)	3.14 ± 0.16 *	67.85 ± 7.47 *^#^	82.48 ± 8.25 *	10.48 ± 1.99 *^#^	5.55 ± 0.82 *^#^
C3 (Quercetin-3-*O*-*β*-galactoside)	2.66 ± 0.14^#^	54.68 ± 6.30^#^	71.62 ± 7.33 ^#^	7.60 ± 1.01 ^#^	4.44 ± 0.62 ^#^
C4 (Isoquercetrin)	3.30 ± 0.17 *	72.21 ± 7.49 *	86.10± 9.08 *	11.35 ± 2.20 *^#^	5.81 ± 0.81 *^#^
C5 (Quercetin)	2.82 ± 0.14 *^#^	59.00 ± 6.50 ^#^	75.24 ± 8.09 ^#^	8.10 ± 1.57 *^#^	4.92 ± 0.67 *^#^

Data are expressed as mean ± SD and analyzed using one-way ANOVA followed by Bonferroni’s post hoe test (*n* = 6–8). ALT = alanine aminotransferase; AST = aspartate aminotransferase; TG = triglycerides; TC = total cholesterol; * significantly different compared to the normal group at *p* < 0.05; ^#^ significantly different compared to the T2DM control group (high fat diet + Streptozotocin) at *p* < 0.05.

## Data Availability

Data are contained within the article and the Supplementary Material.

## Supplementary Materials

The following are available online at https://www.mdpi.com/article/10.3390/antiox10111713/s1, Figures S1–S3: NMR spectra of compound **1**; Figures S4–S8: NMR spectra of compound **2**; Figures S9–S11: NMR spectra of compound **3**; Figures S12–S14: NMR spectra of compound **4**; Figures S15–S17: NMR spectra of compound **5**; Figures S18–S20: NMR spectra of compound **6**; Figures S21–S23: NMR spectra of compound **7**; Figure S24: The architecture of NF-κB RelB:p52:DNAheterocomplex, Figure S25: The architecture of human inhibitory-κB kinase β (hIKKβ) in complex with inhibitor, Table S1: The effect of the crude extract and fractions of *Cynanchum acutum* on fasting blood glucose, insulin and insulin resistance indices in the experimental rats; Table S2: The effect of the crude extract and fractions of *Cynanchum acutum* on the body weight, adipose tissue index and lipid profile of the experimental rats; Table S3: The effect of the crude extract and fractions of *Cynanchum acutum* on the liver index and liver enzymes of the experimental rats; Table S4: The average daily food intake of the experimental rats (g/rat/day) in each group throughout the duration of the study. Table S5: The In silico findings of ligand-docking studies at the DNA-specific binding domain of the NF-κb RelB:p52 heterodimer; Table S6: The In silico findings of ligand-docking studies at the ATP-binding domain of the hIKK protein target.
