# Supplementary material for: Antioxidant and Anti-Inflammatory Activity of Cynanchum acutum L. Isolated Flavonoids Using Experimentally Induced Type 2 Diabetes Mellitus: Biological and In Silico Investigation for NF-κB Pathway/miR-146a Expression Modulation
Source: Antioxidants (Basel). 2021 Oct 27;10(11):1713. doi: 10.3390/antiox10111713 (PMC8615122; doi:10.3390/antiox10111713)
Supplement: Supplementary file 1 [file antioxidants-10-01713-s001.zip › antioxidants-1398416-supplementary.pdf]

Antioxidant and Anti-inflammatory Activity of *Cynanchum acutum* L. Isolated Flavonoids Using Experimentally Induced Type 2 Diabetes Mellitus: Biological and In Silico Investigation for NF-κB – miR-146a Pathway Modulation

Additional Experimental Detail

1) **Figure S1.** ^{1}H NMR spectrum of compound **1**
2) **Figure S2.** ^{13}C NMR spectrum of compound **1**
3) **Figure S3.** DEPT ^{13}C NMR spectrum of compound **1**
4) **Figure S4.** ^{1}H NMR spectrum of compound **2**
5) **Figure S5.** ^{13}C NMR spectrum of compound **2**
6) **Figure S6.** HSQC spectrum of compound **2**
7) **Figure S7.** ^{1}H- ^{1}H COSY spectrum of compound **2**
8) **Figure S8.** HMBC spectrum of compound **2**
9) **Figure S9.** ^{1}H NMR spectrum of compound **3**
10) **Figure S10.** ^{13}C NMR spectrum of compound **3**
11) **Figure S11.** DEPT ^{13}C NMR spectrum of compound **3**
12) **Figure S12.** ^{1}H NMR spectrum of compound **4**
13) **Figure S13.** ^{13}C NMR spectrum of compound **4**
14) **Figure S14.** DEPT ^{13}C NMR spectrum of compound **4**
15) **Figure S15.** ^{1}H NMR spectrum of compound **5**
16) **Figure S16.** ^{13}C NMR spectrum of compound **5**
17) **Figure S17.** DEPT ^{13}C NMR spectrum of compound **5**
18) **Figure S18.** ^{1}H NMR spectrum of compound **6**
19) **Figure S19.** ^{13}C NMR spectrum of compound **6**
20) **Figure S20.** DEPT ^{13}C NMR spectrum of compound **6**
21) **Figure S21.** ^{1}H NMR spectrum of compound **7**
22) **Figure S22.** ^{13}C NMR spectrum of compound **7**
23) **Figure S23.** DEPT ^{13}C NMR spectrum of compound **7**
24) **Figure S24.** The architecture of NF-κB RelB:p52:DNAheterocomplex
25) **Figure S25.** The architecture of human inhibitory-κB kinase β (hIKKβ) in complex with inhibitor,

26) **Table S1.** The effect of the crude extract and fractions of *Cynanchum acutum* on fasting blood glucose, insulin and insulin resistance indices in the experimental rats
27) **Table S2.** The effect of the crude extract and fractions of *Cynanchum acutum* on the body weight, adipose tissue index and lipid profile of the experimental rats
28) **Table S3.** The effect of the crude extract and fractions of Cynanchum acutum on the liver index and liver enzymes of the experimental rats
29) **Table S4.** The average daily food intake of the experimental rats (g/rat/day) in each group throughout the duration of the study
30) **Table S5.** The In-silico findings of ligand-docking studies at the DNA-specific binding domain of the NF-κB RelB:p52 heterodimer
31) **Table S6:** The In-silico findings of ligand-docking studies at the ATP-binding domain of the hIKK protein target

Compound 1 (rutin):

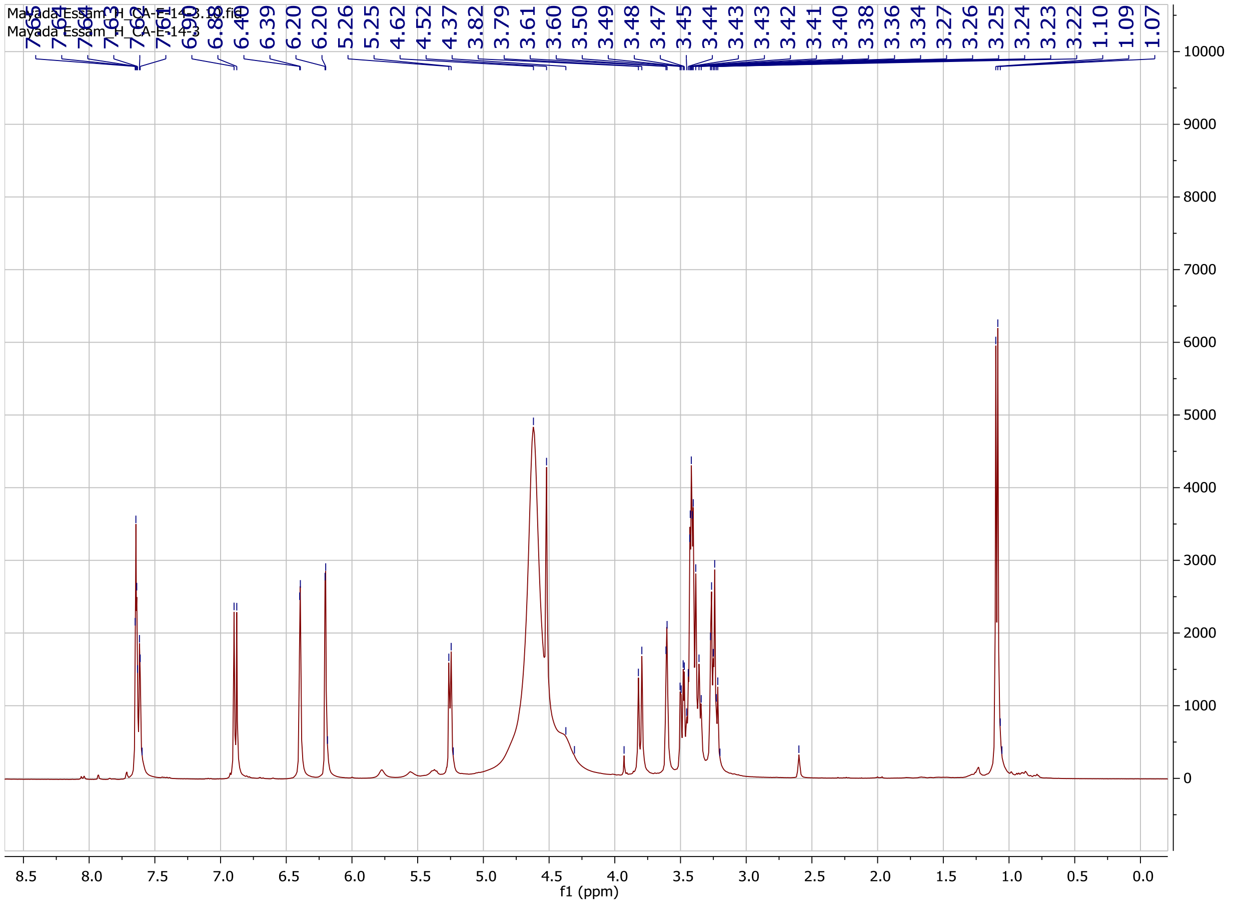

Figure S1. ^{1}H NMR spectrum of compound **1**

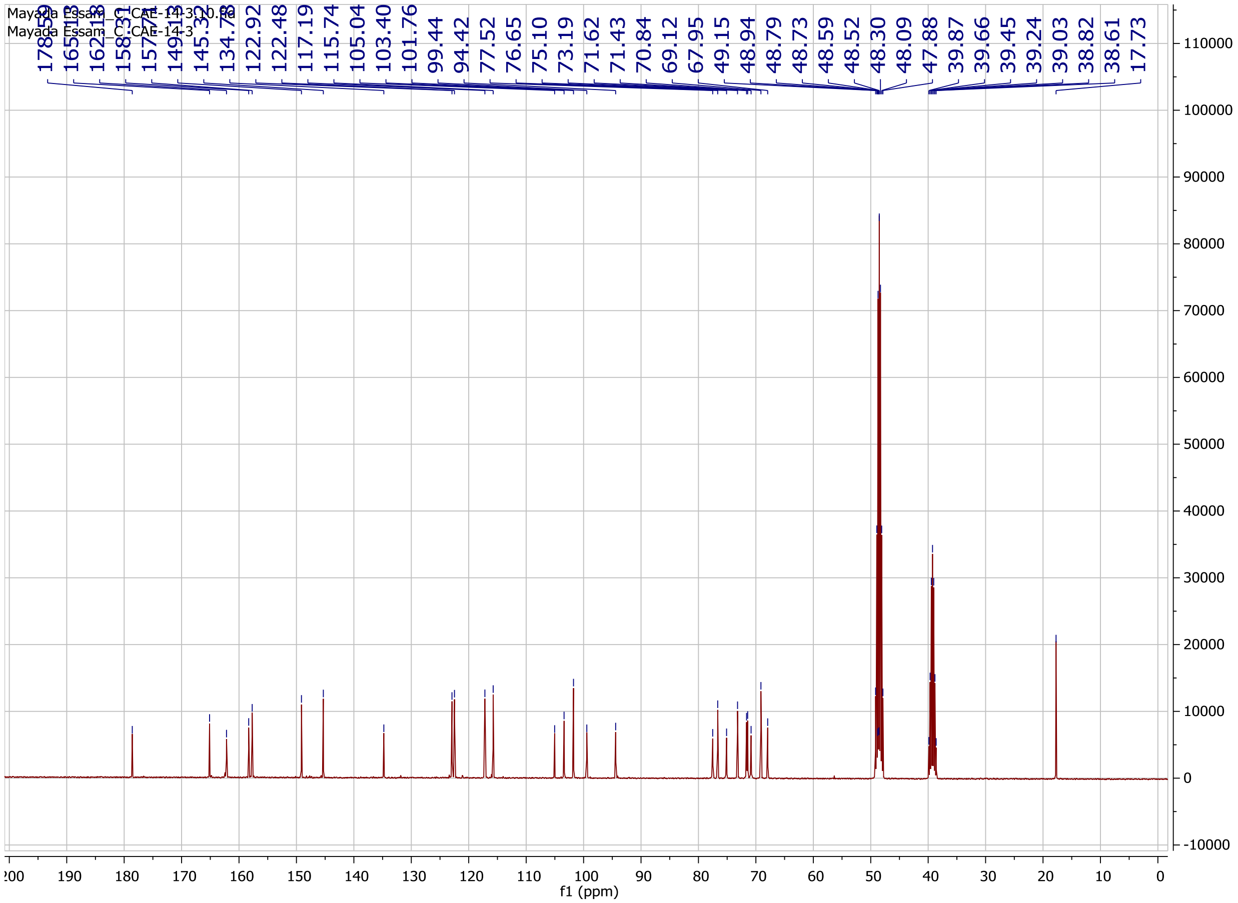

Figure S2. ^{13}C NMR spectrum of compound **1**

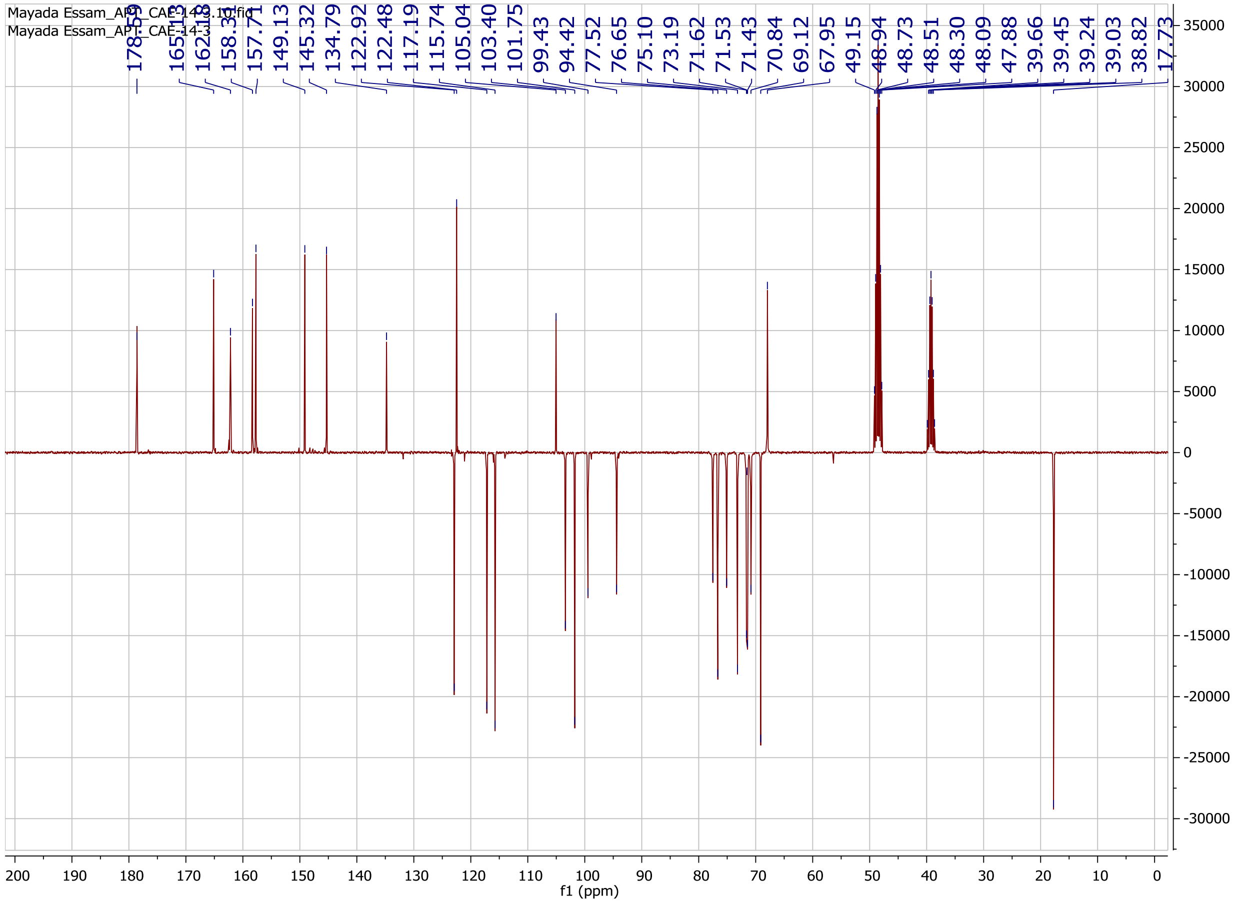

Figure S3. DEPT ^{13}C NMR spectrum of compound **1**

Compound 2 (quercetin-3-*O*-neohesperidoside):

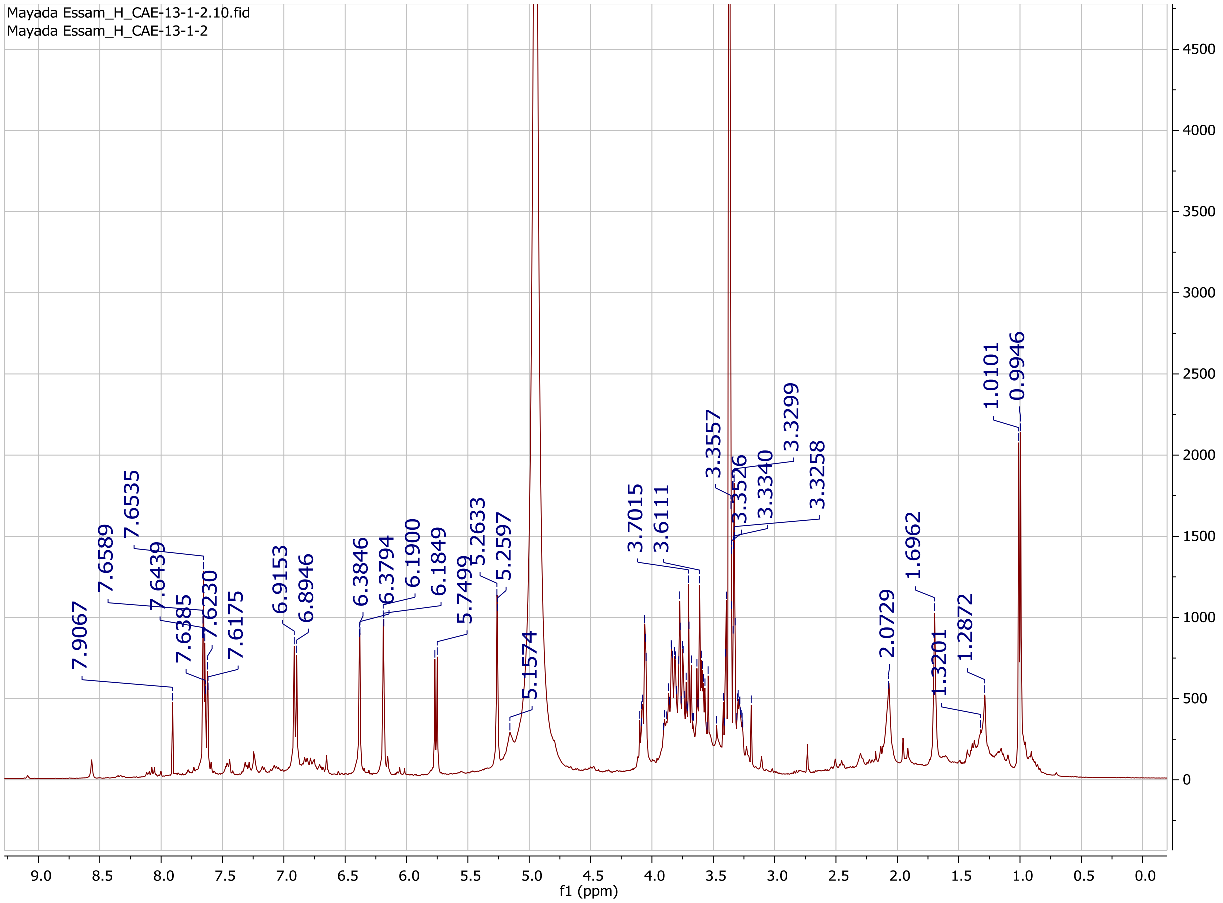

Figure S4. ^{1}H NMR spectrum of compound **2**

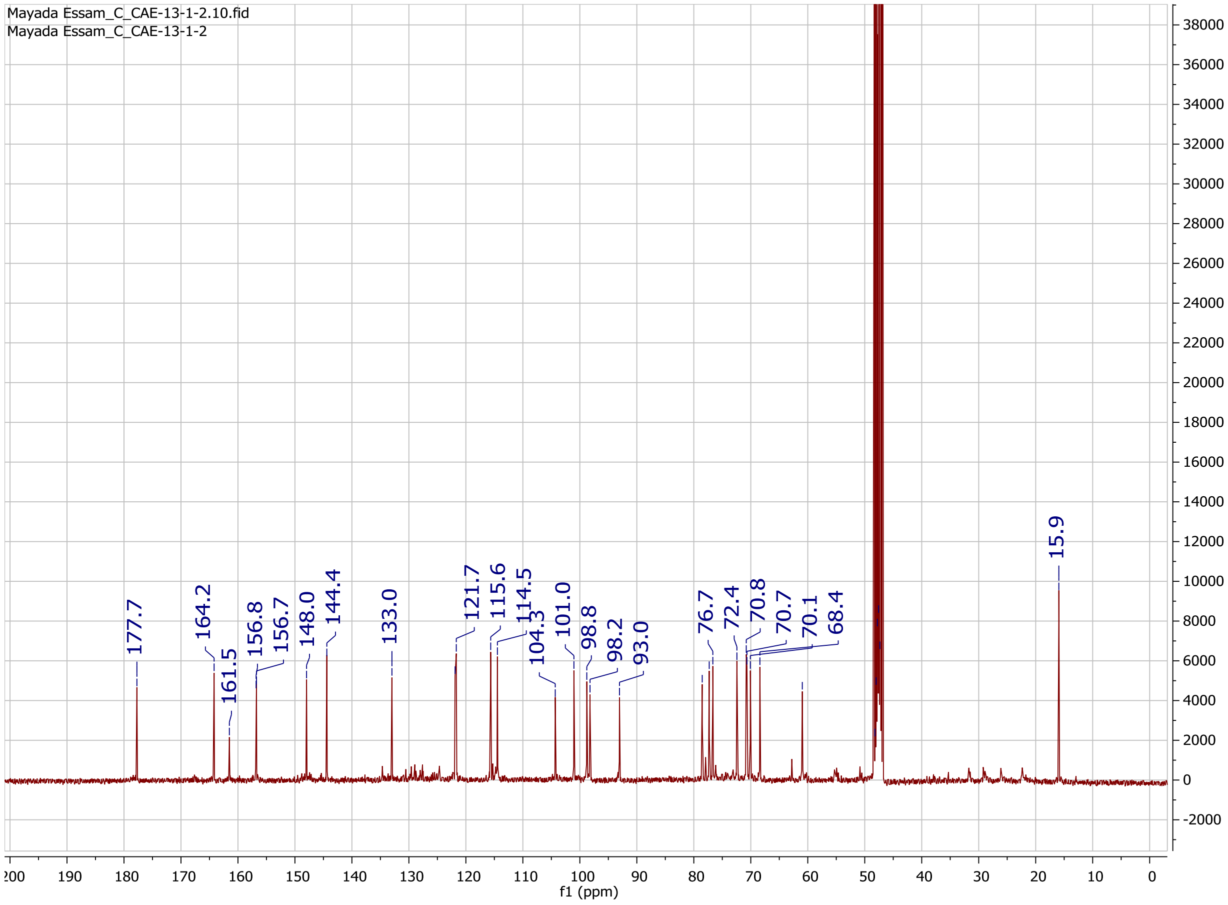

Figure S5. ^{13}C NMR spectrum of compound **2**

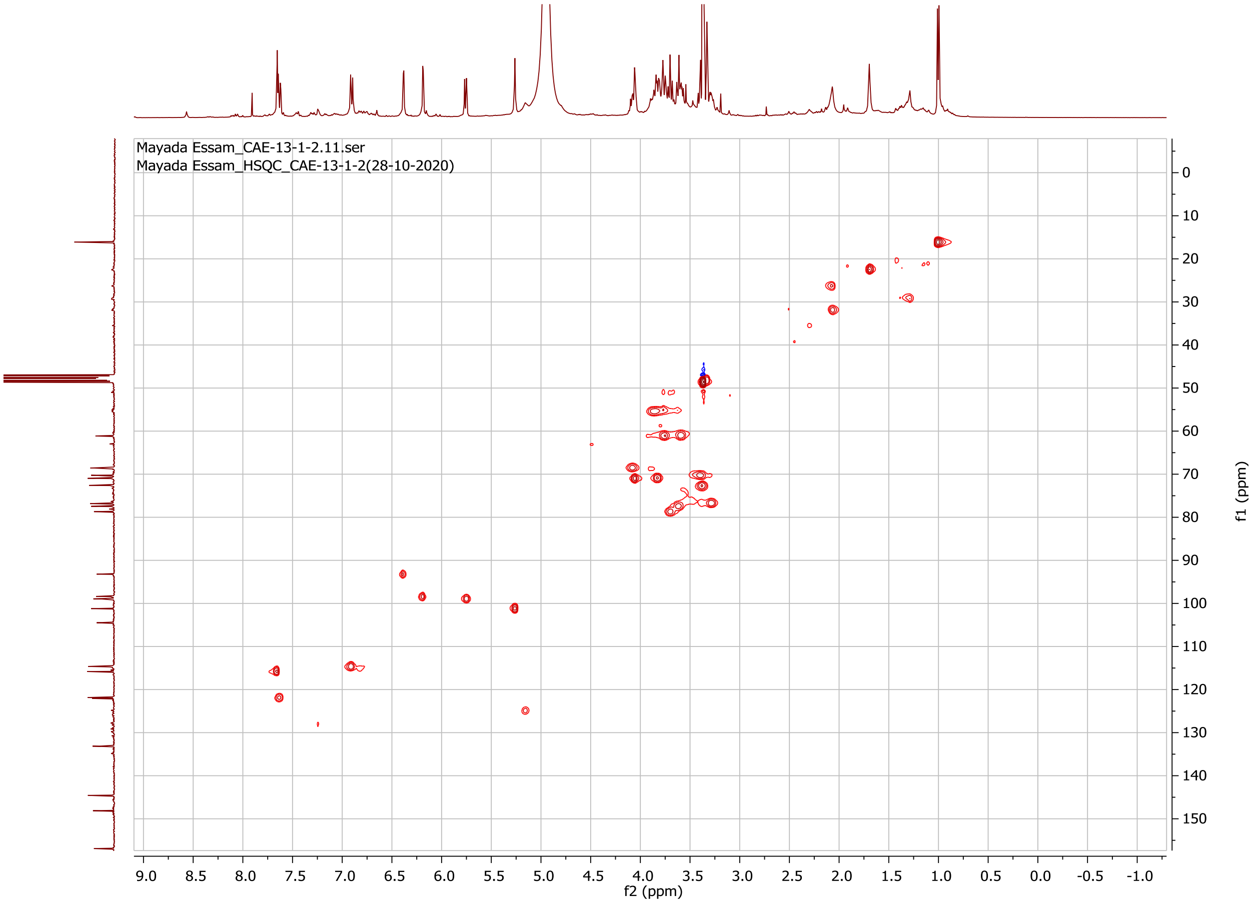

Figure S6. HSQC spectrum of compound **2**

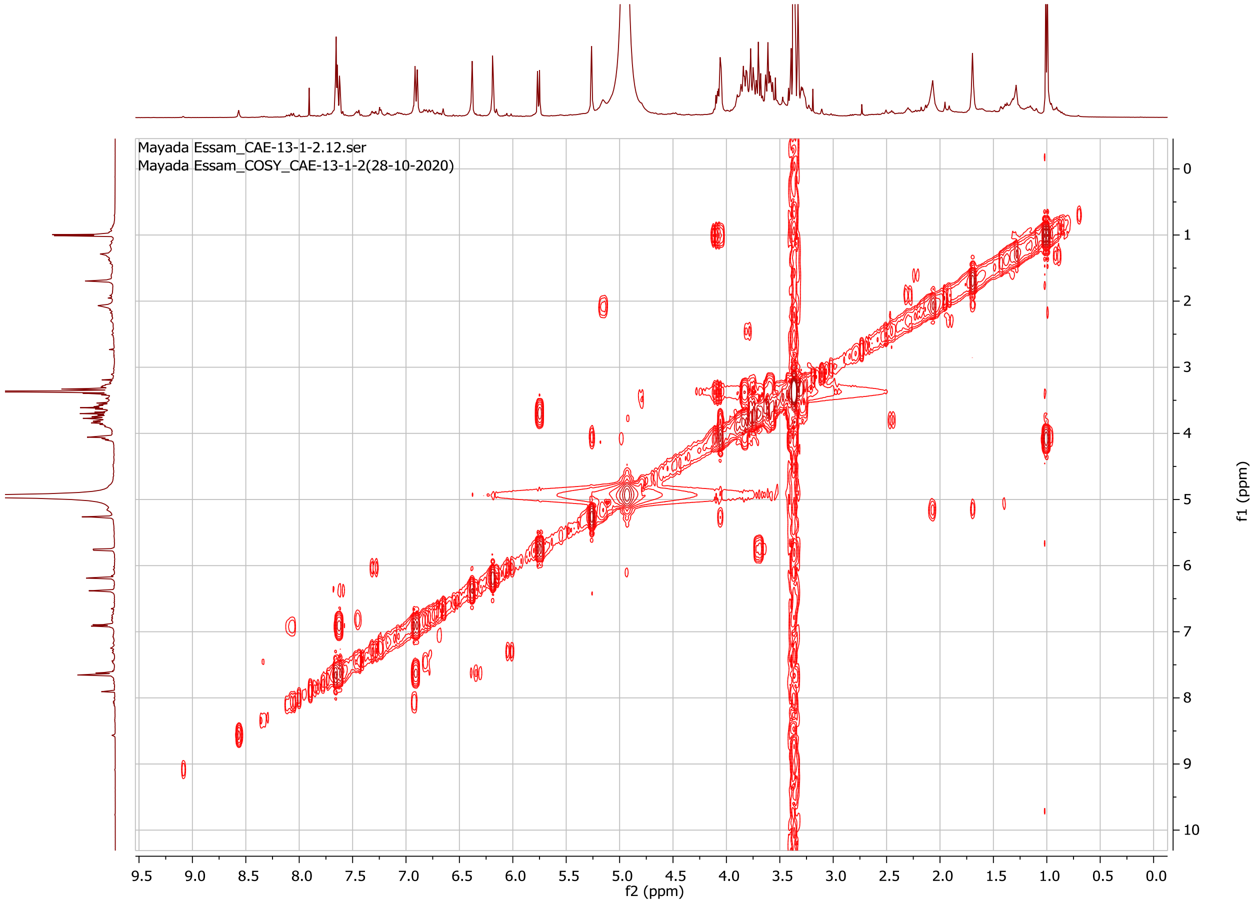

Figure S7. ^{1}H- ^{1}H COSY spectrum of compound **2**

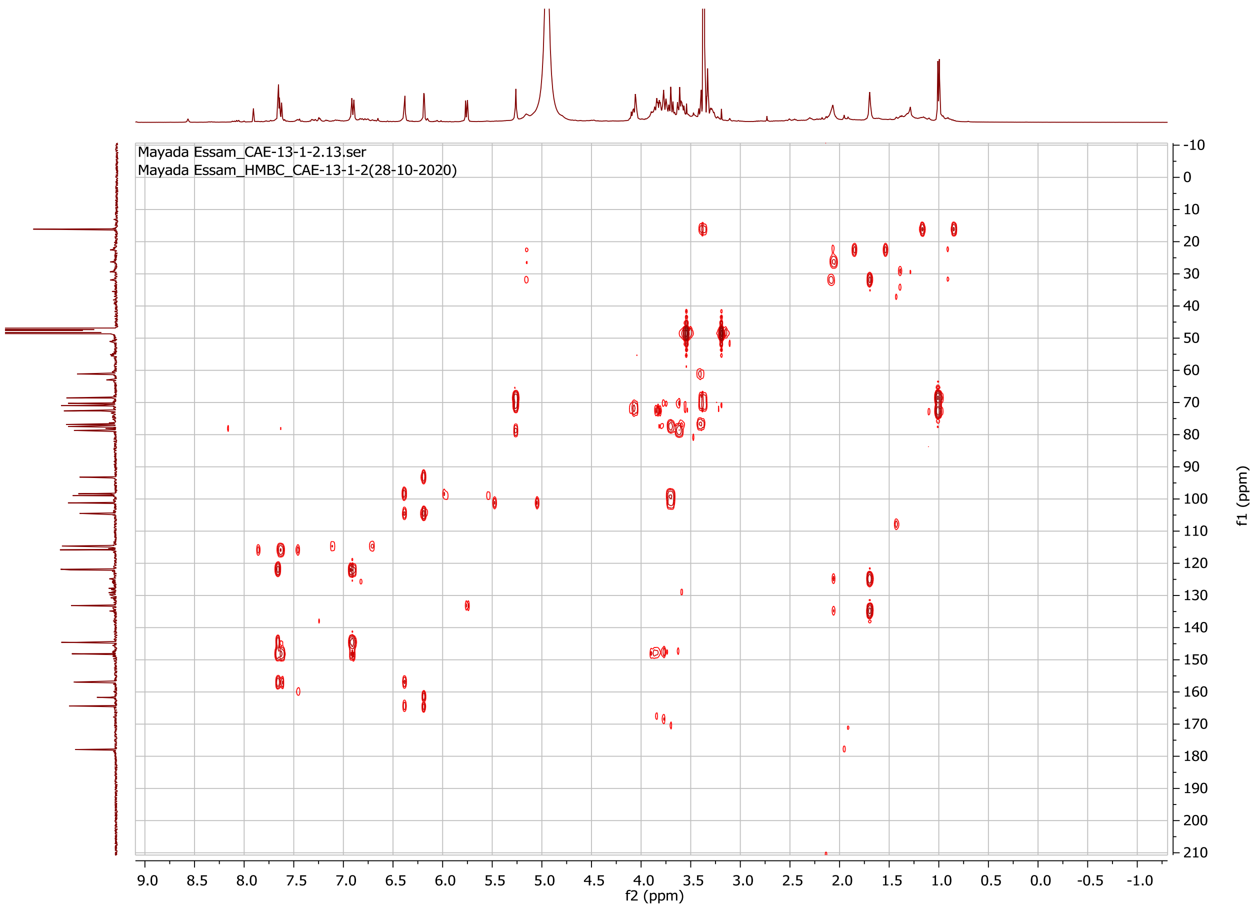

Figure S8. HMBC spectrum of compound **2**

Compound 3 (quercetin-3-*O*-*β*-galactoside):

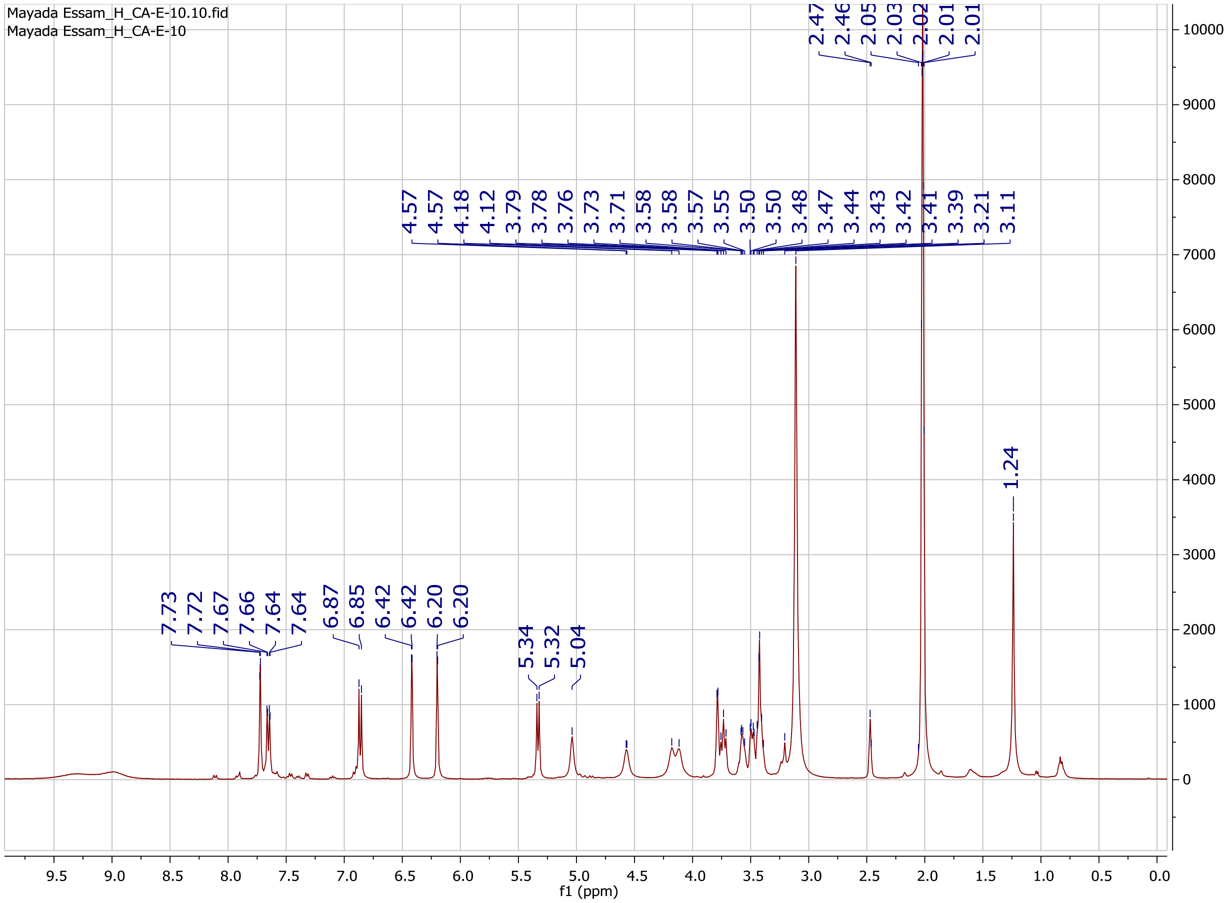

Figure S9. ^{1}H NMR spectrum of compound **3**

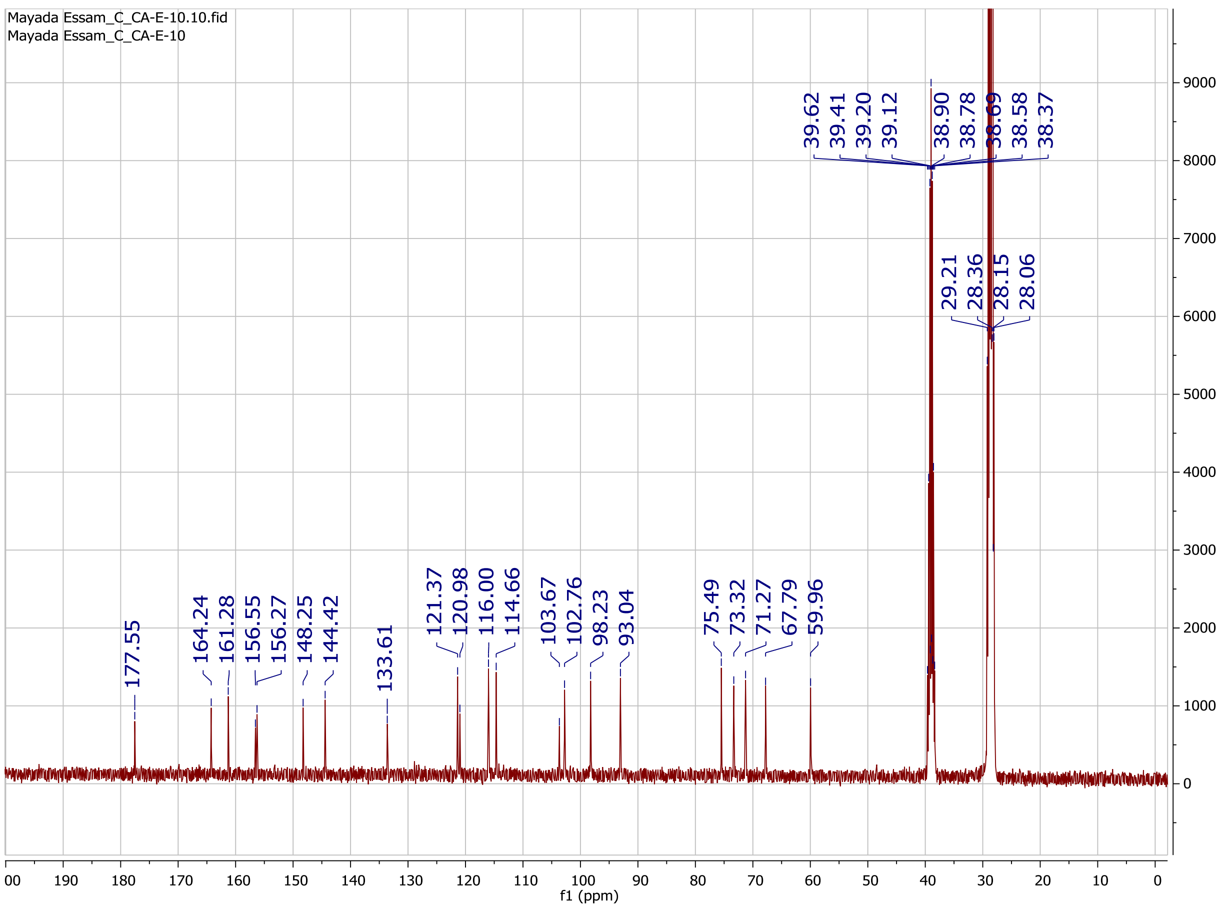

Figure S10. ^{13}C NMR spectrum of compound **3**

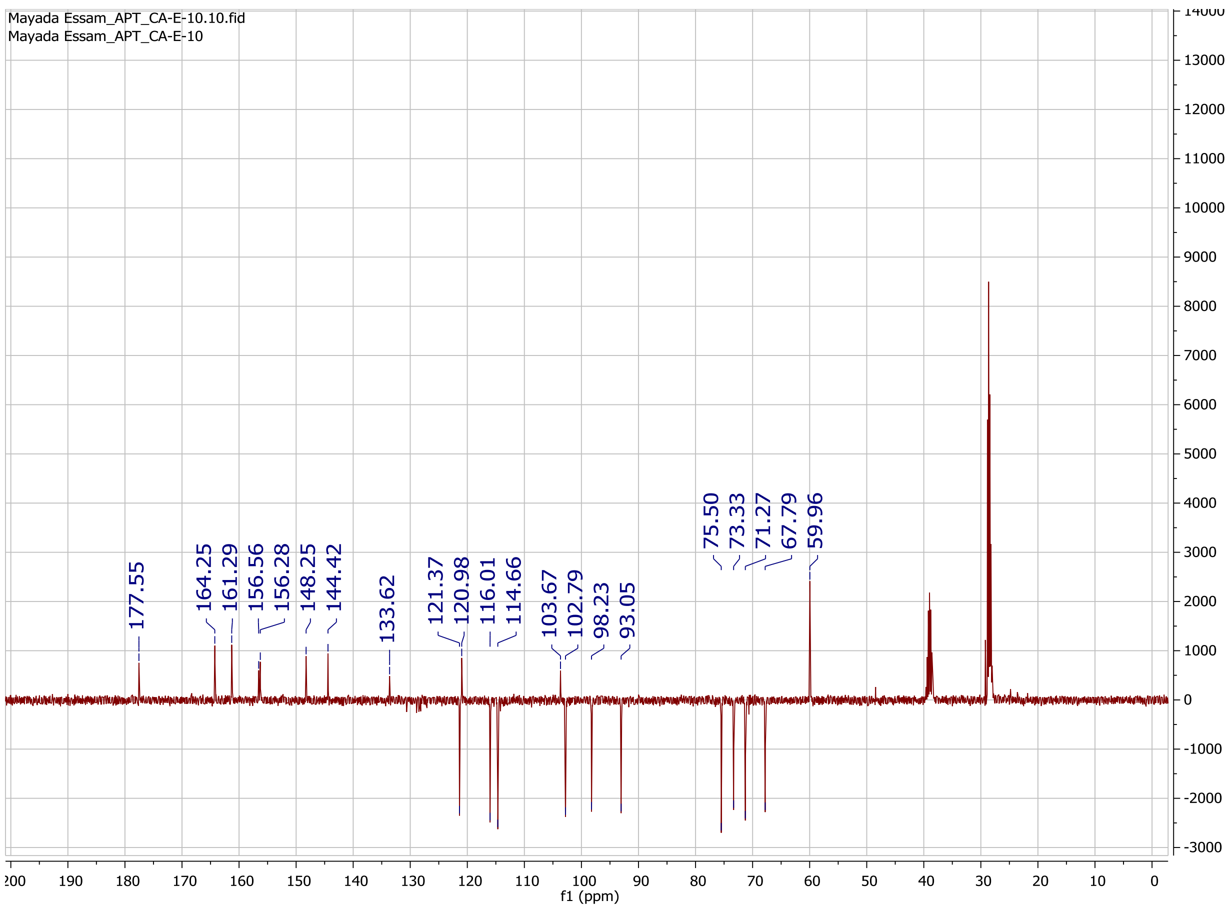

Figure S11. DEPT ^{13}C NMR spectrum of compound **3**

Compound 4 (Isoquercetin):

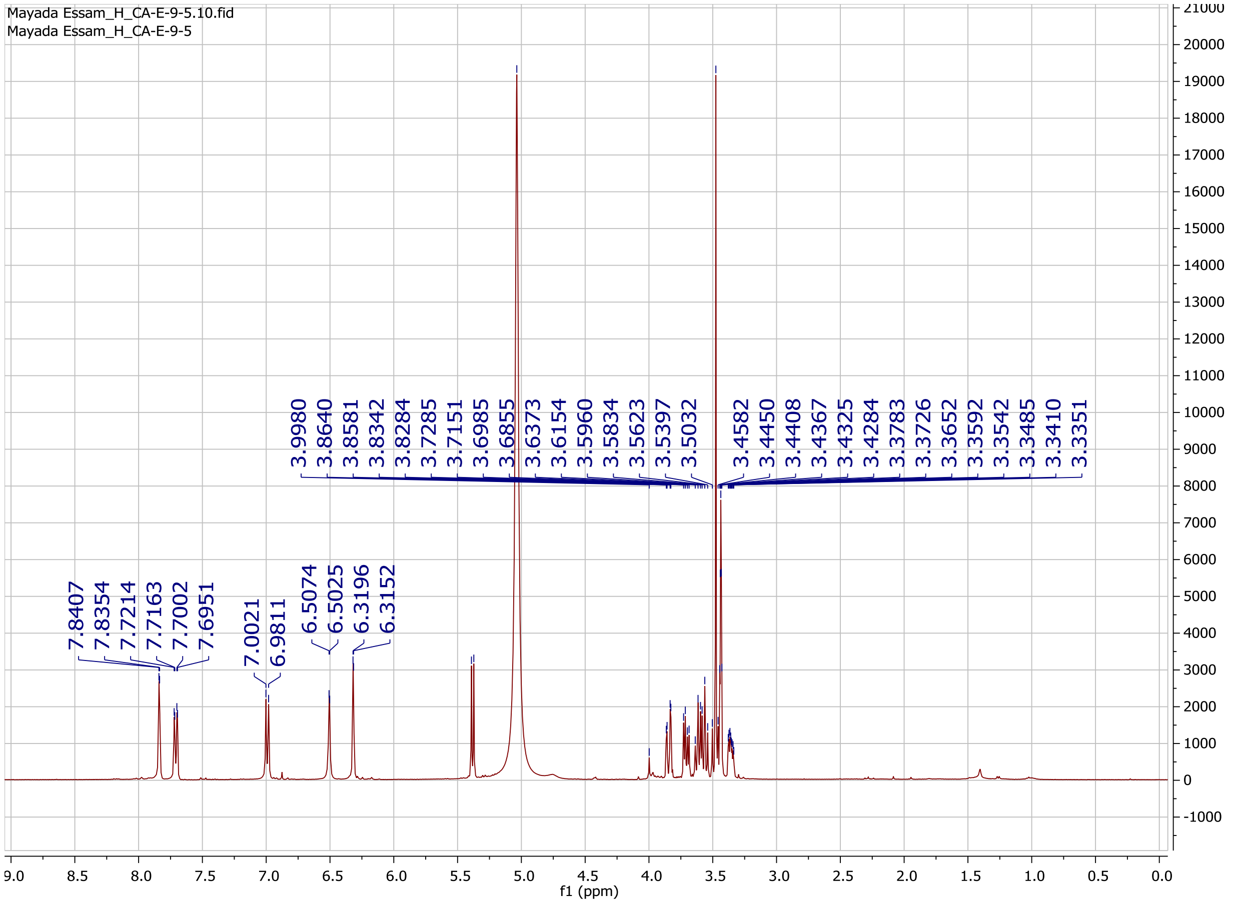

Figure S12. ^{1}H NMR spectrum of compound **4**

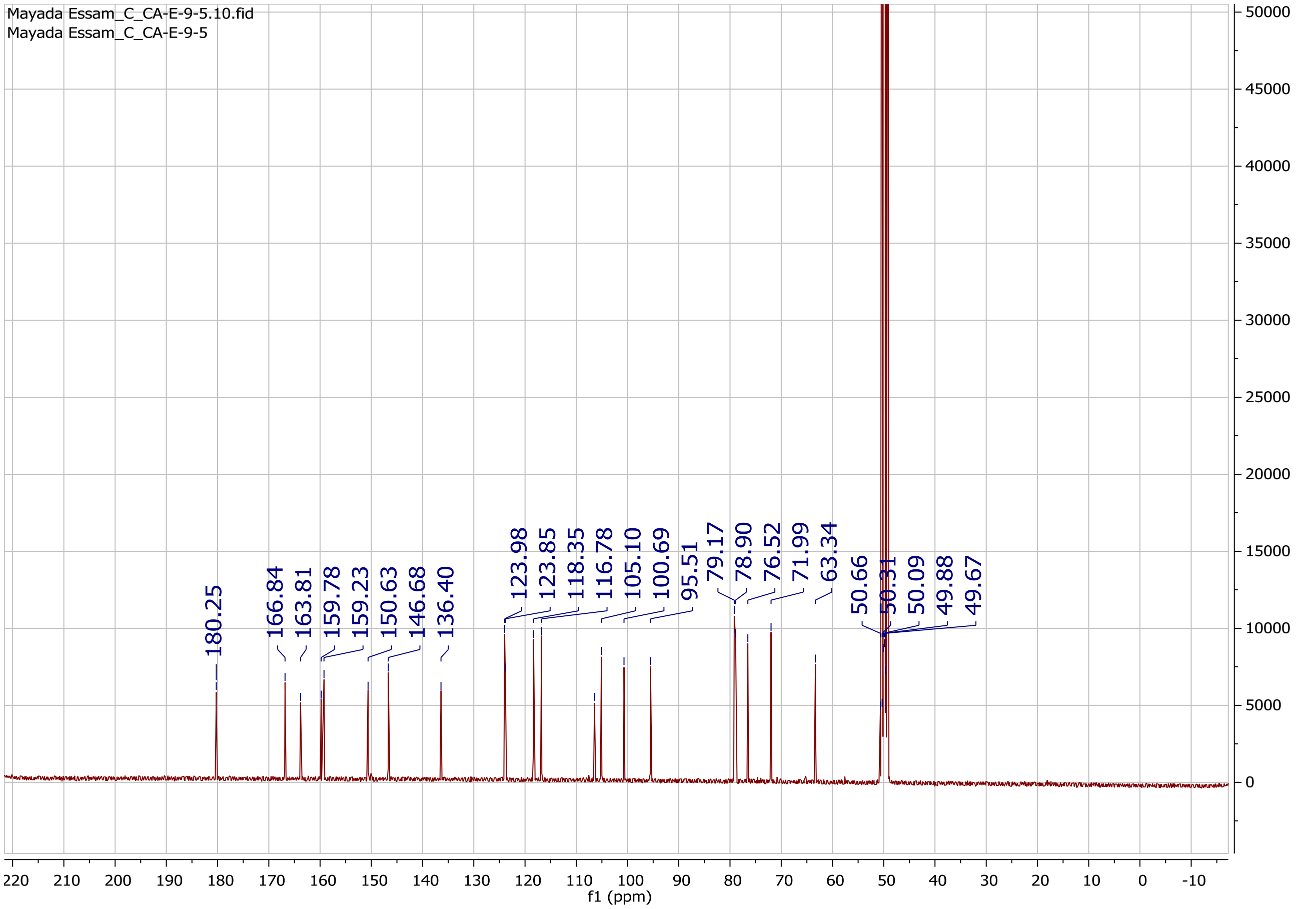

Figure S13. ^{13}C NMR spectrum of compound **4**

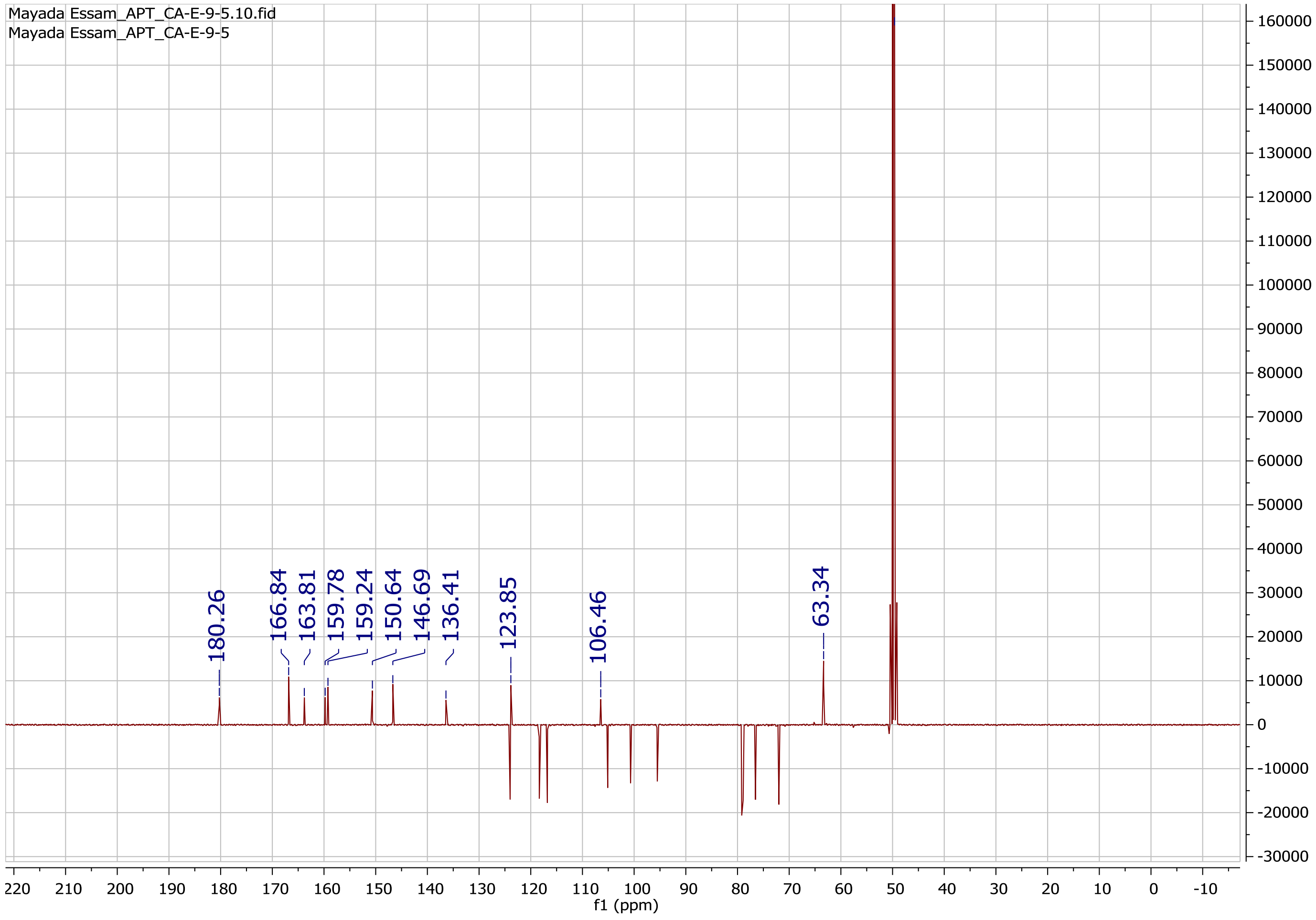

Figure S14. DEPT ^{13}C NMR spectrum of compound **4**

Compound 5 (quercetin):

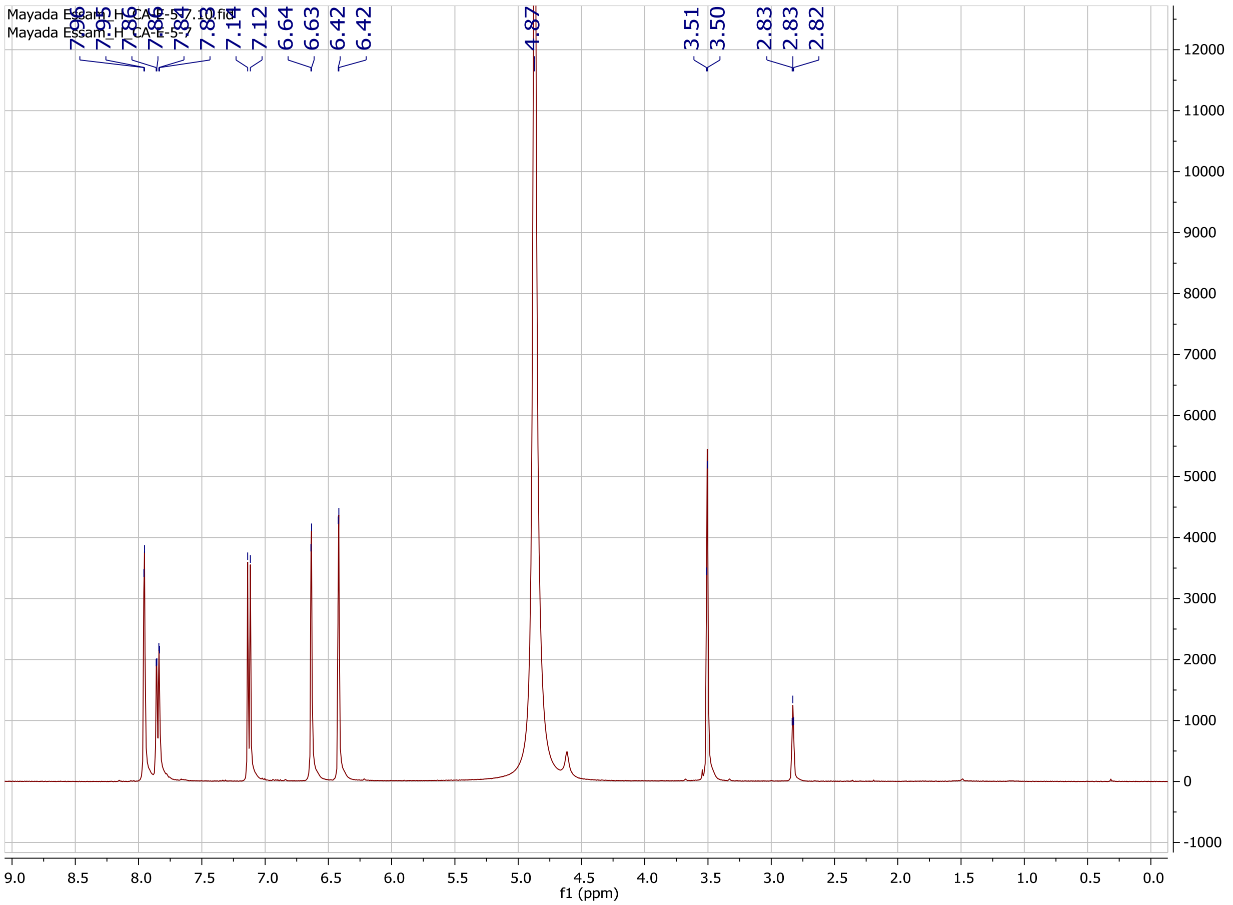

Figure S15. ^{1}H NMR spectrum of compound **5**

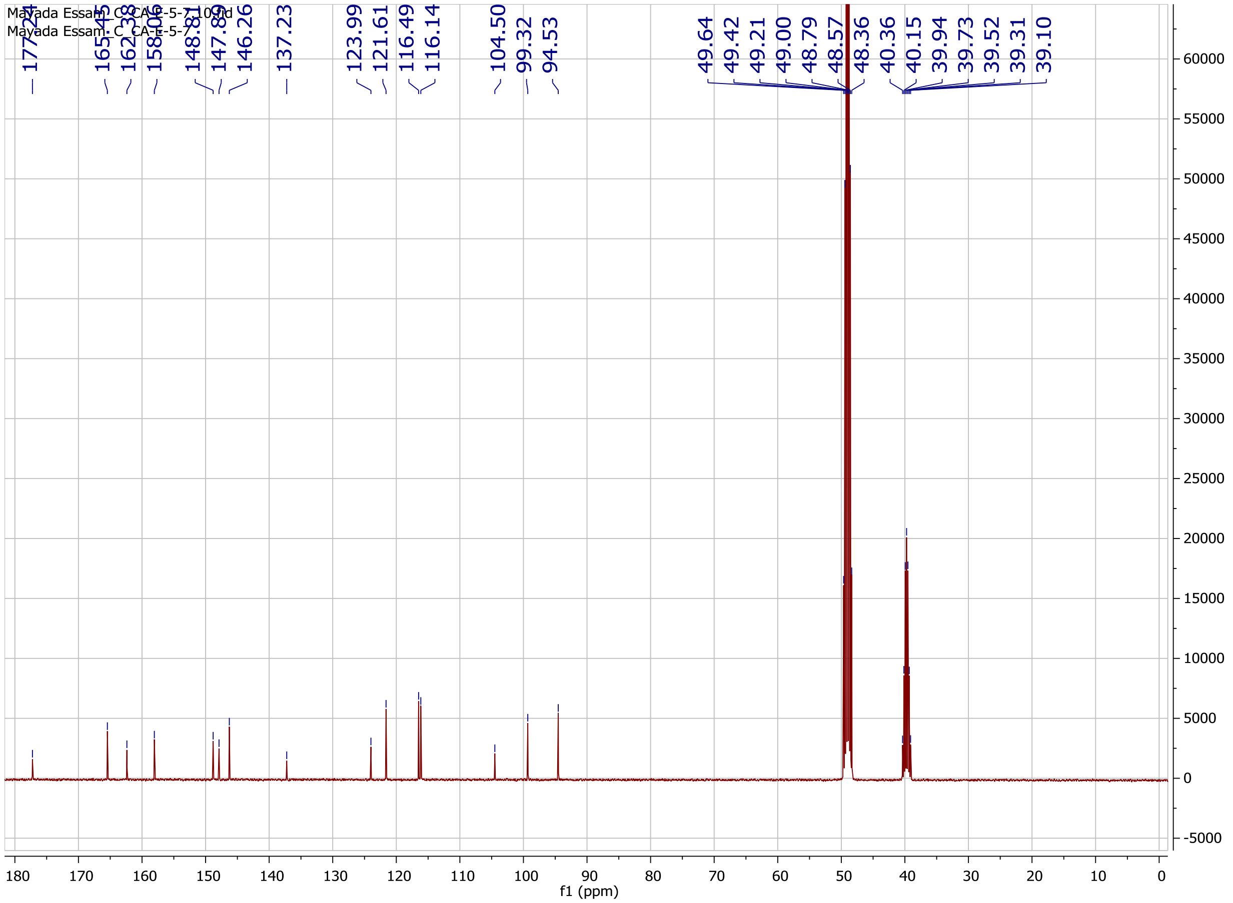

Figure S16. ^{13}C NMR spectrum of compound **5**

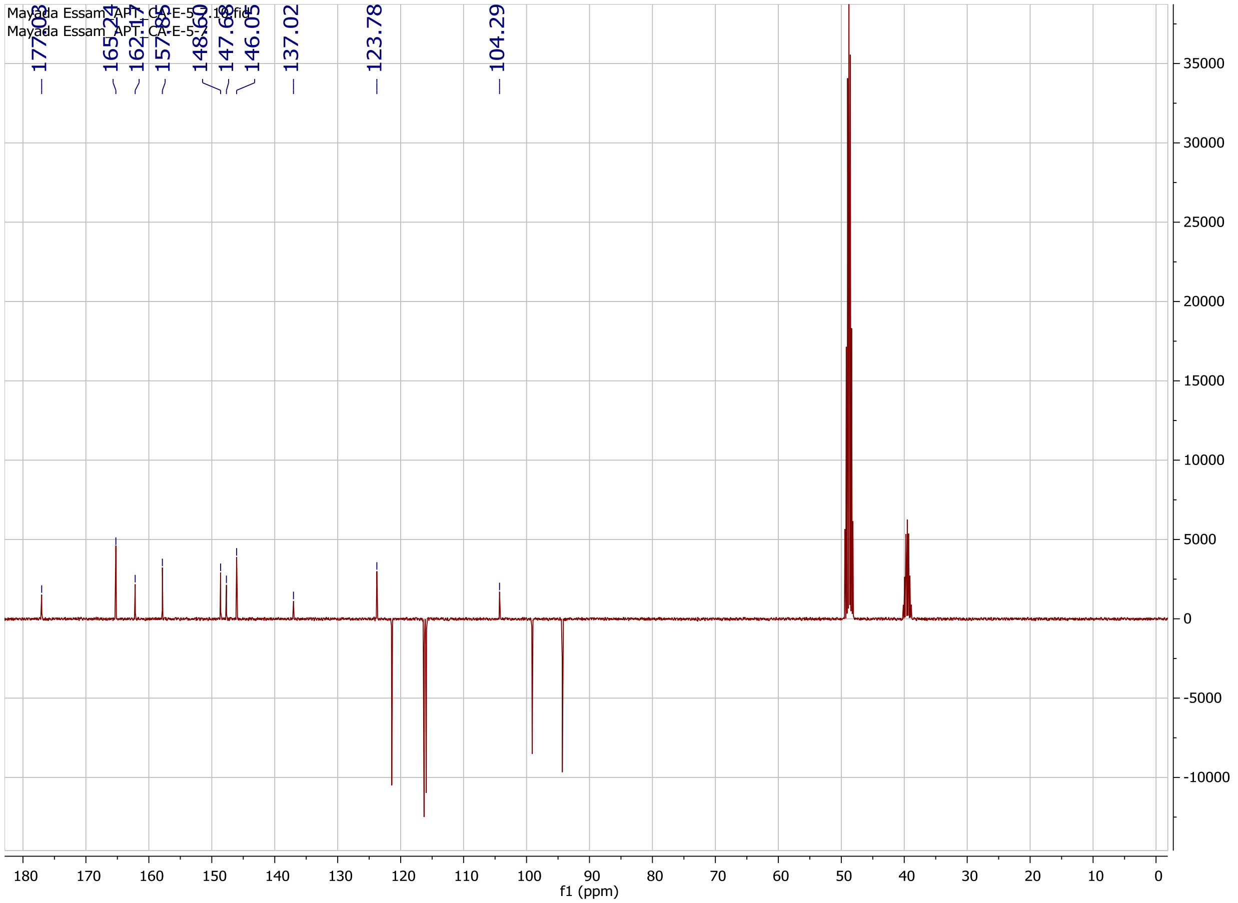

Figure S17. DEPT ^{13}C NMR spectrum of compound **5**

Compound 6 (Kaempferol 3-*O*-glucoside):

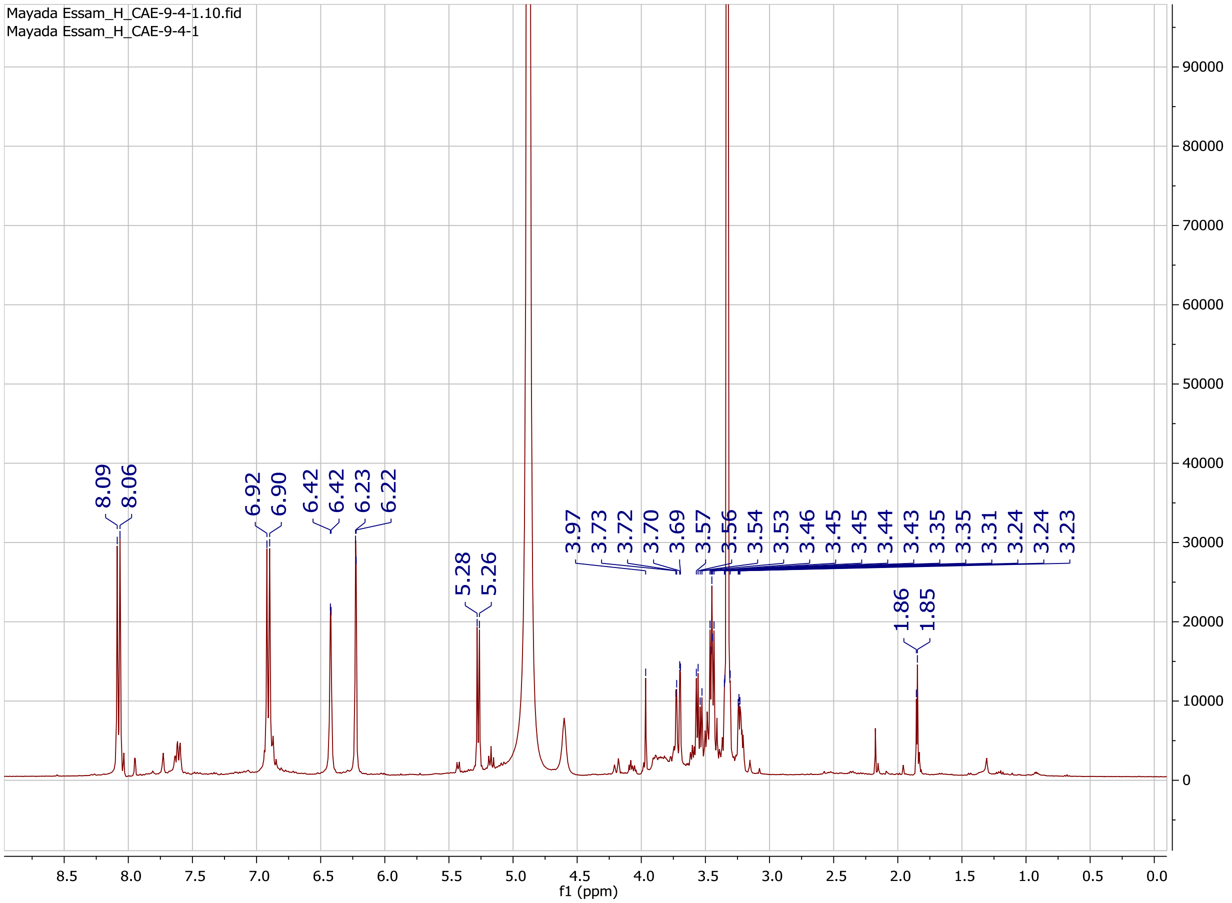

Figure S18. ^{1}H NMR spectrum of compound **6**

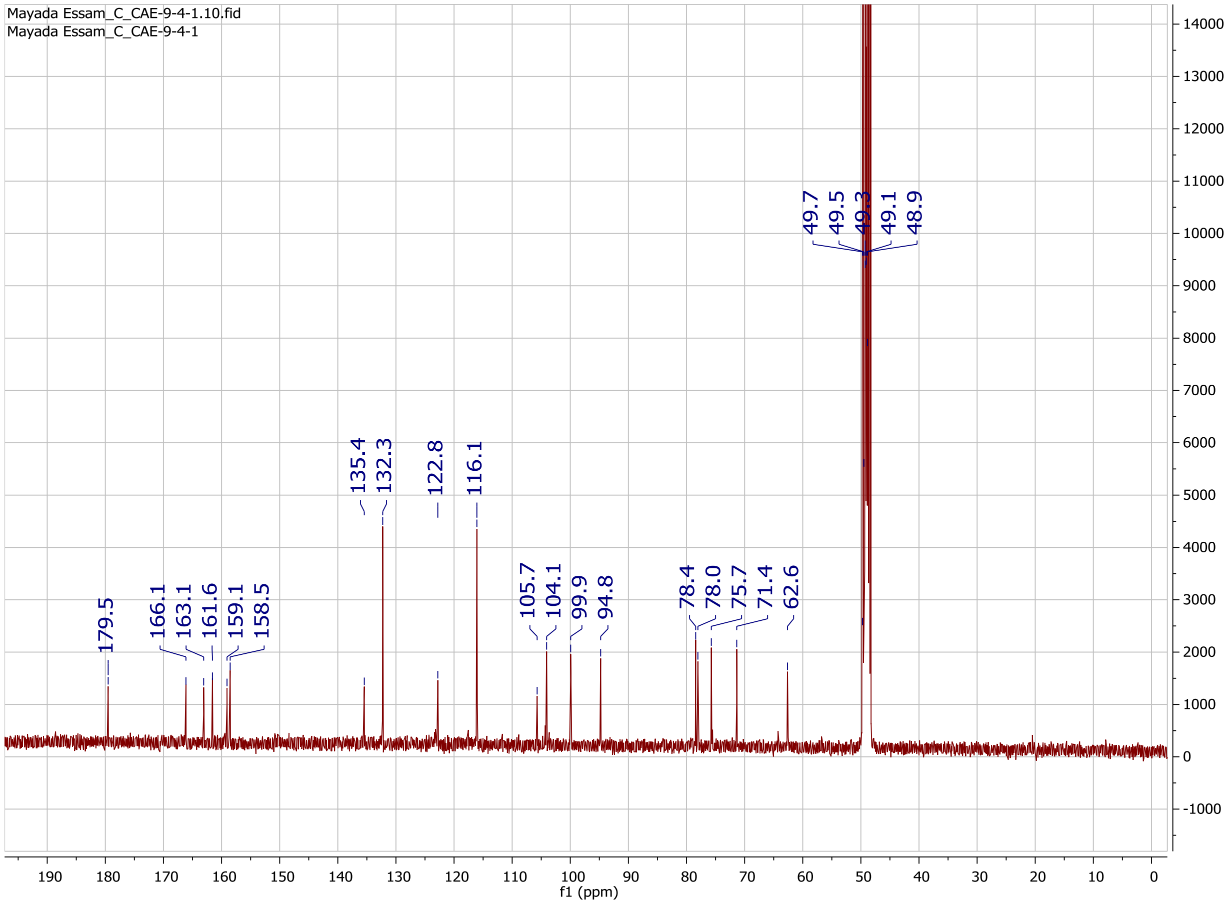

Figure S19. ^{13}C NMR spectrum of compound **6**

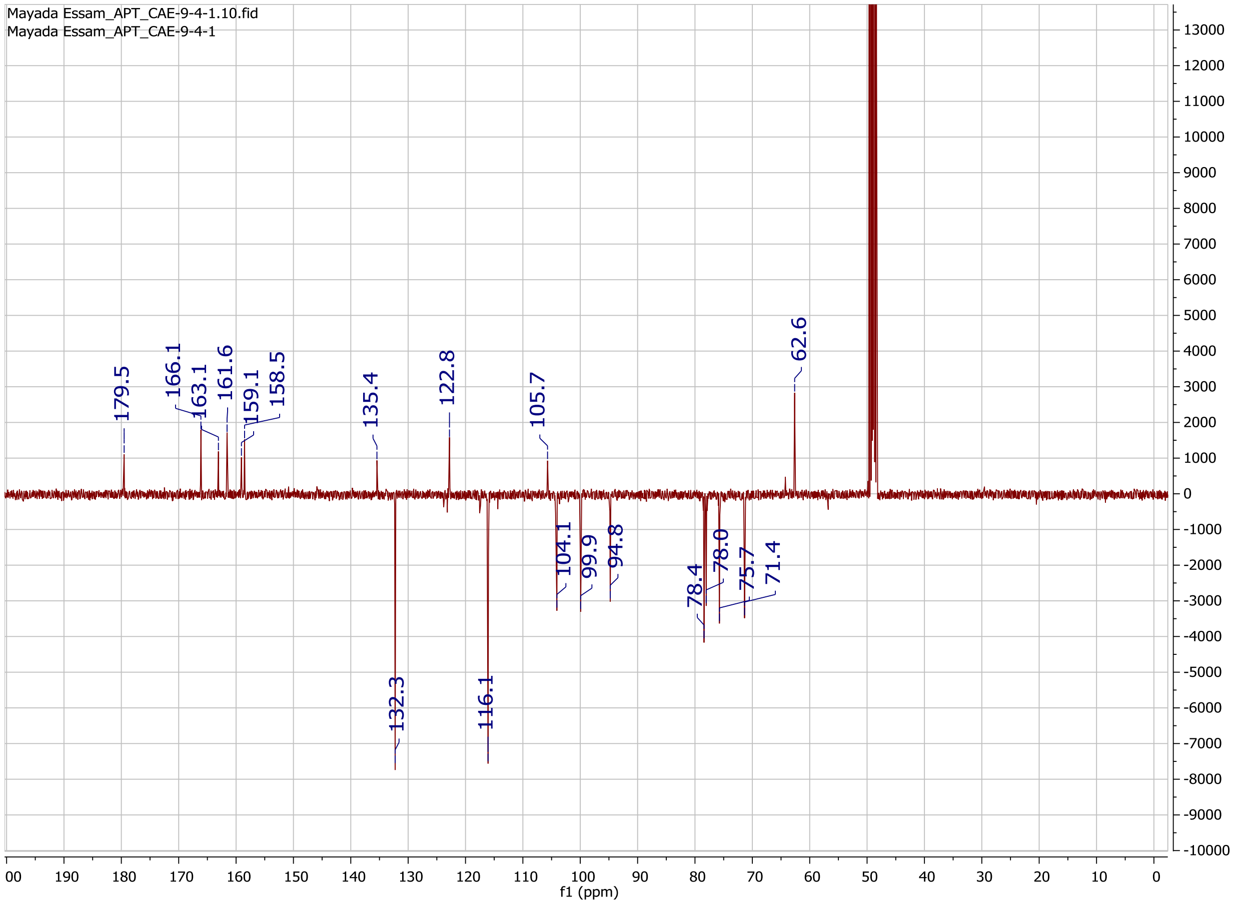

Figure S20. DEPT ^{13}C NMR spectrum of compound **6**

Compound 7 (scopoletin)

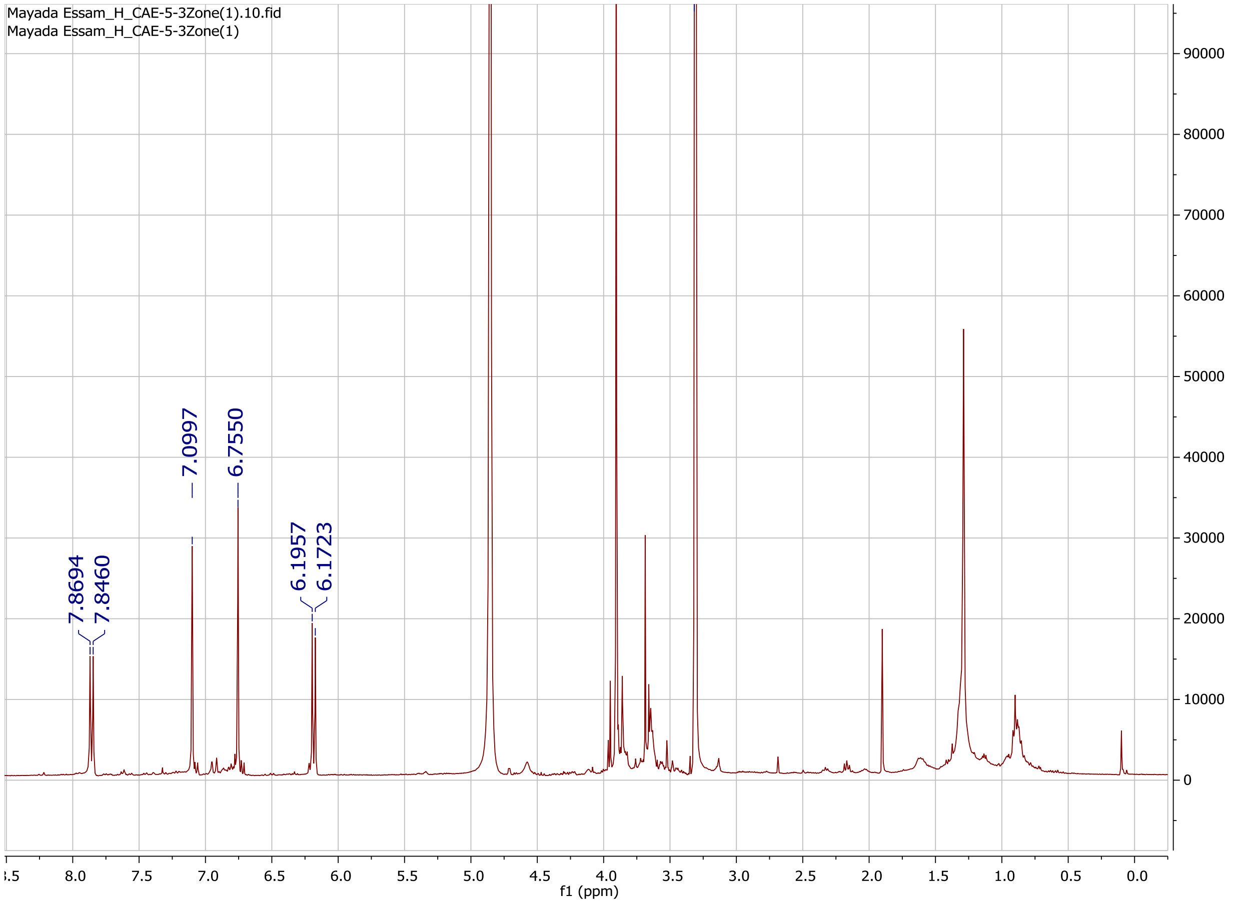

Figure S21. ^{1}H NMR spectrum of compound **7**

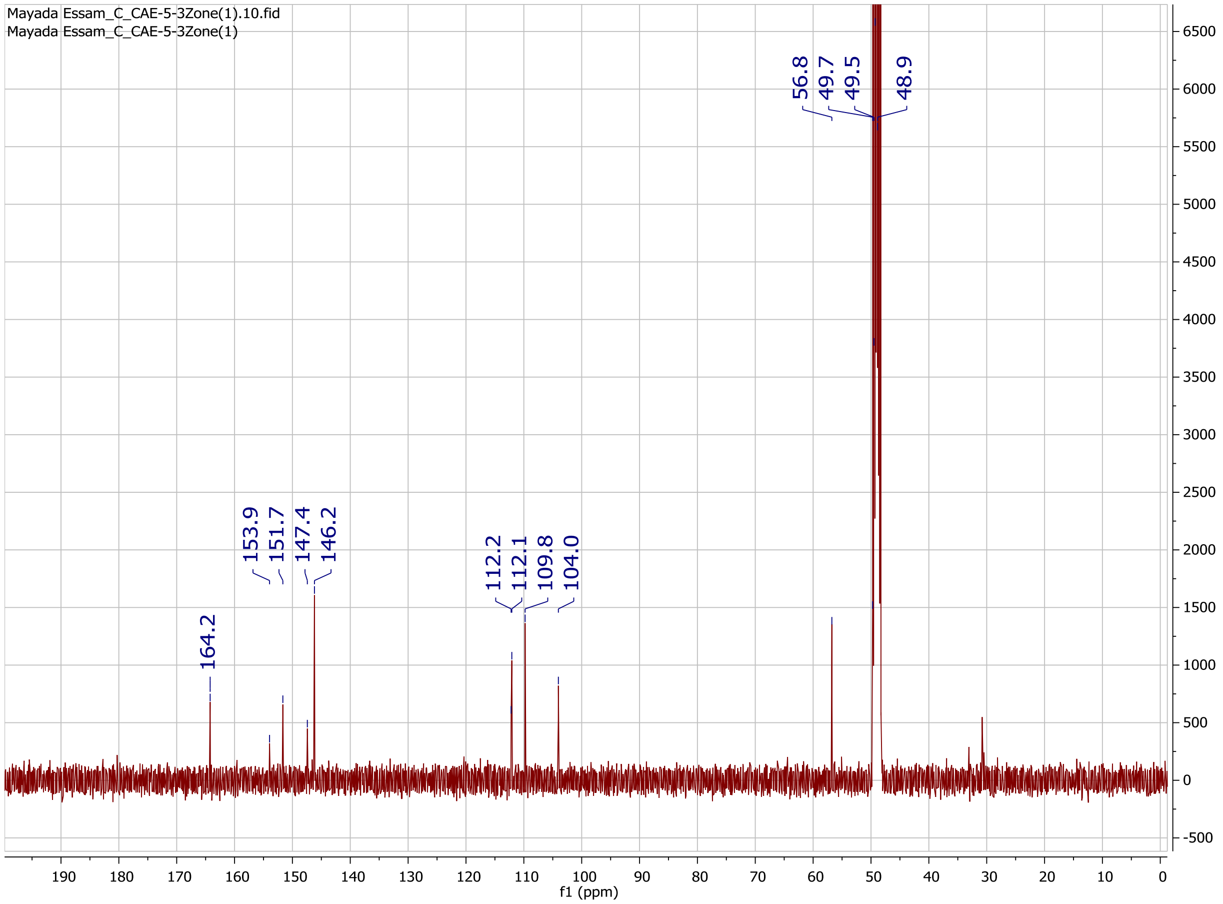

Figure S22. ^{13}C NMR spectrum of compound **7**

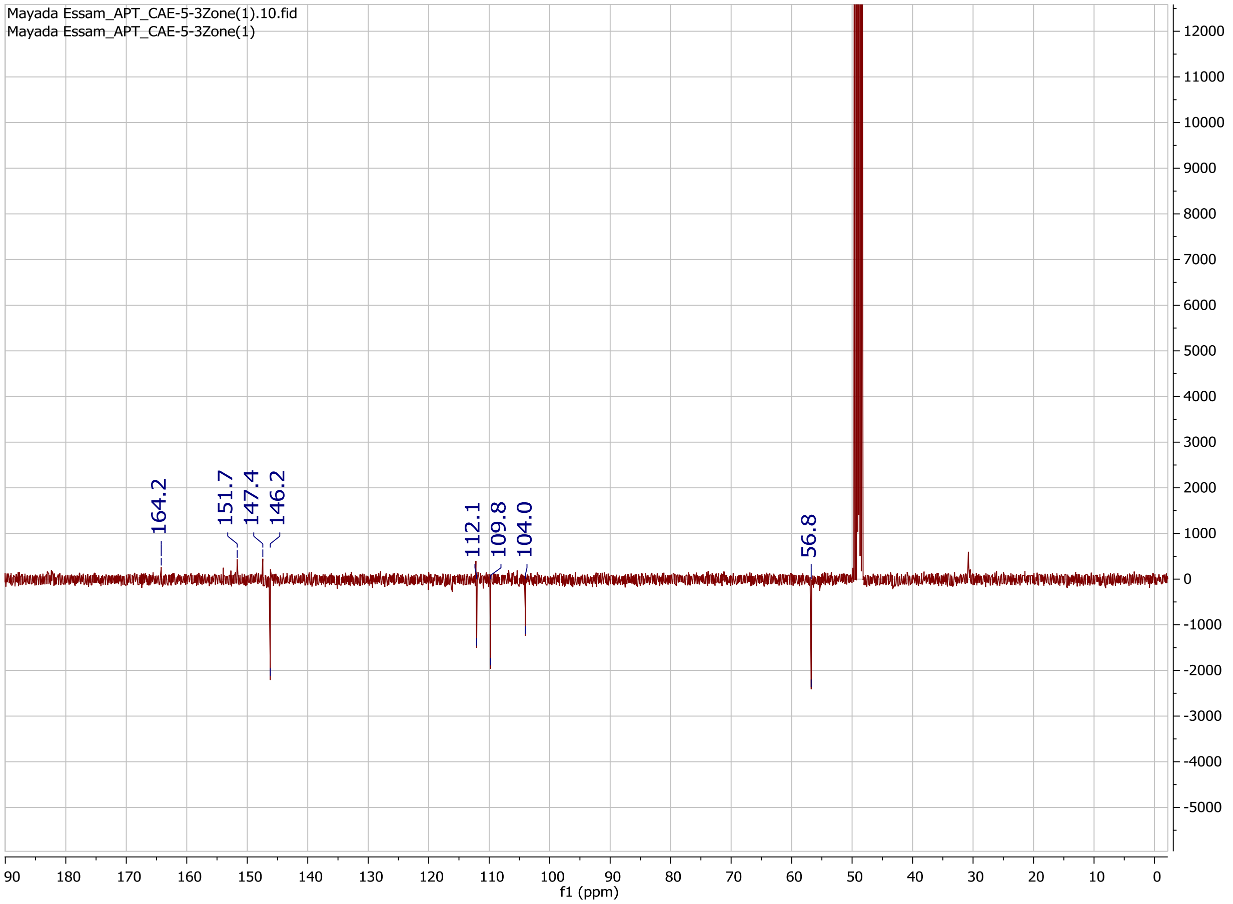

Figure S23. DEPT ^{13}C NMR spectrum of compound **7**

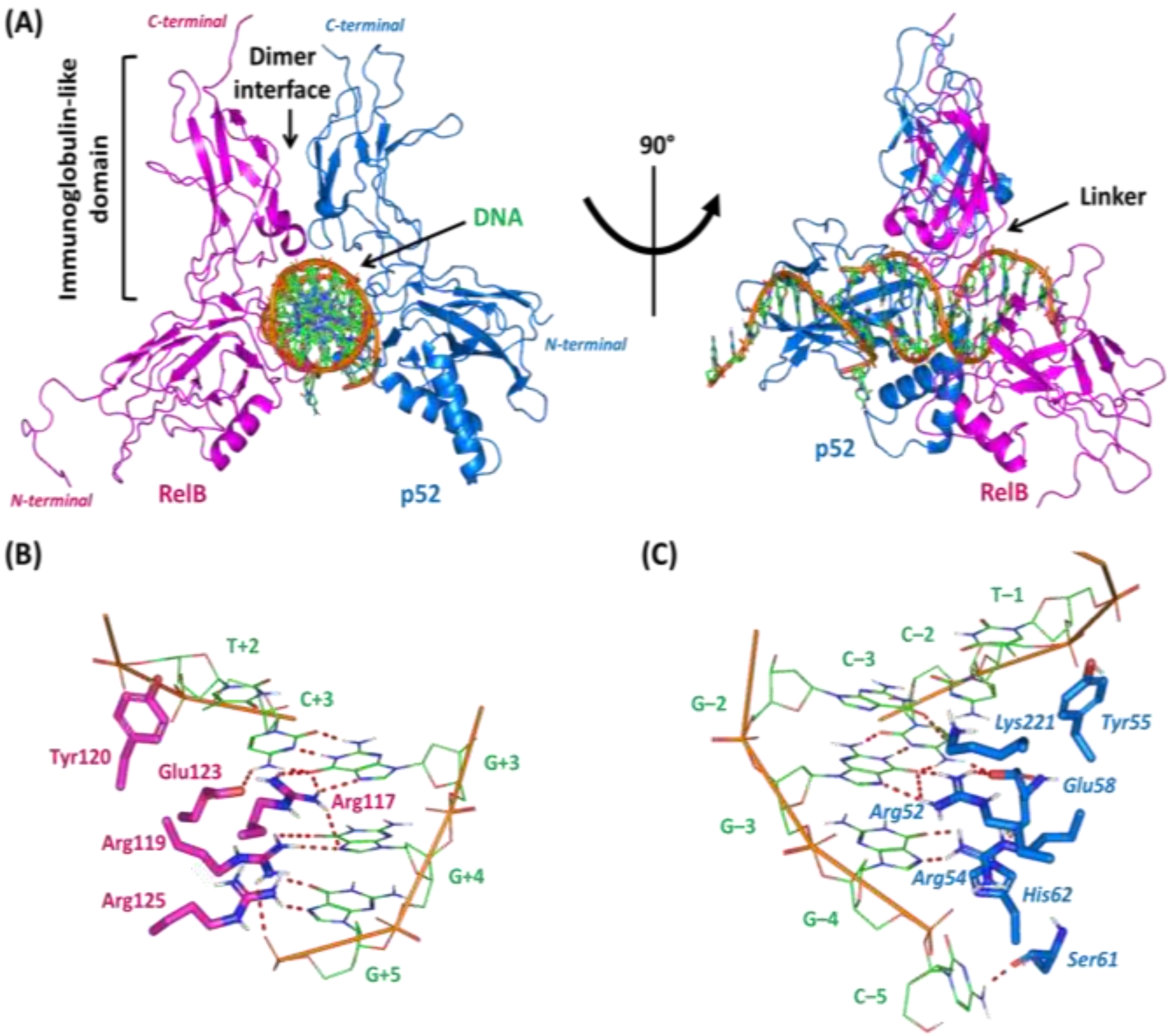

Figure S24. The architecture of NF-κB RelB:p52:DNAheterocomplex. **(A)** Left panel is an overall cartoon representation of NF-κB RelB:p52 heterodimer (PDB: 3DO7) viewed down the helical axis of the bound DNA. The p52 and RelB subunits are illustrated in marine blue and magenta cartoon 3D representations, while the DNA strands are shown in green and orange for the corresponding bases and phosphate backbone, respectively. Right panel is the NF-κB RelB:p52:DNAheterocomplex representation following 90° rotation along its vertical axis. **(B)** Schematic illustration of the detailed contacts between the base-specific residues of the RelB subunit (magenta sticks) and κB DNA-specific bases (green lines). **(C)** Schematic illustration of the detailed contacts between the base-specific residues of the p52 subunit (marine blue sticks) and κB DNA-specific bases (green lines) in addition to a single backbone phosphate group of G+5 (orange line). Polar interactions, represented as hydrogen bonds, are illustrated as red dashed-lines and protein residues are labelled with sequence numbers.

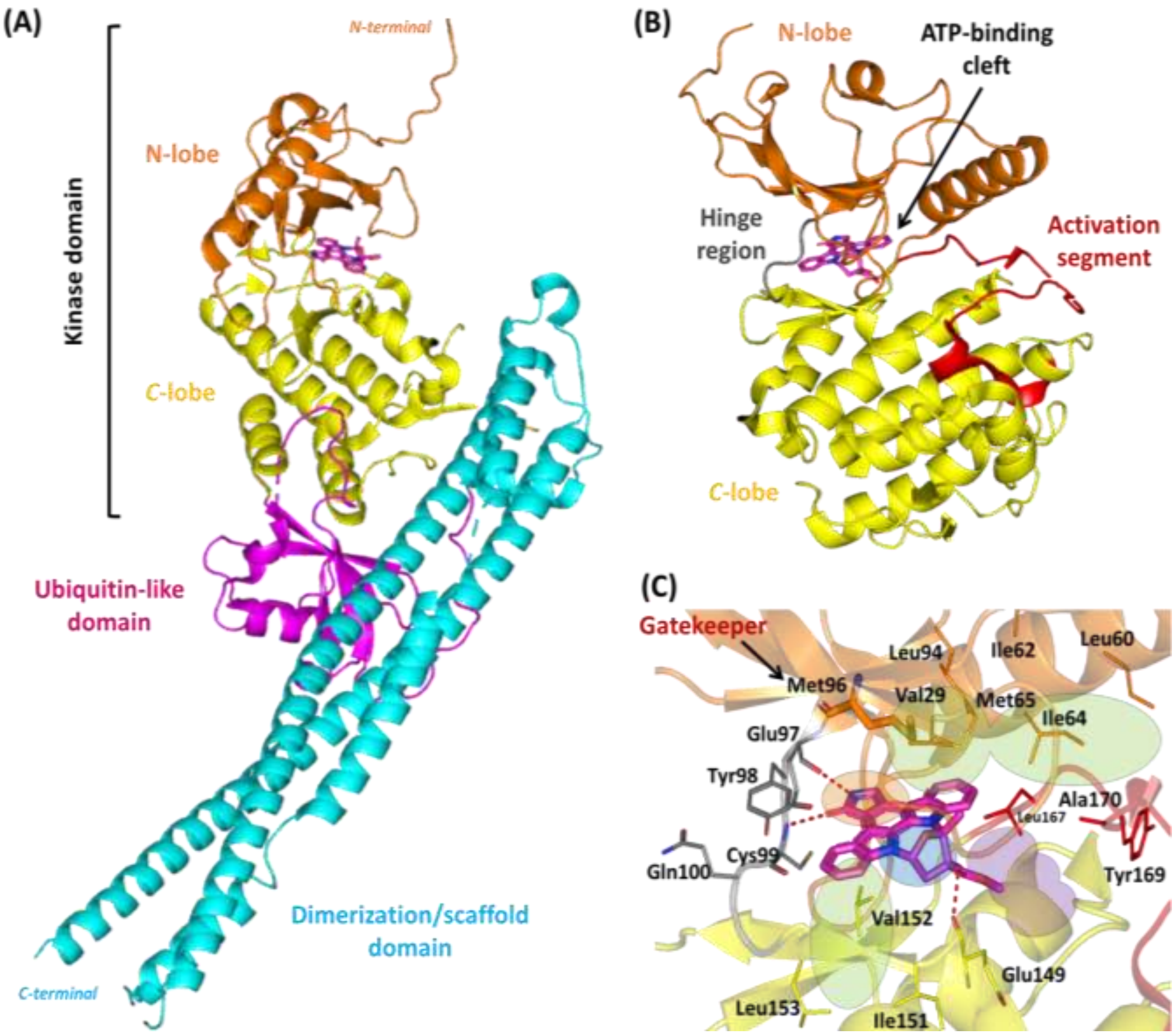

Figure S25. The architecture of human inhibitory-κB kinase β (hIKKβ) in complex with inhibitor. **(A)** Overall cartoon representation of hIKKβ trimodular protein target monomer (PDB: 4KIK) showing its three major domains: the catalytic kinase domain (orange and yellow) with bounded competitive inhibitor KSA (magenta sticks), ubiquitin-like domain (magenta), and elongated scaffold/dimerization domain (cyan). **(B)** Schematic cartoon 3D illustration of the kinase domain in complex with KSA at the ATP-binding site, showing the bilobal domains (orange N-lobe and yellow C-lobe) connected by hinge region (gray) and with activation segment (red). **(C)** Schematic illustration of the detailed contacts between the ATP-binding site key residues (lines) and KSA (magenta sticks) being anchored at the kinase typical subpockets; adenine-specific (orange highlight), sugar-specific (blue highlight), phosphate-specific (violet highlight), hydrophobic pocket (upper green highlight), and hydrophobic channel (lower green highlight). Polar interactions, represented as hydrogen bonds, are illustrated as black dashed-lines and protein residues are labelled with sequence numbers and colored in respect to their position chains.

Table S1. The effect of the crude extract and fractions of *Cynanchum acutum* on fasting blood glucose, insulin and insulin resistance indices in the experimental rats

Groups	FBG (mg/dL)	Insulin (μIU/mL)	HOMA-IR	QUICKI
Normal	91.28 ± 8.35	11.52 ± 2.78	2.60 ± 0.28	0.33 ± 0.03
Control (HFD+STZ)	269.36 ± 28.99*	43.37 ± 4.74*	28.56 ± 3.50*	0.25 ± 0.02*
Crude extract	150.24 ± 15.79$^{*\#}$	27.45 ± 3.10$^{*\#}$	10.00 ± 1.55$^{*\#}$	0.28 ± 0.03$^{*\#}$
Hexane fraction	171.49 ± 18.87$^{*\#}$	32.76 ± 3.79$^{*\#}$	14.01 ± 2.05$^{*\#}$	0.27 ±0.03$^{*\#}$
Chloroform fraction	120.20 ± 12.73$^{*\#}$	22.14 ± 2.90$^{*\#}$	6.57 ± 1.48$^{*\#}$	0.29 ± 0.03$^{*\#}$
Ethyl acetate fraction	106.79 ± 11.80$^{\#}$	16.83 ± 2.88$^{\#}$	4.49 ± 1.09$^{\#}$	0.31 ± 0.03$^{*\#}$
Butanol fraction	238.85 ± 25.69*	38.07 ± 4.72*	22.42 ± 2.98*	0.25 ±0.02*

Data are expressed as mean ± SD and analyzed using one-way ANOVA followed by Bonferroni's post hoe test (n = 6-8). FBG = fasting blood glucose; HOMA-IR = homeostasis model assessment-insulin resistance; QUICKI = quantitative insulin sensitivity check index; *significantly different compared to the normal group at $P< 0.05$; ** at $P<0.01$; ***at $P<0.001$; $^{\#}$significantly different compared to the T2DM control group (high fat diet + Streptozotocin) at $P< 0.05$; $^{\#\#}$ at $P<0.01$; $^{\#\#\#}$at $P<0.001$.

Table S2. The effect of the crude extract and fractions of *Cynanchum acutum* on the body weight, adipose tissue index and lipid profile of the experimental rats

Groups	% increase in body weight	Adipose tissue index %	TG (mg/dL)	TC (mg/dL)	LDL-C (mg/dL)	HDL-C (mg/dL)
Normal	63.70 ± 6.92	2.08 ± 0.31	152.77 ± 19.38	125.27 ± 10.38	69.18 ± 8.36	40.78 ± 4.28
Control (HFD+STZ)	190.20 ± 20.80*	5.40 ± 0.43*	249.59 ± 27.12*	222.47 ± 13.29*	166.83 ± 17.10*	31.29 ± 3.27*
Crude extract	155.80 ± 17.26*#	4.43 ± 0.37*	205.10 ± 21.82*#	168.21 ± 12.38*#	111.09 ± 12.26*#	36.38 ± 3.00#
Hexane fraction	143.64 ± 14.28*#	4.08 ± 0.38*	189.38 ± 18.29*#	177.73 ± 12.37*#	124.36 ± 13.27*#	35.25 ± 2.87*#
Chloroform fraction	156.56 ± 15.39*#	4.45 ± 0.51*	206.07 ± 22.17*#	181.93 ± 14.28*	128.20 ± 14.15*#	33.25 ± 3.28*
Ethyl acetate fraction	136.80 ± 14.27*#	3.89 ± 0.39*#	179.77 ± 20.18*#	155.23 ± 11.36*#	99.41 ± 10.87*#	38.27 ± 2.99#
Butanol fraction	169.48 ± 18.38*	4.82 ± 0.50*	222.80 ± 21.70*	196.34 ± 12.20*	140.16 ± 14.19*	34.26 ± 3.02*

Data are expressed as mean ± SD and analyzed using one-way ANOVA followed by Bonferroni's post hoe test (n = 6-8). TG = triglycerides; TC = total cholesterol; LDL-C = low density lipoprotein-cholesterol; HDL-C = high density lipoprotein-cholesterol; *significantly different compared to the normal group at $P < 0.05$; ** at $P<0.01$; ***at $P<0.001$; #significantly different compared to the T2DM control group (high fat diet + Streptozotocin) at $P < 0.05$; ## at $P<0.01$; ###at $P<0.001$.

Table S3. The effect of the crude extract and fractions of *Cynanchum acutum* on the liver index and liver enzymes of the experimental rats

Groups	Liver index %	ALT (IU/L)	AST (IU/L)
Normal	2.50 ± 0.23	50.20 ± 5.90	68.00 ± 7.10
Control (HFD+STZ)	3.44 ± 0.26*	76.82 ± 8.22*	89.74 ± 9.37*
Crude extract	2.82 ± 0.20#	63.14 ± 7.1*#	72.98 ± 8.01#
Hexane	2.60 ± 0.27#	58.21 ± 6.25#	67.28 ± 7.26#
Chloroform	2.83 ± 0.28#	63.45 ± 7.11*#	73.34 ± 7.77#
Ethyl acetate	2.48 ± 0.25#	55.44 ± 6.00#	64.08 ± 6.99#
Butanol	3.07 ± 0.29*#	68.68 ± 7.25*	79.39 ± 8.67*#

Data are expressed as mean ± SD and analyzed using one-way ANOVA followed by Bonferroni's post hoe test (n = 6-8). ALT = alanine aminotransferase; AST = aspartate aminotransferase; *significantly different compared to the normal group at $P < 0.05$; #significantly different compared to the T2DM control group (high fat diet + Streptozotocin) at $P < 0.05$.

Table S4. The average daily food intake of the experimental rats (g/rat/day) in each group throughout the duration of the study

Days	Normal	Control (HFD+STZ)	C1	C2	C3	C4	C5
1	20.5	25.3	23.5	22.8	21.7	25.0	23.4
2	21.3	24.4	22.9	21.9	21.9	25.1	24.3
3	22.2	23.1	21.4	23.4	23.5	24.3	22.5
4	20.7	25.0	22.6	21.8	22.8	23.8	23.3
5	22.8	25.2	23.8	22.3	22.7	22.9	21.9
6	21.9	23.4	24.7	21.7	21.9	22.8	22.5
7	20.9	23.4	22.5	24.5	23.0	22.9	22.7
8	21.2	24.9	23.7	25.0	23.7	23.3	21.9
9	22.0	24.3	24.2	24.1	21.7	24.0	25.0
10	20.3	23.7	25.3	23.4	22.4	22.8	23.5
11	22.3	22.2	21.5	22.7	22.6	23.5	22.9
12	21.5	24.4	21.4	22.9	22.5	22.7	24.3
13	20.9	23.1	22.8	21.8	21.7	24.5	22.9
14	21.2	24.4	23.5	23.7	23.4	24.2	22.5
15	22.8	25.2	21.9	24.4	23.5	23.4	23.5
16	20.2	24.4	24.1	21.9	24.2	22.8	22.8
17	21.7	25.0	21.8	23.5	23.6	24.3	24.4
18	22.3	23.9	22.5	24.7	21.8	23.8	22.7
19	20.9	24.1	23.3	24.4	23.5	22.7	21.9
20	22.8	24.0	24.7	22.8	22.8	21.9	22.5
21	20.6	24.3	22.7	21.8	22.5	22.7	23.7

C1: Rutin; C2: Quercetin 3-O-neohesperdoside; C3: Quercetin-3-O- β-galactoside; C4: Isoquercetin; C5: Quercetin.

Table S5. The *In-silico* findings of ligand-docking studies at the DNA-specific binding domain of the NF-κB RelB:p52 heterodimer

**Ligand name**	**MOE docking Score (*S*) [a] Kcal/mol**	**RMSD_refine [b] Å**	**Ligand-target polar interaction (Hydrogen bonding) description [Type; Length Å; Angle °; ligand functionality/Binding Residues]**
**Quercetin-3-*O*-*β*-galactoside**	-6.98 ± 0.22 (RelB)<br>-6.06 ± 0.06 (p52)	1.79 ± 0.03	2.4 ± 0.11 **Å ;** 171.0 ± 0.18 ° **;** Tyr120-**O**H / sugar C6-O**H**<br>2.7 ± 0.19 **Å ;** 119.8 ± 0.74 ° **;** Asn242-C=**O** sidechain / sugar C4-O**H**<br>3.0 ± 0.05 **Å ;** 139.7 ± 0.44 ° **;** Arg117-=N^{+}H**H** / **O** linker<br>2.3 ± 0.23 **Å ;** 126.3 ± 0.32 ° **;** *Arg52*-NH**H** / Ph-*para*-O**H**<br>2.0 ± 0.55 **Å ;** 121.7 ± 0.07 ° **;** *Ser220*-**O**H / sugar C2-O**H**
**Quercetin**	-5.96 ± 0.58 (RelB)<br>-5.13 ± 0.84 (p52)	1.53 ± 0.29	1.6 ± 0.44 **Å ;** 136.2 ± 0.23 ° **;** Tyr120-**O**H / sugar Ph-*meta*-O**H**<br>2.5 ± 0.34 **Å ;** 122.0 ± 0.56 ° **;** Tyr120-**O**H / sugar Ph-*para*-O**H**<br>2.8 ± 0.01 **Å ;** 139.0 ± 0.77 ° **;** Ser275-**O**H / **O1** of chromone ring<br>2.2 ± 0.45 **Å ;** 112.2 ± 0.89 ° **;** *Ser188*-O**H** / Ph-*para*-**O**H<br>2.2 ± 0.78 **Å ;** 169.3 ± 0.33 ° **;** *Ser222*-**O**H / Ph-*para*-O**H**
**Isoquercitrin**	-4.90 ± 0.34 (RelB)<br>-4.12 ± 0.14 (p52)	1.98 ± 0.11	1.8 ± 0.63 **Å ;** 123.9 ± 0.93 ° **;** Tyr120-**O**H / sugar C6-O**H**<br>2.4 ± 0.94 **Å ;** 164.9 ± 0.88 ° **;** Lys274-C=**O** / sugar C2-O**H**<br>2.0 ± 0.09 **Å ;** 164.0 ± 0.64 ° **;** *Tyr55*-**O**H / sugar C3-O**H**<br>2.6 ± 0.19 **Å ;** 104.9 ± 0.73 ° **;** *Ser222*-**O**H / sugar C2-O**H**
**Rutin**	-7.47 ± 0.22 (RelB)<br>-7.01 ± 0.66 (p52)	1.46 ± 0.77	3.3 ± 1.01 **Å ;** 124.5 ± 0.92 ° **;** Arg117-=N^{+}H**H** / glucopyranose C3-**O**H<br>2.3 ± 0.52 **Å ;** 159.9 ± 0.81 ° **;** Lys210-N**H** / Ph-*para*-**O**H<br>2.4 ± 0.92 **Å ;** 140.8 ± 0.84 ° **;** Glu238-C=**O** sidechain / chromone C7-O**H**<br>2.0 ± 0.16 **Å ;** 134.7 ± 0.11 °**;** Asn242-C=**O** sidechain /rhamnopyranose C2-O**H**<br>2.6 ± 0.81 **Å ;** 143.6 ± 0.89 ° **;** Lys274-C=**O** / rhamnopyranose C4-O**H**<br>3.2 ± 0.77 **Å ;** 126.0 ± 0.89 ° **;** *Arg52*-=N^{+}H**H** / chromone C7-**O**H<br>2.0 ± 0.34 **Å ;** 119.8 ± 0.83 ° **;** *Tyr55*-**O**H / rhamnopyranose C2-O**H**<br>2.2 ± 0.39 **Å ;** 120.8 ± 0.09 ° **;** *Lys143*-N^{+}H$_2$**H** / glucopyranose C3-O**H**<br>2.4 ± 0.61 **Å ;** 137.0 ± 0.71 ° **;** *Ser188*-**O**H / rhamnopyranose C4-O**H**<br>1.9 ± 0.34 **Å ;** 108.6 ± 0.44 ° **;** *Lys221*-N^{+}H$_2$**H** / rhamnopyranose C2-**O**H
**Quercetin-3-neo-hesperidoside**	-6.32 ± 0.46 (RelB)<br>-5.16 ± 0.22 (p52)	1.91 ± 0.63	2.5 ± 0.29 Å **;** 129.4 ± 0.92 ° **;** Tyr120-O**H** / glycosidic **O** linker<br>2.0 ± 0.31 Å **;** 119.7 ± 0.27 °**;** Asp272-C=**O** sidechain /rhamnopyranose C4-O**H**<br>2.9 ± 0.45 **Å ;** 124.7 ± 0.11 ° **;** Tyr55-**O**H / rhamnopyranose C2-O**H**<br>2.1 ± 0.43 **Å ;** 147.1 ± 0.21 ° **;** *Ser188*-**O**H / rhamnopyranose C4-O**H**

[a] Scored for the best-ranking pose chosen based on visual inspection, MOE (***S***) scoring function and Root-mean-squared-deviation (RMSD). Refinment via Rescoring by GBVI/WSA dG scoring function.
[b] RMSD between heavy atoms of predicted binding mode (following refinement) and prior refinement.

Table S6: The *In-silico* findings of ligand-docking studies at the ATP-binding domain of the *h*IKK protein target

**Ligand name**	**MOE docking Score (*S*)** [a] **Kcal/mol**	**RMSD_refine** [b] **Å**	**Ligand-target polar interaction (Hydrogen bonding) description [Type; Length Å; Angle °; ligand functionality/Binding Residues]**
**KSA (redocking)**	-6.93 ± 0.79	0.80 ± 0.12	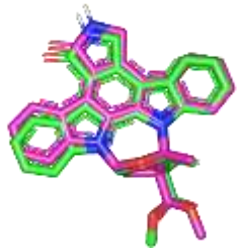<br>2.0 ± 0.71 **Å ;** 164.7 ± 0.71 ° **;** Glu97-C=**O** mainchain / pyrrolidin N**H**<br>1.9 ± 0.82 **Å ;** 165.7 ± 0.18 ° **;** Cys99-N**H** / pyrrolidin C=**O**<br>3.2 ± 0.12 **Å ;** 142.4 ± 0.34 ° **;** Glu149-C=**O** mainchain / terminal O**H**
**Quercetin-3-*O*-*β*-galactoside**	-7.91 ± 0.52	1.15 ± 0.34	2.9 ± 0.72 **Å ;** 145.4 ± 0.17 ° **;** Lys44-N**H** / chromone C7-O**H**<br>2.8 ± 0.91 **Å ;** 120.2 ± 0.45 ° **;** Cys99-N**H** / Ph-*meta*-O**H**<br>3.1 ± 0.40 **Å ;** 126.2 ± 0.91 ° **;** Cys99-N**H** / Ph-*para*-**O**H<br>2.4 ± 0.82 **Å ;** 163.7 ± 0.74 ° **;** Asp103-C=**O** mainchain / sugar C2-O**H**<br>2.7 ± 0.71 **Å ;** 127.5 ± 0.33 ° **;** Asp103-C=**O** mainchain / sugar C3-O**H**<br>2.5 ± 0.53 **Å ;** 142.3 ± 0.13 ° **;** Asp103-C=**O** mainchain / sugar C4-O**H**<br>3.0 ± 0.17 **Å ;** 116.1 ± 0.34 °**;** Glu149-C=**O** mainchain / chromone enolic O**H**<br>2.5 ± 0.45 **Å ;** 148.8 ± 0.27 ° **;** Asp166-C=**O** sidechain C7-O**H**
**Isoquercitrin**	-6.28 ± 0.61	1.98 ± 1.21	2.3 ± 0.61 **Å ;** 108.7 ± 0.33 ° **;** Glu97-C=**O** mainchain / chromone C7-O**H**<br>3.1 ± 0.32 **Å ;** 126.1 ± 0.44 ° **;** Cys99-N**H** / chromone C7-**O**H<br>2.6 ± 0.12 **Å ;** 139.9 ± 0.01 ° **;** Asp103-C=**O** sidechain / sugar C2-O**H**<br>2.0 ± 0.22 **Å ;** 156.1 ± 0.05 ° **;** Asp103-C=**O** sidechain / sugar C3-O**H**
**Rutin**	-6.73 ± 0.53	2.09 ± 0.82	2.1 ± 0.29 **Å ;** 130.8 ± 0.72 ° **;** Cys99-N**H** / Ph-*para*-**O**H<br>3.4 ± 0.91 **Å ;** 111.0 ± 0.31 ° **;** Asp103-N**H** mainchain / Ph-*meta*-**O**H<br>2.7 ± 0.83 **Å ;** 129.2 ± 0.34 ° **;** Asp103-C=**O** sidechain / Ph-*meta*-O**H**<br>3.4 ± 0.64 **Å ;** 144.1 ± 0.15 ° **;** Asn109-N**H** sidechain / rhamnopyranose C3-**O**H<br>3.3 ± 0.15 **Å ;** 109.8 ± 0.12 ° **;** Asp166-N**H** mainchain / chromone C7-O**H**
**Quercetin-3-neo-hesperidoside**	-5.94 ± 0.55	2.52 ± 0.43	2.2 ± 0.18 **Å ;** 127.0 ± 0.45 ° **;** Glu97-C=**O** mainchain / chromone C7-O**H**<br>2.8 ± 0.19 **Å ;** 173.9 ± 0.19 ° **;** Cys99-C=**O** mainchain / Ph-*meta*-O**H**<br>2.7 ± 0.28 **Å ;** 154.5 ± 0.05 ° **;** Asp103-C=**O** sidechain / galactopyranose C3-O**H**<br>2.5 ± 0.37 **Å ;** 125.2 ± 0.83 ° **;** Asp103-C=**O** sidechain / galactopyranose C4-O**H**<br>3.5 ± 0.44 **Å ;** 134.1 ± 0.29 ° **;** Lys106-N**H** sidechain / Ph-*para*-**O**H
**Quercetin**	-6.57 ± 0.71	1.04 ± 0.53	3.3 ± 0.81 **Å ;** 129.8 ± 0.43 ° **;** Asn28-N**H** sidechain / Ph-*para*-**O**H<br>3.6 ± 0.94 **Å ;** 121.9 ± 0.21 ° **;** Asn28-C=**O** sidechain / Ph-*para*-O**H**<br>3.3 ± 0.92 **Å ;** 137.4 ± 0.43 ° **;** Cys99-N**H** mainchain / chromone C5-**O**H

			2.3 ± 0.84 **Å ;** 153.4 ± 0.91 ° **;** Cys99-C=**O** mainchain / chromone C5-O**H**<br>3.3 ± 0.65 **Å ;** 129.8 ± 0.45 ° **;** Asp166-N**H** mainchain / Ph-*meta*-**O**H

[a] Scored for the best-ranking pose chosen based on visual inspection, MOE (***S***) scoring function and Root-mean-squared-deviation (RMSD). Refinment via Rescoring by GBVI/WSA dG scoring function.

[b] RMSD between heavy atoms of predicted best-ranking binding mode as compared to the crystal ligand, KSA.
